# Supplementary material for: Safe and just Earth system boundaries
Source: Nature. 2023 May 31;619(7968):102–11. doi: 10.1038/s41586-023-06083-8 (PMC10322705; doi:10.1038/s41586-023-06083-8)
Supplement: Supplementary file 1 — Supplementary Methods, Figs. 1–3 and Tables 1–11. [file 41586_2023_6083_MOESM1_ESM.pdf]

nature portfolio
https://doi.org/10.1038/s41586-023-06083-8

Supplementary information

Safe and just Earth system boundaries

In the format provided by the
authors and unedited

SUPPLEMENTARY METHODS for '*Safe and just Earth system boundaries'*

including SUPPLEMENTARY FIGURES S1-S3 AND SUPPLEMENTARY TABLES S1-S11

Here we provide technical details of the methods and evidence used to specify safe and just Earth system boundaries (ESBs). We begin with a *glossary of key terms*.

3Is: Within our Earth system justice framework (Gupta et al. 2023), we use three Earth system justice criteria (the "3Is") to assess whether adhering to the safe ESBs could protect people from significant harm:

- '**Interspecies justice and Earth system stability**' (I1) (Burke and Fishel 2020)
- **Intergenerational justice** (Meyer 2021) between past and present generations (I2a) and present and future generations (I2b);
- **Intragenerational justice** (I3) between countries (Blake and Smith 2022), communities and individuals through an intersectional lens (Norlock 2019).

Earth system: The energetically open but nearly materially closed system of all living and non-living (abiotic) interacting things including forcings, feedbacks and biological processes at the surface of the Earth, bounded by outer space on the outside, and by the inner Earth (with its own heat source) on the inside (Steffen et al. 2020). The Earth system is a **social-ecological system,** a coupled system of humans and nature (Berkes et al. 2000; Liu et al. 2007).

- **Earth system boundaries (ESBs):** See safe and just ESB definitions below. Our approach goes beyond planetary boundaries in both combining elements from local to global levels and biophysical and social science knowledge domains.
- **Earth system change:** Change in the state or other properties of one or more sub-systems of the Earth system.
- **Earth system domain:** A sub-system, component or process within the Earth system whose boundaries we assess, e.g. the climate system, nitrogen cycle, water cycle, biosphere.
- **Earth system justice**: Building on recognition and epistemic justice and local to global justice scholarship, it includes procedural justice (access to information, decision-making, civic space and courts) and substantive justice in terms of access to basic resources and services and allocation of the remaining resources, risks, and responsibilities. These elements are applied in terms of ends (just minimum access and no significant harm) and means (addressing drivers of Earth system change and vulnerability, addressing barriers to change, liability and revisiting allocation mechanisms) (Gupta et al. 2023).
- **Earth system resilience/stability:** the capacity of the Earth system to deal with change and continue to function. In this manuscript, we primarily analyse the resilience/stability of the biophysical components and processes of the Earth system.

Exposure: "The presence of people; livelihoods; species or ecosystems; environmental functions, services, and resources; infrastructure; or economic, social, or cultural assets in places and settings that could be adversely affected" (IPCC 2022) in situ or through interconnections.

Global commons: We define the global commons as those domains and natural functions that regulate the stability and resilience of the Earth system, such as the ecosystems, biosphere, hydrosphere (oceans and freshwater), cryosphere, lithosphere, major biomes, large scale biophysical systems and processes

(e.g. the carbon cycle, freshwater cycle) and tipping elements (Nakicenovic et al. 2016). The stability and resilience of the Earth system is vital to all. This stability and resilience is dependent upon both the global commons as recognized under international law (the areas of the planet outside of national jurisdiction such as oceans, atmosphere and outer space) and also those systems and functions inside national jurisdiction, including tipping elements that regulate Earth System stability to safeguard our livelihoods, for example rainforests or sea ice.

Harm: negative impact on humans, communities and countries from Earth system change *additional* to background rates and to changes in vulnerability. For the purposes of this manuscript, we analytically separate matters of harm due to Earth system change from matters of sufficient access to natural resources (Rammelt et al. 2022).

- **Significant harm:** What constitutes significant harm is difficult to quantify. A "leave no one behind" approach (UN GA 2015) would judge significant as no deaths additional to background rates; others see 10 dead and 100 displaced as a sign of a disaster (IFRC 2020), 1 in 10000 to 1 in a million is a common norm in cancer research (Dankovic and Whittaker 2015), while the IPCC sees 10-100 million exposed as severe to high reasons for concern (O'Neill, van Aalst, Zaiton Ibrahim, et al. 2022). Within the Earth Commission we decided not to define on a cut off point for significant harm and instead define significant harm as: Existential and/or irreversible negative impact on countries, communities or people, such as substantial loss of life, livelihood and income, loss of access to nature's contributions to people, loss of land, chronic disease, injury, malnutrition and displacement (Gupta et al. 2023).

- **No Significant Harm (NSH) Principle** - States and other actors responsible for anthropogenic Earth system change have a duty to refrain from causing significant harm; to prevent, reduce, and control the risk of causing significant harm; and to repair or compensate for the significant harm already inflicted.

- **Just (NSH) ESB** - Earth system state that minimises risk of significant harm to present and future generations, countries, communities, and people

(Largely intact) natural ecosystem area: Largely intact ecosystems are those where the ecological function and species composition largely resembles those of undisturbed ecosystems. Light human use that does not significantly affect these functions, for example low density population subsistence use by indigenous communities, is not excluded.

Safe Earth system boundaries: Safe Earth system boundaries are those where biophysical stability of the Earth system is maintained and enhanced over time, thereby safeguarding its functions and ability to support humans and all other living organisms (Rockström et al. 2021). We set safe boundaries at global and/or sub-global scales, depending on the domain.

Science-based targets: "Measurable, actionable, and time-bound objectives, based on the best available science, that allows actors to align with Earth's limits and societal sustainability goals" (SBTN 2020).

Tipping elements: Those components or processes that regulate the functioning and state of the planet and that show evidence of having thresholds at which small additional perturbations can trigger self-reinforcing changes that undermine Earth system resilience (Lenton et al. 2008; Armstrong McKay et al. 2022).

Transformation: “The altering of fundamental attributes of a system (including value systems; regulatory, legislative, or bureaucratic regimes; financial institutions; and technological or biological systems)” (IPCC 2012). They are a systemic, non-incremental and “fundamental qualitative change… that often involves a change in paradigm and may include shifts in perception and meaning, changes in underlying norms and values, reconfiguration of social networks and patterns of interaction, changes in power structures, and the introduction of new institutional arrangements and regulatory frameworks” (IPCC 2012).

(Cross-scale) translation: Linking of ESBs to actor-specific shares of resources and responsibilities (Bai et al. 2022).

Vulnerability: “the propensity or predisposition to be adversely affected” (IPCC 2022). It encompasses a variety of concepts and elements including sensitivity or susceptibility to harm, exposure, and lack of capacity to cope and adapt.

1. Safe Earth system boundaries

In this section, we describe the evidence base for our safe Earth system boundaries. We also briefly remark how each domain's analysis relates to our I1 "Interspecies justice and Earth system stability" justice criterion (see section 2 for further information).

1.1 Climate & Cryosphere

Our synthesis of existing knowledge of climate tipping elements and their temperature thresholds is summarised in Figure S1. We combine this with knowledge of biosphere functioning, temperature variability in the recent geological past, and the IPCC's most recent 'Reasons For Concern' risk levels to arrive at the summary of the Earth Commission's quantification for the climate change safe limits in Table S1.

These results are also our inductive application of the justice criteria of "interspecies justice and Earth system stability" (I1). We have focused more on the Earth system stability component than on interspecies justice in the specific case of climate change.

Table S1. *Summary of the Earth Commission's quantification of the safe limits for climate change and the cryosphere based on* ***A)*** *the likelihood of reaching climate tipping points (Armstrong McKay et al. 2022);* ***B)*** *Quaternary temperature ranges (Marcott et al. 2013; Willeit et al. 2019; Kaufman et al. 2020; Fox-Kemper, Hewitt, Xiao, et al. 2021; Osman et al. 2021);* ***C)*** *the state of key carbon sinks e.g. Amazon & Tundra (Hubau et al. 2020; de Vrese et al. 2021; Gatti et al. 2021; Winkler et al. 2021), comparisons of the impacts of 1.5 and 2°C in SR1.5 (Schleussner et al. 2016; Hoegh-Guldberg, Jacob, Taylor, et al. 2018), the AR6 Terrestrial & Freshwater Ecosystems Key Risks (TFEKRs) (Parmesan, Morecroft, Trisurat, et al. 2022), and climate-induced localised tipping points in the ocean (Heinze et al. 2021); and* ***D)*** *IPCC AR6's Reasons For Concern (RFCs) (O'Neill, van Aalst, Zaiton Ibrahim, et al. 2022).*

**GMST (vs. 1850-1900)**	**Description**
**<0.5°C**	A. Negligible likelihood of passing tipping points<br>B. Within maximum Holocene natural variability range<br>C. Carbon sinks working at full capacity, no TFEKRs reached<br>D. RFC1 moves towards moderate at 0.4°C
**0.5-1.0°C**	A. Low likelihood of passing tipping points<br>B. Exiting maximum Holocene range<br>C. Some carbon sinks peaking, TFEKRs moderate by 0.8°C<br>D. RFC1-2/5 moderate at ~0.5/0.9°C, RFC1 high at ~0.9°C
**1.0-1.5°C**	A. Moderate likelihood of passing tipping points<br>B. Outside of Holocene but within Interglacial range<br>C. Some carbon sinks weakening, TFEKRs moderate, ocean impacts already evident<br>D. RFC4 moderate at ~1.3°C, RFC2 high at ~1.2°C, RFC1 moves towards very high from 1.2°C
**1.5-2.0°C**	A. High likelihood of passing tipping points<br>B. Likely exiting Interglacial range<br>C. Some carbon sinks transitioning to sources, TFEKRs high by 2°C, critical thresholds in ocean ecosystems expected at >1.5°C, terrestrial species range loss & ecosystem transformations c. doubled at 2°C vs. 1.5°C<br>D. RFC1 very high at ~1.5°C, RFC2 moves towards very high from

	1.8°C, RFC4/5 moves towards high from 1.5°C
**>2.0°C**	A. Very high likelihood of passing tipping points<br>B. Warmest since mid-Pliocene (~3 million years ago)<br>C. Widespread weakening of land carbon sinks, TFEKRs move towards very high from 2.1°C, higher impacts on ecosystems<br>D. RFC2 very high at 2°C, RFC4/5 high at 2°C & move towards very high from 2.5°C

Our climate change safe limits are based on a) the likelihood of passing key climate tipping points, b) the past temperature range of the Holocene (<0.5-1°C) and prior Pleistocene interglacials such as the Eemian (<1.5-2°C), and c) non-tipping Earth system impacts affecting biosphere functioning (e.g. biodiversity decline, biome shifts, and the operation of natural carbon sinks). The climate tipping points assessment (a) is based on the tipping point estimates of Armstrong McKay et al. (2022), while the past temperature range assessment (b) is based on palaeoclimate reconstructions (Marcott et al. 2013; Willeit et al. 2019; Kaufman et al. 2020; Fox-Kemper, Hewitt, Xiao, et al. 2021; Osman et al. 2021). Biosphere functioning (c) is based on assessments of substantially greater biosphere impacts at 2°C relative to 1.5°C (Schleussner et al. 2016; Hoegh-Guldberg, Jacob, Taylor, et al. 2018), growing risks to terrestrial & freshwater ecosystems (including to biodiversity, wildfires, tree mortality, carbon loss, and biome shifts) (Parmesan, Morecroft, Trisurat, et al. 2022), emerging climate-induced localised tipping points in the ocean (Heinze et al. 2021), and evidence for some natural carbon sinks (such as the Amazon or tundra) weakening and transitioning to net carbon sources beyond 1°C and 1.5°C respectively (Hubau et al. 2020; de Vrese et al. 2021; Gatti et al. 2021; Winkler et al. 2021). We also compare to (d) IPCC AR6's Reasons For Concern (RFCs), consisting of risks associated with unique & threatened systems (RFC1), extreme weather events (RFC2), uneven distribution of impacts (RFC3), global aggregate impacts (RFC4), and large-scale singular events such as tipping points (RFC5) (O'Neill, van Aalst, Zaiton Ibrahim, et al. 2022).

We follow the definition of Armstrong McKay et al. (2022) for tipping points, in being when change in a system (the 'tipping element') becomes self-perpetuating beyond a threshold and leads to substantial and widespread Earth system impacts. The resulting nonlinear system change is often abrupt and irreversible, but tipping points can sometimes be non-abrupt (in slow systems e.g. ice sheets) and reversible (if there is low hysteresis) on human timescales. Change beyond a tipping point is still difficult to reverse in either case, resulting in a significant future commitment to adverse impacts. Substantial and widespread Earth system impacts from climate tipping points include modifying the overall state of the whole Earth system, amplifying global warming, exposing large populations of humans to significant harm, and severely damaging or losing a unique feature of the Earth system.

The likelihood of passing climate tipping points grows with temperature increase compared to pre-industrial levels, and there is already a chance that at the current global warming of ~1.1°C (Gulev, Thorne, et al. 2021) at least one localised threshold has been crossed (Amundsen embayment, West Antarctica (Joughin et al. 2014; Rignot et al. 2014)). Based on Armstrong McKay et al. (2022) we estimate that up to 1°C of global warming presents a low likelihood of passing climate tipping points, but with a number of tipping points - in particular in the cryosphere (Greenland and West Antarctic ice sheets) - becoming possible beyond 1°C and likely by 1.5°C (Figure S1). We define crossing a tipping point as 'possible' beyond the tipping element's minimum threshold and likely beyond its central estimate. Threshold-free feedbacks such as gradual permafrost thaw (rather than permafrost collapse) and land carbon sink weakening can also become substantial beyond 1.5°C (Table S1; Armstrong McKay et al. (2022)).

Additional tipping points become possible or likely in the 1.5-2°C Paris range and even more in the 2-3°C range, leading us to classify >1.5°C as high likelihood and >2°C a very high likelihood of passing climate tipping points respectively. Some of the changes such as ice sheet collapse would play out over very long timescales (>1000 years), but others for example coral reef dieback and ocean convection collapse would occur much faster (10s-100s years). As more tipping points are passed it also becomes more likely that they interact and potentially 'cascade', with the impacts of triggering one tipping point making others more likely (e.g. amplified Greenland Ice Sheet melt triggering Atlantic Meridional Overturning Circulation collapse) (Wunderling et al. 2022).

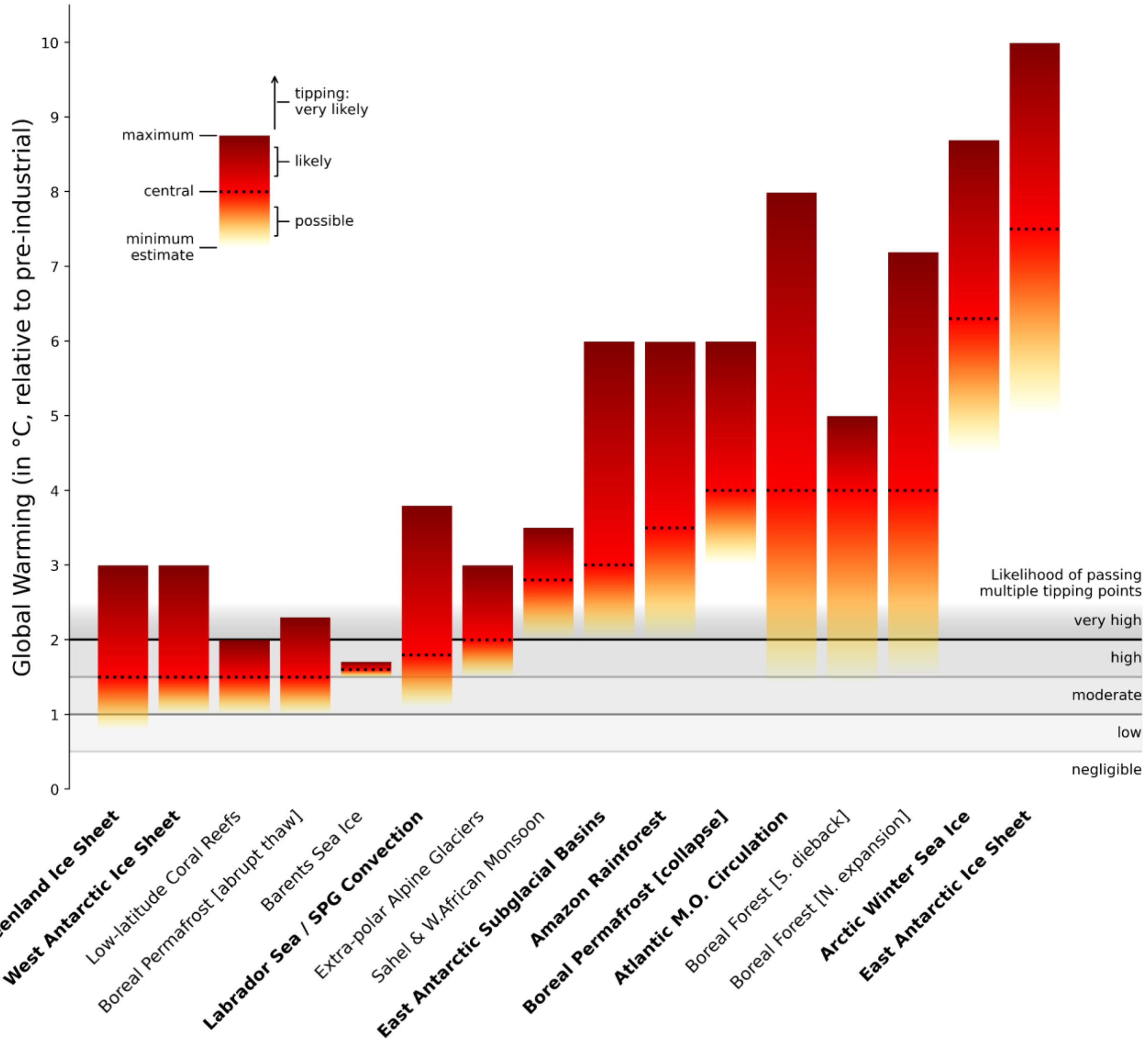

Figure S1. *Range of tipping threshold estimate (minimum, bottom of bar / yellow; central, dotted line / red; maximum, top of bar / dark red) for global 'core' (bold) and regional 'impact' climate tipping elements. The shading in the background indicates the likelihood of passing tipping points with rising warming levels, which increases from negligible (0°C) to very high (>=2°C) in accordance with Table S1. Based on: Armstrong McKay et al. (2022).*

These provisional limits closely reflect the IPCC's assessments of climate risk, with 1.5°C being supported as substantially safer for the biosphere than 2°C (e.g. with 6% of insect species ranges threatened at 1.5°C

versus 18% at 2°C (Hoegh-Guldberg, Jacob, Taylor, et al. 2018), ~7% of ecosystems shifting to new biome versus 13% (Hoegh-Guldberg, Jacob, Taylor, et al. 2018), and 0.44 vs. 0.51m sea level rise (Fox-Kemper, Hewitt, Xiao, et al. 2021). Even at present-day warming levels we are already committed to losing minor parts of some tipping elements prior to wider tipping points being reached, for example some outlet glaciers in West Antarctica (Joughin et al. 2014; Rignot et al. 2014) or in the southeastern Amazon rainforest (Gatti et al. 2021), supporting >1°C as being unsafe. Our low limit of 1°C and high limit of 2°C also reflects the original limits suggested by a report of the Stockholm Environment Institute (SEI) in 1990, which proposed 1°C and 2°C as potential climate 'targets' entailing lower and higher risk respectively (Rijsberman and Swart 1990). The SEI report stated that "temperature increases beyond 1.0°C may elicit rapid, unpredictable, and non-linear responses that could lead to extensive ecosystem damage", an assessment that closely matches our own 30 years later.

Analysis of committed future impacts from current change, due to the intrinsic inertia in many of the Earth system's processes and elements, also inform our boundary. The magnitude of the inertia varies among the subsystems, with the atmosphere having the weakest and the cryosphere the strongest. Systemic inertia means that there may be a time-lag between action and impact. Therefore today's pressure on the Earth system will commit the planet to impacts on the future on short to long time scales. We do not specifically analyse the possibility to temporarily transgress tipping points in slow systems like ice sheets (Ritchie et al. 2021), but remaining above their thresholds at ~2100 would likely ensure tipping. We analyse the committed change in two systems: the terrestrial biosphere and the cryosphere. We used simulations from the Parallel Ice Sheet Model (Bueler and Brown 2009; Winkelmann et al. 2011; Albrecht et al. 2020a, 2020b) for the Greenland and Antarctic ice sheets, and the Lund-Potsdam-Jena managed Land model LPJmL (Schaphoff et al. 2018) for global vegetation to compare the transient impacts to the time-lagged future changes which are already triggered for each decade under low- and high-emission scenarios RCP2.6 and RCP8.5. These idealised experiments allow us to assess the long-term impacts for a given transient warming level. We find that by 2100, while the projected sea-level rise from Greenland and Antarctica adds up to at most a decimeter, the equivalent of up to 11.3 metres global sea-level rise would be committed on the long-term under high-emission scenario RCP8.5. This long-term commitment would be limited to at most 3.5 metres if we were to restrict emissions following an RCP2.6 pathway. Global land carbon pools react highly heterogeneously with the potential to turn into carbon sources. Recent research suggests that the vegetation carbon pool has already begun to saturate (Duffy et al. 2021). We find that the global land can only remain a carbon sink by assuming a high CO_2-fertilisation rate of vegetation: by constraining the CO_2-fertilisation rate to the year 2020 in the LPJmL model, we find that the global land turns from a carbon sink to a source in the year 2034 (mean of LPJmL results under 4 ESM forcings) under RCP8.5 which corresponds to mean global warming levels around 2°C. Therefore, these less optimistic assumptions regarding CO_2-fertilisation exacerbate the findings of evidence line C in Table S1. These results serve as a stark reminder that we need to take the profound long-term and potentially irreversible impacts of short-term climate inaction into account when discussing climate policy measures. Every extra fraction of a degree of warming may commit us to long-term, potentially irreversible, changes and bring us closer to breaching tipping points in the climate system.

1.2 Biosphere

We assessed safe boundaries for two complementary measures of biodiversity to globally characterise the safe and just biosphere space for humanity. These are: the area of (dominantly) natural ecosystems and the functional integrity that secures ecosystem functioning. We use the most constraining ecosystem function to define the minimum viable value. We recognise important local variations may be possible, and that context is deeply important in understanding Nature's Contributions to People (NCP), however that there are overarching principles regarding the area, quality and distribution of nature that permit these rather

conservative estimates. We acknowledge that these two measures do not fully capture all facets of biodiversity, but rather are synthetic measures that address the full Earth surface and help support the different facets of biodiversity.

These measures aim to ensure interspecies justice to the extent possible in combination with Earth system stability (I1 justice).

1.2.1 Area of natural ecosystems

Area of natural ecosystems represents the area of largely intact natural ecosystems remaining with small human uses that do not significantly alter species composition and ecological functions, such as extensive grazing or low intensity use by indigenous communities or sustained harvesting of forest products. It includes pristine/untouched areas with high intactness, and may include protected areas, which are a protection (legal) rather than status category. Operationally, we determined 'natural ecosystem area' as defined by DeClerck et al. (2023) based on analysis of four spatial datasets describing conditions indicative for ecosystem intactness. This area refers to areas with both high ecological integrity and functional integrity. We emphasise that of the Earth system compartments, the biosphere is unique in its capacity to adapt to changing conditions. The biosphere is an important stock of nutrients including nitrogen, phosphorus, carbon, and water, and is a critical flow regulator of these elements. Changes in biosphere quality, notably through extensive habitat loss, fragmentation and degradation not only drive biodiversity loss, but alter its capacity to regulate carbon, water, and nutrient cycles within Holocene ranges.

In our approach to identifying the area of largely natural ecosystems required, we propose ESBs which both secure biosphere contribution to three major Earth system processes. climate regulation, hydrological flow regulation, and are necessary to halt the extinction of biodiversity (including the preservation of wild biodiversity). We estimated an area boundary of 50-60% of Earth's terrestrial surface from studies that assess the natural area and restoration needs to strongly reduce extinction risk (Strassburg et al. 2020) and maintain critical Earth system functions including high carbon storage and water regulation (Jung et al. 2021). At present, roughly 45-50% of the global land cover can be classified as dominantly natural (DeClerck et al. 2023), with large differences between ecoregions and ecosystems. Declining biodiversity and ecological functions indicate that, at least in many regions, this area is insufficient to maintain biodiversity, suggesting that restoration action to increase the area of nature is needed. Estimates of largely intact natural ecosystems needed to secure Earth system functions are widely varying depending on the targets set and indicators used for biodiversity and ecological functions, with Woodley et al. (2019) defining a range from 30% to 80%. However, a recent study determining restoration priorities identified that 60% of species extinction could be avoided and significant contributions to carbon sequestration could be made by restoring 15% of converted lands to nature (Strassburg et al. 2020), accumulating to a global natural land area of about 55% of the terrestrial area. While higher than the estimate of Allan et al. (2022) that indicated a 44% of land area requiring conservation action, our estimate includes all largely intact natural areas and not only those requiring conservation attention given current pressures. Our proposed boundary value is consistent with varied sources that prescribe approximately 15% area restoration over the current natural area of 45-50% (Newbold et al. 2016; Dinerstein et al. 2017; Willett et al. 2019; Riggio et al. 2020; Leadley et al. 2022). This is also consistent with the newly adopted restoration target (Target 2) of the Kunming-Montreal Global Biodiversity Framework (GBF) which translates to restoration of up to 10-15% of global land area (though this is expressed as restoring 30% of degraded lands, which comprise up to 40% of global land (UNCCD 2022), i.e. 10-15% overall). This comparison is an observation; we do not use the CBD target as part of the evidence for this boundary.

The minimum safe area for maintaining biodiversity and ecosystem functions cannot be stated by an exact number, as it depends strongly on the distribution of this area across ecoregions and ecosystems. At the same time, it also depends on the requirements in terms of ecosystem functions, e.g. the needs for carbon sequestration to offset emissions and required regulation of water resources in an uncertain climate change context. Given these uncertainties, the area boundary we propose is a range that uses the current area of natural land cover as a minimum, with the minimum (least stringent) boundary under the assumption of optimal allocation while a higher (more stringent) boundary represents a less optimal spatial allocation. Retaining largely intact natural lands provides a proxy for the different facets of biodiversity that should be considered in Global Biodiversity Framework targets (Díaz et al. 2020) at ecosystems, species and genes level and a number of globally operating NCP.

With the current natural ecosystem area at 45-50% (Table 1), restoration within the range 50-60% may be feasible. Agriculture is the largest contributor to loss of natural ecosystem area, and feeding a global population a healthy diet can be achieved without further land expansion (Willett et al. 2019); largely intact natural areas can also be compatible with human use and occupation. Thus access to sufficient food can be negotiated, managed and avoided through practices and policies that increase sustainable production, transition to healthy consumption, reduce loss and waste, and improve fair/just distribution.

Below we focus on the implications of this boundary for biodiversity habitat, carbon/climate and water. We note that the area of largely natural ecosystems replaces the “land boundary” in the planetary boundaries concept, which implied “largely intact lands” or areas of high biodiversity intactness (Steffen et al. 2015).

1.2.1.1 Biodiversity habitat
Extinction rate has a complex and non-linear relationship with habitat loss. Both theoretical arguments and empirical data support monotonically increasing per-species extinction probability with decreasing area. This relationship, known as the species–area relationship (SAR), is widely used in conservation science to predict the number of species likely to go extinct (Matthews et al. 2014), at both local and global scales, with habitat reduction (Ney-Nifle and Mangel 2000; Ulrich and Buszko 2003; Triantis et al. 2010, 2012; Fattorini and Borges 2012; Matthews et al. 2016; Chase et al. 2019). Although these relationships have been criticised for various reasons (Budiansky 1994; Connor and McCoy 2001; He and Hubbell 2011; Halley et al. 2013; Hanski et al. 2013), SAR provides - as a minimum at the worst - a valuable point of reference for the threat that habitat loss poses to biodiversity (Hanski et al. 2013). These relationships are useful in exploring the existence of critical thresholds in species loss. The existence of such thresholds is of conservation concern, because small additional losses of habitat below the critical threshold level may lead to rapid extinction and potentially irreversible regime shifts (Pardini et al. 2010; Swift and Hannon 2010; Pflüger et al. 2019).

For species population density and abundance, previous studies suggest that a critical threshold exists between habitat loss and extinction rates (Pflüger et al. 2019). This nonlinear erosion of diversity in response to increasing habitat loss is sometimes called an ‘extinction threshold’ (With and King 1999; Ovaskainen and Hanski 2003), and has been shown to occur when the amount of remaining habitat drops below 10-50% within a landscape (Andrén 1994; Swift and Hannon 2010; Banks-Leite et al. 2014; Dinerstein et al. 2017). While it would be appealing to be able to derive one universal threshold value, empirical studies have found mixed and species-dependent evidence for the occurrence of such a threshold (Swift and Hannon 2010). Threshold values for species diversity in mammals and birds ranged between 10 and 30% of native habitat cover (Andrén 1994), at around 20-30% protected area for terrestrial ecoregions (Gonçalves-Souza et al. 2020), for Amazonian mammals' and birds’ species richness between 19 and 43% (Ochoa-Quintero et al. 2015), while for richness in Australian forest-dependent bird species the threshold

has been estimated at 10% of habitat (Radford et al., 2005). Other studies have shown a threshold in the non linear erosion of abundance at around 30%, and for allelic richness at around 22% (Pflüger et al. 2019), at 29% when fragmentation and movement habitat cells is considered (With & King, 1999), and with further studies indicating that approximately 30% of native habitat is needed to preserve the integrity of vertebrate communities within each landscape (Banks-Leite et al. 2014; Pan 2016). Globally, it has been suggested that retaining half of ecosystem area intact would enable the conservation of 80% of species (Wilson 2016; Dinerstein et al. 2019).

Habitat loss and critical thresholds review relationships have been suggested for a variety of taxa, landscape types, and spatial scales. These large scale studies show variable responses and are too limited in number to nail down whether a critical threshold exists and the exact value. However, where thresholds were apparent, most occurred within the 10 – 30% range (see values cited above and Table 3 of (Swift and Hannon 2010). Furthermore, research shows that habitat specialists and generalists differ in their susceptibility to habitat loss and that values derived from SAR studies using total richness may be underestimating the impact of habitat loss on specialist species (Matthews et al. 2014). This - along with the empirical evidence that suggests that SARs underestimate species extinction by habitat loss and fragmentation (Fattorini and Borges 2012; Rybicki and Hanski 2013) - dictates that we should set our threshold at the upper range. Therefore, we set our extinction threshold at 30% intact habitat as defining critical and, we emphasise, permanent loss. Important for halting extinction losses or maintaining biodiversity is spreading conservation efforts across global ecoregions.

1.2.1.2 Carbon

Photosynthesis remains one of the primary means by which carbon from the atmosphere is taken up by plant respiration for long term storage in either biomass or in soils. Inversely, land-use change drives the combustion and loss of this stored carbon accounting for approximately 23% of CO_2-e emissions per year. Sequestration potential, and conversion risks are not equal however, with forest biomes, and peatlands having particularly high carbon storage. This storage and buffering potential is one key basis of the land boundary quantification in the planetary boundaries framework (Steffen et al. 2015) which concludes that on average (across all forest biomes) 75% of global forests must be kept intact to enable stable functioning of the Earth system. This same research concludes that retaining 85% natural ecosystem area is needed for boreal and tropical forest biomes, with 50% required for temperate forest biomes. The major forest biomes play an important role in land surface-climate coupling than other biomes because of their high productivity and carbon storage potential combined with their very large geographic extents. For these biomes, the amount of intact area needed may be more critically constrained by climate impacts than by halting extinction losses.

1.2.1.3 Water

Land use conversion leading to ecosystem change is a major driver of altered water flows, which in turn further undermines species composition, structure, and water flow partitioning, i.e., a vicious cycle of water-ecosystem decline. Planetary boundary science has quantified maximum water withdrawals (from aquatic ecosystems) and consumptive water use, as well as tolerable nutrient loading for Earth system resilience (Rockström et al. 2009; Steffen et al. 2015). The EAT-Lancet Commission, quantified the share of the planetary boundaries attributable to the global food system (Willett et al. 2019). These planetary boundary assessments of safe freshwater withdrawals and consumptive use, are both interesting and critically important in that they are established based on impacts on riverine and coastal aquatic biodiversity. However, they do not address how changes in biodiversity alter hydrological flow regulation by nearly intact nature, and the change in flow regulation that occurs with land use change. A green water boundary has recently been proposed (Wang-Erlandsson et al. 2022). Green water stocks and flows have been

significantly perturbed by human action including land use change. Wang-Erlandsson et al. (2022) have proposed that the green water planetary boundary can be represented by the percentage of ice-free land area on which root-zone soil moisture deviates from Holocene variability for any month of the year; they further argue that this boundary has been transgressed. From a biodiversity and Earth system function perspective, evapotranspiration (green water flow) is a key control variable because it responds to human impacts on ecological dynamics even though it only indirectly controls hydroecological Earth system functions. That is, in terms of hydroecological control, evapotranspiration, to a significant extent determined by canopy cover and biomass growth represents responses to ecological change (Wang et al. 2014; Wang-Erlandsson et al. 2022).

Evapotranspiration (ET), after precipitation, is the second largest flow of the global water cycle over land. Studies have demonstrated that land use conversion, from intact system to cropland and rangelands has decreased evapotranspiration by 5.6% globally, and increased run-off by 6.8% (Sterling et al. 2013). Land-cover change reduces annual total ET by approximately 3,500 km^3/yr (5.6%) with the largest losses in evapotranspiration associated with conversion of wetlands, whereas the greatest increase are associated with the creation of reservoirs (Sterling et al. 2013). Globally, CO_2 fertilisation and nitrogen deposition effects appear to have strong impacts on changing ET, however, the effect of land use change on land ET was also important locally (Mao et al. 2015). The consequences of loss of intact nature on alteration of hydrological flows remains poorly understood, although some studies suggest important impacts on regional weather patterns. Comparisons of biome scale change in evapotranspiration have not yet been conducted, although we hypothesise evapotranspiration will follow patterns similar to net primary productivity and carbon storage with the biggest losses in evapotranspiration potential in those biomes where net primary productivity is high. This same hypothesis was part of the rationale for emphasising the tropical forest land boundary in the planetary boundaries framework (Steffen et al. 2015). We recommend further research to identify biome scale evapotranspiration boundaries with evidence that biome scale deviations impact regional and global alterations of the water cycle.

1.2.2 Functional integrity

We define functional integrity as the capacity of habitats to maintain and provide the species interactions that secure ecosystem functions required to generate nature's contributions to people (NCP), or ecosystem services. Intact ecosystems have this capacity, however this is not generally the case in highly transformed human-dominated landscapes where a minimum level of functional integrity is needed to provide NCP, but often missing. The amount, quality of and distance to natural habitat in these landscapes can serve as a proxy for functional integrity. Functional integrity of human-dominated lands complements assessments of the required natural area by recognizing that non-intact lands, while highly modified, can provide critical contributions to people including NCP not related to native species composition (e.g., flood control, soil erosion reduction, water quality regulation) and those related to specific species such as insects (pollination and pest control), fungi (decomposition and soil carbon sequestration), mammals and birds (pollination, bushmeat, and pest control) amongst others.

Our measure of functional integrity uses the percent of natural or semi-natural habitat per unit area. We propose 1 km^2 as an appropriate spatial extent considering that many NCP are provided by species with little or limited mobility, and their benefits therefore mostly occur at the sub-kilometer scale (Steffan-Dewenter and Tscharntke 1999; Steffan-Dewenter et al. 2002; Klein et al. 2003b, 2004; Thies et al. 2003; Ricketts 2004; Kremen et al. 2007; Avelino et al. 2012; Fremier et al. 2013; Martínez-Salinas et al. 2016; Sirami et al. 2019). While there is significant context specificity in how biodiversity provides NCP (Tscharntke et al. 2016; Karp et al. 2018), the loss and decline of NCP with increasing distance from habitat is consistently demonstrated. For example, the number of species, and abundance of species able to provide pollination or pest control services rapidly decreases with the decrease in the amount of habitat available (Kremen et al. 2004, 2007, 2012; Ricketts et al. 2008; Chaplin-Kramer et al. 2011; Tscharntke et al. 2012; Kremen 2015; Kremen and Merenlender 2018; Garibaldi et al. 2020) and with increasing distance from source habitat (Denys and Tscharntke 2002; Steffan-Dewenter et al. 2002; Klein et al. 2003a, 2004; Kremen et al. 2004, 2007; Ricketts 2004; Ricketts et al. 2008; Avelino et al. 2012; Tscharntke et al. 2012; Martínez-Salinas et al. 2016). Particularly in human-dominated landscapes, NCP provisioning is related to fine scale biodiversity mediated processes such as reduction of soil and sediment loss [1-10 m], pollination [10-1000 m], pest control [10-1000m]) (Fremier et al. 2013). Thus, while specific NCP provided by distinct ecological communities will remain deeply contextual (Tscharntke et al. 2016; Karp et al. 2018), and the subject of both interesting and important research, a more universal hypothesis is that below a threshold value of functional integrity NCP are not provided.

Combining both amount of and distance from habitat measures, we derived a density threshold in habitat per km^2 as a proxy measure of functional integrity ESB. Rather than attempt to identify the correlation between these measures and the amount of NCP provided, which has not been successful in past studies, likely due to the diversity of species specific response options, we sought to identify the value below which NCP are no longer provided. We make the case that this value can serve as a critical safe and just threshold value. Current estimates have proposed that the boundary is laying between at least 10-20% semi(natural) habitat per 1 km^2 in human-dominated landscapes (Willett et al. 2019; Garibaldi et al. 2020; Tscharntke et al. 2021). To find broader and further support for these values we conducted a systematic analysis of 102 synthetic and original papers (representing 2215 studies in total) identified in 'Web of Science' (Clarivate Analytics, Philadelphia, USA) using two to three key words related to each NCP (Table S2).

We sought publications between 2010 and 2021 that contain values of habitat amount within 1 square km at landscape scale to synthesise the minimum amount of semi(natural) habitat needed to secure six critical local NCP including pollination, pest and disease control, water quality regulation, soil protection, natural hazards mitigation and recreation. Additional relevant papers were added either through expert consultation

or by a complementary search of the search terms in google scholar. The primary search survey produced 402 potentially eligible reviews and relevant papers based on titles and abstracts. Of those studies we conducted a full text review to confirm eligibility which was established if the reference contained measures of habitat quantity within 1 km at the landscape scale with measures appearing either directly in the source text, tables, supplementary information or in figures. A total 102 studies were considered eligible following this evaluation including 48 reviews and 54 original articles (Table S2).

Based on this systematic analysis, we find a critical functional integrity threshold across the six NCP equaling >20-25% of (semi-)natural habitat per square 1 km^2 ranging from 10 to 50% depending on the context and the NCP in question (Table S2). Mohamed et al. (2022 [pre-print]) present a more extended version of this analysis that also discusses the context dependency, and an analysis of the mechanisms of each NCP provisioning, quality of habitat, distance thresholds, and spatial configurations.

Table S2. *Boundary estimates for six major local NCP.*

**NCP (search terms)**	**Functional groups**	**Min. habitat quantity %/km^2 (weighted mean)**	**Min. habitat quantity %/km^2 (range)**	**Min. habitat quantity %/km^2 (median)**	**number of papers (total)**	**Summary**
**Pollination (“pollinat*” AND “habitat” AND “landscape”)**	insects	21	10-50	20	11 articles and 8 reviews/ meta-analysis (172)	Extensive evidence supports the positive roles that pollinators play in food production. SNH with sufficient floral resources plays a key role in maintaining the process with the need of 20% of SNH within km2 emerging as a minimum threshold.
**Pest and disease control (“Biological control*” AND “habitat” AND “landscape”)**	insects, birds, arachnids	19	10-38	20	10 articles and 11 reviews/ meta-analysis (260)	The direct relationship between pests and diseases, and their control agents can be highly specific with a variability of responses across taxa, overall SNH of at least 20% hosting beneficial organisms secure the provisioning of the service with important positive inputs to food security
**Water quality regulation ("riparian buffer*" AND "width*")**	plants	6	1.2-15	5	12 articles and 19 reviews/ meta-analysis (1480)	High supportive evidence of the role of riparian vegetative buffers both side of streams in mitigating surface and sub-surface water non-point pollutants from upland agricultural fields and thus improving streams water quality

**Soil protection ("soil erosion*" AND "vegetation*" AND "landscape*")**	plants	44	30-63	50	9 articles and 7 reviews/ meta-analysis (251)	Most studies reports the more vegetation cover(avoiding bare soil), the better soil is protected against erosion and degradation with the need of at least 50% of cover to maintain the process
**Recreation (“physical AND psychological*” AND “wellbeing*” AND “nature*”)**	Plants, birds	25	19-30	25	10 articles and 3 reviews (50)	Studies have shown clearly that experiences and activities in nature provide multiple and diverse physical and psychological benefits in health and well-being with the need of at least 25% of vegetation cover or green spaces within km2 in urban ecosystems to maintain the service
**Natural hazards mitigation ("landslide*" AND "vegetation cover*")**	plants	50	na	na	2 articles (2)	SNH plays a key role in regulating hazardous and extreme events e.g.landslides,however, most studies (only 2 found in our analysis) do not propose the minimum quantity of nature to be maintained.

1.2.2.1 Current state of functional integrity

We calculated the current state of global functional integrity using satellite data classified by land-cover type. We used the ESA Worldcover 10 m resolution land cover map (https://esa-worldcover.org/en) to create a binary classification where land was classified as either natural lands (1) or human-dominated [landshuman-modified working lands] (0). We then calculated an integrity value for each pixel using a focal function to calculate the mean of the binary for a 500-meter radius (approximately 1km^2) around each pixel. We then aggregated this layer to determine the percentage of pixels that meet or exceed our ‘functional integrity threshold’ (20%) in human-dominated lands. All analyses were done using the native Google Earth Engine interface. For full details see Mohamend et al. (2022 [pre-print]).

1.3. Freshwater

The loss of freshwater biodiversity is a major aspect of the global biodiversity crisis and has serious ramifications for food security in developing regions. Inland fisheries support the protein needs of hundreds of millions of people and this is directly at risk due to pressures on freshwater biodiversity (McIntyre et al. 2016). Flow alteration of rivers and wetland systems is a primary cause of reductions in aquatic ecosystem health that lead to reduced water quality, habitat losses and subsequently biodiversity losses (Bunn and Arthington 2002; Tickner et al. 2020). Freshwater species have evolved highly specific life history strategies as well as behavioural and morphological adaptations to take advantage of the natural flow regime of the rivers in which they persist (Lytle and Poff 2004). In catchments across the planet, river flows have different timing, magnitude, frequency and duration, of key flow events and native biodiversity make use of these foraging, breeding and dispersal opportunities. Similarly, estuarine and coastal fisheries species representing 77% of global catch depend on freshwater flows for part of their life cycle (Broadley et al. 2022). However, water resource developments such as hydro-electric and water supply dams and groundwater pumping for irrigation can substantially disrupt the natural pattern of surface flow, as well as connectivity across catchments, resulting in decreases in the abundance and diversity of aquatic organisms (Bunn and Arthington 2002; Poff and Zimmerman 2010; Dang et al. 2016). In addition to direct impacts on native biodiversity, flow alteration has also been shown to favour invasive species, most often as a result of environmental homogenisation, which can lead to further biodiversity losses (Marchetti and Moyle 2001; Poff et al. 2007). With the interconnectedness of groundwater and surface water in supporting aquatic ecosystems and the ecosystem services they provide, we have devised a safe target for surface water flow alteration and groundwater use based on the concept of environmental flows.

The surface and groundwater flows that support freshwater ecosystem functioning and the ecosystem services that they provide are known as environmental flows (Arthington 2012). The Brisbane Declaration of 2018 defines these as; “Environmental flows describe the quantity, timing, and quality of freshwater flows and levels necessary to sustain aquatic ecosystems which, in turn, support human cultures, economies, sustainable livelihoods, and well-being” (Arthington et al. 2018). This definition builds on decades of conceptual development and research that has established the importance of the natural pattern of flows to support biodiversity, healthy aquatic ecosystems and the ecosystem services that they provide (Poff et al. 1997, 2010; Arthington et al. 2006). An important aspect of environmental flows is a recognition that we may not ever be able to return to a “natural flow regime” in a given location, due to centuries of hydrological change, nor will every society necessarily wish to return to such a state (Acreman 2016; Poff 2018). As such explicit evaluation of flow-ecology relationships and societal preferences is required when responding to safeguarding environmental flows in local settings (Acreman 2016). Nonetheless, when setting an ESB for blue water across the entire Earth system, we adopt presumptive standards to be applied in the absence of knowledge around local environmental flow needs.

There are many methods for defining environmental flow needs for a given basin (Tharme 2003), and they generally revolve around identifying flow-ecology relationships that describe how the ecosystem responses to changes in river flow (e.g. Wang et al. 2013; McClain et al. 2014; Turschwell et al. 2019). The knowledge from these relationships can then be applied to identify environmental flows: the key facets of the flow regime that need to be protected, restored or delivered, in managed rivers to achieve a desired ecological state (Poff et al. 2017). Holistic environmental flow methods seek to define the environmental flows required to support the entire ecosystem (Tharme 2003), and by extension the ecosystem services that it provides. Defining the environmental flows on the basis of these relationships can occur via a bottom-up approach, where key flow events are identified and aggregated to design a managed flow regime (e.g. King and Louw 1998) or a top-down approach, where a maximum allowable extent of flow alteration is identified for flow management (e.g. Brizga et al. 2022). Top-down methodologies tend to be the more robust approaches

leading to better ecological outcomes (Tharme 2003). More recent approaches focus on defining environmental flow requirements for multiple catchments within a single region (e.g. Poff et al. 2010), supported by evidence of the transferability of flow-ecology relationships within a given region (e.g. Chen and Olden 2018). In spite of these advances, most catchments around the world remain unassessed for their environmental flow needs (Liu et al. 2021).

Given the inherently local to regional-scale nature of flow-ecology relationships and subsequently defining environmental flow requirements, the derivation of global-scale environmental flow standards relies on general rules of thumb and tend to follow a top-down approach (e.g. Richter et al. 2012; Pastor et al. 2014). The Variable Monthly Flows Method, developed by Pastor et al. (2014), allocates 60%, 45% and 30% of flows to the environment during the low flow, intermediate and high flow seasons respectively. Richter et al. (2012) applied a more conservative threshold of allocating 80% monthly flows to the environment, no matter the season. This approach emphasises the importance of maintaining the natural flow regime to support aquatic ecosystems and the ecosystem services they provide. While locally defined environmental flow requirements based on flow-ecology modelling will generally always be preferable, we have developed the global ESB in this study based on a sub-global ESB of all rivers meeting the presumptive standard devised by Richter et al. (2012).

The empirical evidence supporting the use of the 20% flow alteration as an ESB, when locally derived environmental flow standards are not available, is grounded in the concepts of the natural flow regime (Poff et al. 1997). Decades of eco-hydrological research has shown that instream organisms, and aquatic ecosystem function more generally, have evolved under the conditions of the natural flow regime (Lytle and Poff 2004), which supports myriad ecosystem services on which humans have come to rely (Yeakley et al. 2016). Given the wide range of natural hydrological variation in rivers across the world, and the ecological responses to this, there is not one single consistent relationship between different flows and ecological responses (Poff and Zimmerman 2010; Bruckerhoff et al. 2019). Nonetheless, altering the natural flow regime substantially has been shown to negatively impact aquatic ecosystems (Webb et al. 2013).

Generally, flow-ecology research is not presented in standardised units, such as the percent alteration from unaltered monthly flows (Rosenfeld 2017). It is commonly expressed in terms of hydrological metrics such as the mean daily flow or the timing and duration of high/low flow events that are then converted into environmental flow requirements (Poff et al. 2010). Nonetheless many empirical and modelling studies have shown how alteration beyond 20% of the natural flow regime, impacts aquatic organisms, ecosystem function and ecosystem services while limiting flow alteration below this threshold, tends to reduce ecological impacts. For example, empirical studies have shown how streams with flow alterations greater than 20% of the natural flow regime had highly impacted biodiversity (Poff and Zimmerman 2010; Webb et al. 2013). In contrast, studies directly comparing streams with highly altered and unaltered flow regimes have shown that streams that meet the safe and just ESB have near natural water quality (Rosenfeld 2017) and biodiversity (Carlisle et al. 2010, 2012; Rolls and Arthington 2014). Similar findings have been found in fisheries modelling where predicted catch rates in a flow dependent fishery show substantial reductions with increasing flow alteration but minimal declines when flow alteration is kept within 20% of the natural flow regime (Broadley et al. 2020).

In addition to the effects of substantial flow alteration, there are additional impacts on aquatic ecosystems and ecosystem services associated with water resources infrastructure such as dams and weirs through the reduction in connectivity they cause (Grill et al. 2019). Both large and small dams, and the reservoirs they create, act as barriers to upstream and downstream spawning migrations of fish (Pelicice et al. 2014; Couto et al. 2021) as well as reducing terrestrial biodiversity in the flooded areas of the impoundment

(Benchimol and Peres 2015). The loss of fish migration due to the barrier effects of dams and reservoirs impacts crucial freshwater fisheries that support protein needs and provide food security to many millions of people (Dugan et al. 2010; Orr et al. 2012). In addition to affecting biodiversity, dams and weirs also trap sediment and nutrients which impacts ecosystem services downstream, including the replenishment of deltas that are at increasing risk of sinking with declining sediment delivery coupled with sea level rise (Syvitski et al. 2009; Dunn et al. 2019). For example, the proliferation of large dams and weirs throughout the Mekong River basin is associated with declines in sediment delivery to the Delta and coastal zone (Loisel et al. 2014; Tessler et al. 2018), and subsequently increased saline intrusion across the Delta affecting agricultural production and population centres (Van Binh et al. 2020). The multifaceted impacts on aquatic ecosystems and their ecosystem services that come from flow alteration and the proliferation of large dams highlight the need for strategic approaches to development that meet the safe and just ESBs for blue water.

We have expressed the blue water ESBs in two ways: (i) as a representation of the spatial extent of exceedance of surface flow alteration and groundwater decline (similar to the ESB for biosphere integrity); and (ii) as a global volumetric aggregation of surface flow alteration and groundwater recharge. The former emphasises the degree to which surface flows and groundwater resources have been altered in many parts of the world, that cannot be conveyed in terms of the global volumetric totals. The spatial representation of the ESB addresses concerns raised by Gleeson et al. (2020) about the limitation of the solely volumetric approach to previous derivations of the planetary boundary for water. By defining the safe ESBs based on the global extent of local-scale alteration to blue water flows, there is a direct connection between the global and sub-global boundaries which can facilitate local adoption of the ESB (sensu Zipper et al. 2020). The global scale volumetric aggregation provides a comparison to the global accounting of blue water flows and the quantities of the previous planetary boundaries, and enables comparisons to estimates of just access levels expressed in the same volumetric terms (e.g. Rammelt et al. 2022).

1.3.1 Surface water

The safe ESB is a spatial realisation of a sub-global alteration budget (20% monthly flow alterations, see main text) and to complement this we estimated a global volumetric aggregation of available alteration of flows under the ESB. We derived this global estimate from modelled pre-industrial and contemporary flows derived from the WBM water balance model runs (Wisser et al. 2010) at 6-minute grid cell resolution using the TerraClimate high resolution data set of monthly climate forcings (Abatzoglou et al. 2018) for the period 2000-2020. The modelled long-term mean contemporary global annual discharge of approximately 38,000 km^3 under this scenario was consistent with results from the literature, though at the low end of other studies (Table S3).

From a justice perspective, this approach emphasises interspecies justice more than Earth system stability as it is seen as more relevant for this domain.

Table S3. *Global annual continental runoff from comparable global modelling studies*

**Study**	**Time period**	**Estimate (km^3/yr)**	**Model/data source**
Zhang et al. (2018)	1984-2010	42,158*	Variable Infiltration Capacity land surface model
Trenberth et al. (2011)	1979–2000	40,000	NCAR CCSM4
Wisser et al. (2010)	1961-1990	37,401	Water Balance Model (WBM)
Sperna Weiland et al. (2010)	1958-2001	36,812	PCR-GLOBWB
Recknagel et al. (2021)	1901-2016	39,828	WaterGAP 2.2d
Rost et al. (2008)	1971-2000	36,921	LPJmL
Hanasaki et al. (2010)	1985-1999	41,820	H08
This study	2000-2020	38,153	Water Balance Model (WBM)

* calculated based on estimated global runoff of 318 mm/yr over land surface excluding Antarctica and Greenland

1.3.1.1 The safe ESB for surface water and previous Planetary Boundaries

As noted above, the safe ESB for surface water builds on the previously developed PB for water, with modification to accommodate the spatial importance of the sub-global boundaries for Earth system domains like water. Nonetheless, the globally aggregated volumetric number of the safe ESB can be compared with previous iterations of the PB for water, which followed a volumetric approach. The sub-global safe ESB is stricter in conceptual and quantitative terms than previously quantified PBs for surface water, though the globally aggregated volumetric number is larger. The difference between the safe ESB for surface water

and the previously defined PB is twofold, including the region of the globe that is included in the analysis and the conceptual approach to the quantification.

When originally defined by Rockström et al. (2009) (and then updated by Gerten et al. (2013) and adopted by Streffen et al. (2015), the PB for surface water was based only on what was defined as “accessible water”, which had been previously defined by Postel (1996) as water that could be appropriated by humans. This approach excluded much of the water in the Amazon, the Congo and the high arctic as it was deemed *inaccessible*. Working from this approach, meant that the PB defined by Rockstrom et al. (2009) was based on the total accessible water of 12,500 km^3/year. In their update to the PB approach, Gerten et al (2013), included water stored in dams (approximately 7,000 km^3/year) to be included in their updated PB, which meant the PB was based on total accessible water of 16,300 km^3/year. The safe ESB for surface water developed in this paper is primarily a sub-global boundary given the nature of impacts on aquatic ecosystems, however, when aggregating across the world, we used all of the world’s annual runoff, approximately 38,000 km^3/year. As human development progresses and the climate continues to warm, more and more of the water in regions like the Amazon and Congo basins is becoming accessible and as the climate warms, land in the high arctic may become more accessible too. As such, these regions need to be included in the application and development of the safe and just ESBs.

Another quantitative difference between the previous PBs and the safe ESB for surface water is that Gerten et al. (2013) and Rockström et al. (2009), applied a different "withdrawal budget" depending on the time of year. It was considered safe to withdraw more water during the high flow season than the low flow season (between 30-60% of flows). The safe ESB sets the safe alteration to be 20% each month, to recognise the critical importance of high flows for aquatic ecosystems, particularly for things like access to floodplain habitat which often fuels fisheries (e.g. Leahy and Robins 2021) and provides access resources for fish to survive the dry season (e.g. O’Mara et al. 2021). This is particularly important in the many dry regions of the world where people and ecosystems rely on aquatic production during the wet season. The consequence of this different approach is a much higher globally aggregated number for the safe ESB but a stricter sub-global boundary. If we apply the safe ESB to the global runoff considered in Rockstrom et al. (2009) and Gerten et al. (2013), the aggregated number ESB would be 2,500 km^3/year or 3,260 km^3/year respectively, which is lower than the numbers presented in those papers.

The conceptual difference between the safe ESB for surface water and the PBs is based around the type of alterations to the flow regime that are considered safe. The approach of the PBs was to consider consumptive use, meaning it considers only water that is removed from the aquatic ecosystem. In contrast, the ESBs focus on flow alterations be they increased or decreased flows or consumptive or non-consumptive uses. This means that non-consumptive uses must ensure return flows reach the aquatic ecosystem within the same month to not risk exceeding the safe ESB. In addition, the safe ESB accounts for the negative ecological impact of artificially increased flows, often provided during the low flow season for anthropogenic use as such flows often create conditions more suitable for invasive species than native species.

1.3.2 Groundwater

In the absence of a consistent data source on baseline aquifer volumes, we used satellite derived estimated groundwater storage to derive the safe boundary for groundwater. We used the Gravity Recovery and Climate Experiment (GRACE) satellite data covering the period 2003-2016 (data files accessed at http://www2.csr.utexas.edu/grace/RL06_mascons.html) to estimate monthly changes in terrestrial water storage (TWS), being the sum of soil moisture, groundwater, surface water, snow water, and canopy storage:

$$TWS = SMS + GWS + SWE + SWS + CS$$

where TWS is changes in terrestrial water storage, SMS is the soil moisture storage change, GWS is the change in groundwater storage, SWE is the change in snow water equivalent, SWS is the change in surface water storage (i.e., inland surface and reservoir storage) and CS is the canopy storage change.

Groundwater storage anomalies (i.e., GWS) were estimated by rearranging the above equation. Soil moisture components of varying depths (e.g., 40-100 and 0-200 cm), canopy storage, and snow melt were removed from the GRACE-derived TWS time series (2003-2016). These data (SMS, SWE, and CS) were derived or acquired from the latest Global Land Data Assimilation (GLDAS) NOAA Land Surface Model L4 v2.1 (Beaudoing et al. 2020). The uncertainties associated with the relative contributions of these water storage components (SMS, SWE, and CS) obtained from model simulations to GRACE-derived GWS changes could be larger for some regions due to the presence of strong variability in surface reservoirs and snow/ice cap storage. As opposed to regional studies where the effects of surface water variations from lakes and reservoirs with strong changes can be reasonably managed and isolated from TWS by reconstructing these variations, using satellite-derived water level variations and a synthesised kernel functions (Ndehedehe et al. 2017; Agutu et al. 2019), handling similar effects on a global scale is more complicated and not feasible. To circumvent this, much of the regions with substantial inland surface water storage changes or strong gravimetric signals (e.g., Caspian Sea, Black Sea, Lake Victoria, and other significant water bodies) have been masked out. This decision also acknowledges the argument that the use of SWS as an auxiliary data from model reanalysis to aid the estimation of GWS from GRACE introduces more uncertainties into the process (Chen et al. 2016).

To further minimise uncertainties in the estimated GWS from GRACE TWS, residual ice/snow cover from areas (e.g., Patagonia, Alaska, Himalayas, Swiss Alps, etc.) with large variations were also masked using the world distribution of glaciers and ice caps extents. This was warranted because of the higher uncertainties in the simulations of these quantities by the GLDAS model. Further, given that some glaciers are small, a buffer zone of 1 arc degree was created to help capture and remove such glaciers. The water budget approach employed in isolating the residual time series representing changes in groundwater is consistent with a widely used approach in GRACE-derived groundwater storage studies (e.g. Ojha et al. 2019; Thomas and Famiglietti 2019). TWS variations were based on the Center for Space Research RL 06 version 02 GRACE mass concentrations (mascon) product obtained from NASA. These GRACE observations are preferred to other GRACE solutions based on spherical harmonics since it exhibits less signal leakage and a posteriori filtering is also unnecessary because this GRACE mascon solution relies on geophysical constraints to suppress noise in the data (e.g. Watkins et al. 2015).

Using the spatial time series derived from this analysis, we estimated the annual recharge volume for each pixel in the GRACE record by quantifying the difference between the deepest groundwater observation in a given year and the shallowest observation the following year. With a spatial time series of recharge depths, we then calculated the global average depth in mm, which we converted to an estimated volume by multiplying by the total land area containing GRACE observed quantities or pixels. Due to the time period of the GRACE satellite data, our estimated global annual groundwater recharge includes the signal from existing climate change. The impact resulting from this change has been a major contributor to intensive human water withdrawal or groundwater depletion in several global hydrological hotspots. Nonetheless, it is broadly consistent with other published studies based on global hydrological models and empirical observation and modelling that cover late 20th century and pre-industrial time periods (Table S4).

Table S4. *Global annual recharge values from recent published studies*

Study	Time period	Estimate (mm/yr)	Estimate (km^3/yr)	Model/data source
Döll and Fiedler (2008)	1961-2000	N/A	12,666	WaterGAP
Wada et al. (2010)	1958-2000	N/A	15,200	PCR-GLOBWB
Mohan et al. (2018)	1981-2014	134	13,600	Linear regression
Herbert and Döll (2019)	1981-2010	N/A	16,480	WaterGAP 2.2b
Moeck et al. (2020)	1968-2018 (approx)	234	N/A	Observed data*
Reinecke et al. (2021)	1661-1860	140	18,255*	Eight global hydrological models
This study	2002-2016	122	15,753	Remote sensing (GRACE)

* Reinecke et al. (2021) provided their estimate of average annual recharge in mm, which we converted to km^3

1.4 Nutrient cycles

In this section we detail the derivation of our proposed safe and just (NSH) ESBs for Nitrogen and Phosphorus. In our Earth system justice analysis (see Section 2), these ESBs meet most of the I1, I2 and I3 criteria.

1.4.1 Nitrogen

Nitrogen (N) is a key macronutrient required for agriculture, for which the main sources have historically been nitrogen-fixing plants and lightning with internal transfer via manure, and more recently synthetic fertilisers and atmospheric deposition. However, in many regions much more N is applied to croplands and pastures than is required, with on average only ~46%(40–53%) of applied N taken up by harvested crops (the nitrogen use efficiency, NUE) (Zhang et al. 2021b). The rest is termed N surplus and can enter the atmosphere, terrestrial ecosystems, surface water, and groundwater as N losses in various forms including ammonia and nitrous oxide to air and nitrate to water (Figure S2). N losses can trigger freshwater eutrophication or drinking water contamination by nitrate (NO_3) and degradation of terrestrial ecosystems adapted to low nutrient availability by ammonia (NH_3) and nitrous oxides (NO_X) when their concentrations are above critical limits (de Vries et al. 2021; Schulte-Uebbing and de Vries 2021). Upon reaching the ocean, excess nutrient input from land (including both N & P) have led to an increase in the number of known hypoxic coastal sites (oxygen concentrations ≤2mg/L) by more than 9 times since 1950, and open ocean oxygen minimum zones expanding by ~4.5M km^2 over the past 50 years with complex impacts on fisheries (Breitburg et al. 2018). N_2O emissions also contribute to climate change (with 273 times the global warming potential of CO_2 over 100 years; (Forster, Storelvmo, et al. 2021)), but this effect does not affect setting of the climate ESB, which is specified using temperature change rather than concentrations of greenhouse gases. Our ambition is that inter-boundary interactions will be analysed more explicitly in future iterations. For example, in addition to N_2O emissions increasing radiative forcing, N cycle disruption might also lead to a net-cooling effect via increased carbon sequestration (Erisman et al. 2011; De Vries et al. 2017).

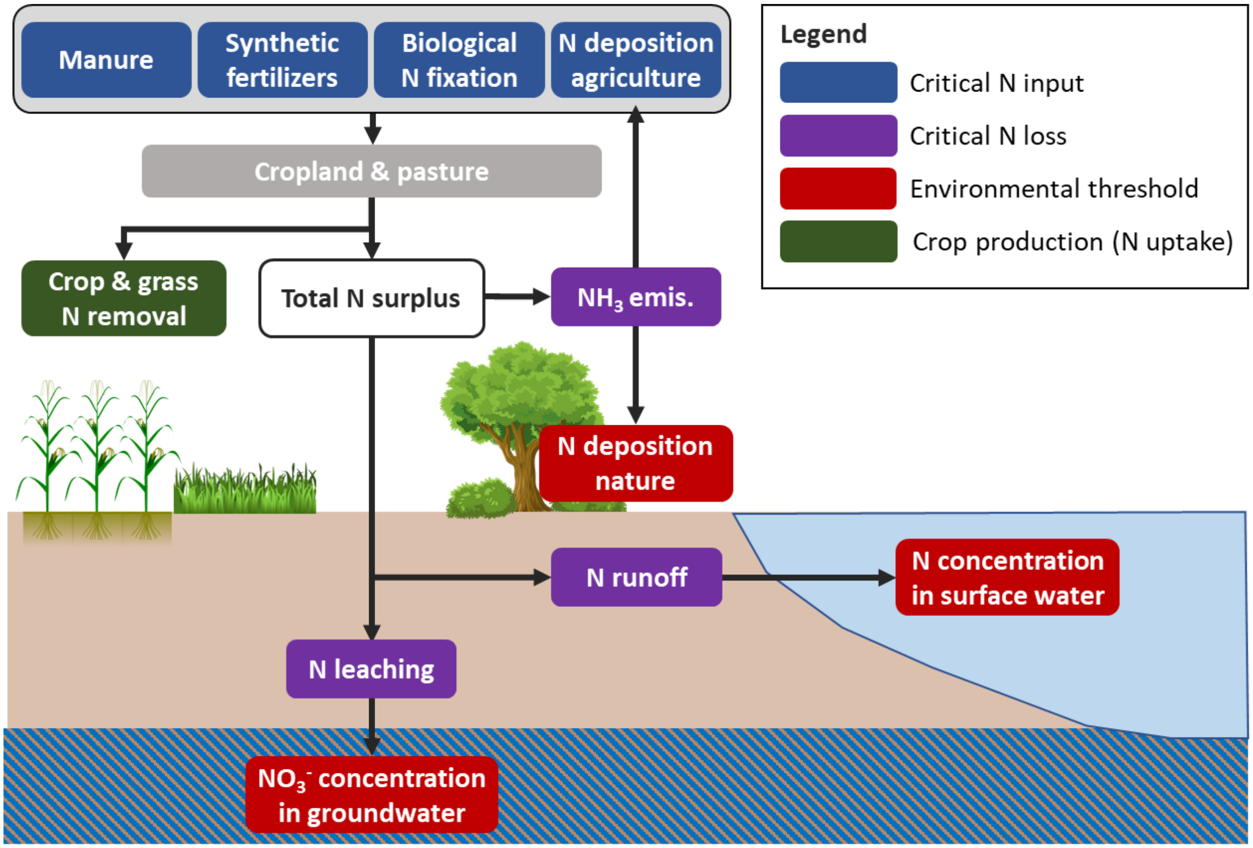

Figure S2. *Simplified representation of relationships between critical N inputs, critical N losses, environmental thresholds, and crop/grass production (crop/grass N uptake). Adapted from (Schulte-Uebbing and de Vries 2021).*

In 2009 the Planetary Boundaries framework first proposed a global safe boundary for N pollution beyond which unacceptable global environmental change occurs, setting the boundary for intentional new N fixation (i.e. anthropogenic synthetic fertiliser production and biological fixation by leguminous crops) as a first guess well below current rates (~140 TgN/yr) at 35 TgN/yr (Rockström et al. 2009) (Table S7). Several updated quantifications followed, with De Vries et al. (2013) revising the new fixation boundary with an improved methodology to 72 (62-82) TgN/yr with respect to protection of surface water, the lower bound being adopted in the Planetary Boundaries update in 2015 (Steffen et al. 2015). More recently the focus of N boundary-setting has shifted from new fixation and total inputs to N surplus and losses, on the basis that it is the surplus N leftover after harvest that directly determines the N available for loss to water or atmosphere rather than the input magnitude. Springmann et al. (2018) calculated a global surplus N boundary of 90 (67-146) TgN/yr for protection of surface water while Chang et al. (2021) calculate 106-123 TgN/yr, compared to the current value of ~134 TgN/yr.

Here we adopt values from the most recent and comprehensive analysis available for the current status and safe boundaries of agricultural N (Schulte-Uebbing et al. 2022). The global safe N limits are set to avoid significant pollution to surface water (from total N runoff), groundwater (from NO_3 leaching) and terrestrial ecosystems (from NH_3 and NO_X emission and re-deposition) across wide areas (see Figure S2). The IMAGE model was used to calculate critical N losses, surpluses, and inputs on agricultural land at a spatial resolution of 0.5x0.5 degrees based on local critical concentration limits for ecosystem or health disruption derived from the literature (N concentration on surface water of 2.5 mgN/L to prevent eutrophication, N

deposition on terrestrial ecosystems of 5-20 kgN/ha/yr depending on biome to prevent vegetation shifts, and the WHO drinking water limit of 50 $mgNO_3/L$ or 11.3 $mgNO_3$-N/L for groundwater to maintain drinkability) (Schulte-Uebbing et al. 2022). Critical N losses are a function of environmental characteristics such as water discharge, share of agricultural land in a region, or sensitivity of receiving ecosystems, whereas corresponding critical N surpluses are a function of N loss fractions, which are affected by soil type, climate, hydrology, slope, crop type, and agricultural management (see Beusen et al., 2015 for details on the IMAGE-GNM model). Finally corresponding critical N inputs were calculated as a function of N use efficiency. These subglobal critical N losses, surpluses, and inputs were then aggregated to calculate global ESBs (Table S5). To calculate a tolerance range for the ESBs we also recalculate each ESB for a range of critical concentrations based on the range of stricter and looser values used in the literature (1-4 mgN/L for surface water, c. +/-25% for terrestrial ecosystem deposition per biome [see Table S5 in Bobbink et al., (2010)], and 10-11.3 $mgNO_3$-N/L for groundwater using the lower US standard as the lower bound (Ward et al. 2018). A No Significant Harm (NSH) boundary can be set for drinking water quality from nitrate leaching via a similar approach (see below).

Table S5. *Global totals of current values and safe limits (TgN/yr) for total input, new fixation, surplus, and losses for N in relation to the protection of surface water (from N runoff), terrestrial ecosystems (from ammonia emissions), and groundwater drinking quality (from N leaching), and in combination. Bold numbers indicate headline indicators. Values are from Schulte-Uebbing et al. (2022) except for Combined (SW, & TE) which were recalculated for this study following the same methodology.*

**N indicator / Environmental threshold targets**		**Current value**	**Safe limit**	**Lower safe bound**	**Upper safe bound**
Total Input	Surface Water (SW)		198	117	247
	Terr. Ecosystems (TE)		220	165	262
	Groundwater (GW)	232	245	235	245
	Combined SW & TE		143	87	189
	Combined SW, TE, & GW		134	85	170
New Fixation	Surface Water		92	58	115
	Terr. Ecosystems		106	83	125
	Groundwater	122	107	102	107
	Combined SW & TE		70	48	90
	Combined SW, TE, & GW		65	47	79
**Surplus**	Surface Water		92	49	120
	Terr. Ecosystems		101	74	123
	Groundwater	**119**	117	111	117
	**Combined SW & TE *[safe]***		**61**	**35**	**84**
	**Combined SW, TE, & GW *[safe & just]***		**57**	**34**	**74**
Losses	Surface Water	23	21	-	-
	Terr. Ecosystems	32	29		
	Groundwater	37	34		

These proposed thresholds align with some of existing thresholds (Table S7), but are more advanced in the following aspects:

1. In contrast to a focus on total global input (in particular fixed N) by many Planetary Boundary (PB) studies, these proposed thresholds follow recent work (e.g. Springmann et al. 2018; Chang et al. 2021; Schulte-Uebbing et al. 2022) in focusing on N surplus and N losses (Figure S2) as these are what have the direct impact on ecosystems and are not sensitive to assumptions on nitrogen use efficiency (NUE) which are affected by management. Surpluses and losses can thus be (partly) decoupled from inputs by improving NUE, indicating a major potential lever of transformation.
2. By considering the N thresholds on a finer spatial scale, the approach allows additional necessary N inputs in under-fertilised regions, where increased usage of N does not exceed critical concentration limits, while it enhances crop yield. This acts to increase the potential global safe N limit. In contrast, taking account of all multiple environmental thresholds instead acts to reduce the global safe limit lower than the individual limits for each.
3. Working with the N thresholds on a finer spatial scale also recognized the impacts of N pollution, which occur on a local/regional scale and have large spatial variation. The global aggregated safe limits presented above are useful but have their limitations, as N pollution is mostly a localised issue that does not globally aggregate in the way that for example CO_2 pollution does. It is a global value assuming optimal regional allocation.

Taking account of both N redistribution and the spatial heterogeneity of N pollution impacts allows for a more nuanced approach than the original single global input cap proposed by the planetary boundaries approach.

Beyond ecological damage, N pollution can cause harm to humans in several different ways (de Vries 2021). Contamination of water by N (in particular by nitrate (NO^-_3) and nitrite (NO^-_2)) can render it harmful to human health (WHO 2017; Ward et al. 2018), although in surface water eutrophication (and its harmful consequences, such as fishery collapse or toxin release) is likely to occur first. Air pollution by N compounds is also a serious problem. Recently, it was estimated that NO_2 pollution causes 4.0 (1.8–5.2) million new paediatric asthma cases annually, being equal to 13 (6–16) % of the global asthma incidence (Achakulwisut et al. 2019). Particulate matter ($PM_{2.5}$) linked to agriculture, in particular ammonia as a $PM_{2.5}$ precursor from intensive animal agriculture and artificial fertiliser use, also has significant human health consequences, with agriculture the primary source of $PM_{2.5}$ in Europe, parts of East Asia, and Northeastern USA and responsible for around ~20% of the ~3.3 million/year deaths from $PM_{2.5}$ (Lelieveld et al. 2015). A 50-100% reduction in agricultural emissions is projected to be able to prevent global air pollution mortality by ~8-25% (~250,000-800,000 people/yr) (Pozzer et al. 2017), and nitrogen accounting for 39% of global $PM_{2.5}$ exposure in 2013 potentially caused 23 million years of life lost (Gu et al. 2021). However, there is currently insufficient work quantifying a limit for N-related air pollution with respect to human health at a global scale, although it is likely that NH_3 emission levels ceilings that protect nature also protect human health from NH_3-induced $PM_{2.5}$ effects (Schulte-Uebbing et al. 2022). PM formation from agricultural NH_3 emissions strongly depend on chemical and meteorological conditions varying in time and in space, with for example aerosol (PM) formation in Europe and North America generally not limited by NH_3 availability and a reduction in NH_3 emissions in these regions not linearly translating into a reduction in PM formation. Assessing the effects of NH_3 on PM formation would therefore require detailed atmospheric chemistry models that capture these processes, which has not yet occurred on a global scale. As a result, in this iteration we only include harm from groundwater contamination and ecological impacts for the global harm quantification, but in future work harm for air pollution should also be directly incorporated.

For the boundary of harm to health resulting from N contamination of water, we use the WHO drinking water quality threshold of 50 $mgNO_3/L$ (i.e. 11.3 $mgNO_3$-N/L) set to prevent infant methemoglobinemia (Ward et al. 2018). For groundwater leaching this results in global limits of 245 TgN/yr for total input, 107 TgN/yr for

new fixation, 117 TgN/yr for surplus, and 34 TgN/yr for losses (Table S5). This concentration threshold for NSH is well above the 2.5 mgN/L critical concentration used for safe limit setting with respect to surface water eutrophication, meaning that for surface water the safe limit (for nature) is far below the harm boundary (for human health) for N contamination. Above the safe limit there is an increasing risk for eutrophication (also depending on the phosphorus concentration), leading in extreme cases to harmful consequences from algal blooms releasing other toxic compounds, fish stocks collapsing, or loss of cultural ecosystem services such as recreation. Additionally, parts of the population (e.g. infants, those with less access to water filtration, etc.) are more vulnerable to drinking water contamination by fertilisers than others (e.g. in India, where nutrient pollution is particularly high; (Brainerd and Menon 2014)), and as the WHO guideline is set for acute exposure a safe limit for chronic exposure would likely be lower (Ward et al. 2018).

Based on this, we take a drinking water quality threshold of 50 mgNO_3/L (11.3 mgNO_3-N/L) as a boundary for groundwater NSH at the subglobal level and the previously quantified safe limit for groundwater pollution (117 TgN/yr surplus, corresponding to ~245 TgN/yr total input at current NUE) at the global level. However, as discussed above harm also occurs beyond the more stringent safe ESB (2.5 mgN/L and 5-20 kgN ha/yr for subglobal, 61 TgN/yr surplus corresponding to ~143 TgN/yr total input at current NUE for global) due to the loss of ecosystem services. However, in some regions local circumstances result in the subglobal groundwater boundary being reached before the subglobal surface water and terrestrial ecosystem boundaries that determine the safe ESB (Schulte-Uebbing et al. 2022). Aggregating the lowest of these three boundaries across each subglobal area leads to a more stringent global combined safe and just boundary (57 TgN/yr surplus, corresponding to ~134 TgN/yr total input at current NUE for global) than both the global safe ESB (61 TgN/yr surplus) and groundwater harm boundary (117 TgN/yr surplus), as this avoids the safe-only ESB resulting in groundwater pollution in the regions where that happens sooner (Table 1). For the distribution of where exceeds the safe and just N boundary at a subglobal scale for Figure 3 in the main text, as spatial data is lacking for some of our subglobal variables (terrestrial deposition and groundwater drinkability) we use a map of current N surplus (estimated from the IMAGE model; (Beusen et al. 2015, 2022) relative to critical N surplus (Schulte-Uebbing et al. 2022) as a proxy for where excess N is likely to lead to environmental and social harm.

1.4.2 Phosphorus

Phosphorus (P) is another key macronutrient required for agriculture. The primary sources of P beyond natural weathering (~10 TgP/yr pre-industrial, not all to agricultural soils) are mined rock phosphate and manure (~17 TgP/yr each, the latter being recycled within the agricultural production system) (Table S6; Figure S3). As with N, more P is applied to croplands and pastures globally than is taken up, with about ~60-66% of inputs taken up by harvested crops (i.e. the phosphorus use efficiency, PUE) (Zou et al. 2022). Unlike N, surplus P mainly accumulates in soil by sorbing to e.g. clay particles and aluminium and iron oxides, leading to a gradual buildup of P in agricultural soils and benefiting crop yield over the long term if P is below a level affecting crop yield (Li et al. 2011; Bai et al. 2013). Especially in P-depleted soils (such as heavily weathered tropical soils rich in iron or aluminium compounds) applied P tends to be adsorbed by the soil and is unavailable for crop growth, limiting the effectiveness of fertiliser application (Magnone et al. 2019) which reduces when the soil P status is increased by excess P input (so-called legacy P). However, this P can later be released to surface water via soil erosion (Alewell et al. 2020), along with P from sewage, manure, and other sources (e.g. industry and detergents) (see pathways to surface water box in Figure S3). P concentrations beyond a critical level in surface water are likely to trigger widespread eutrophication, while subsequent P adsorption and leaching by waterway sediments frustrates recovery from eutrophication (Carpenter 2005; Carpenter and Bennett 2011; Mekonnen and Hoekstra 2018; Springmann et al. 2018). As described in section 1.4.1, excess nutrient input from land (from both N & P) have also led to increasing areas of coastal hypoxia (Breitburg et al. 2018), and geological data suggest

that elevated P loss to the ocean maintained over 1000s of years could result in global ocean anoxia (Watson et al. 2017).

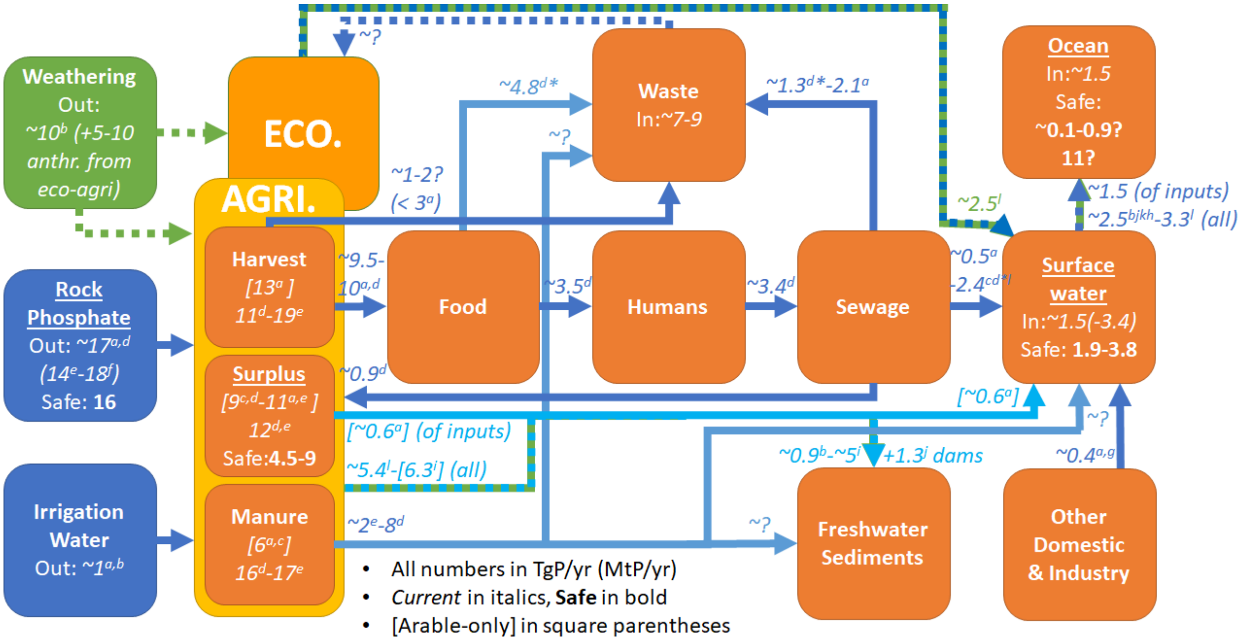

Figure S3. *Schematic showing flow of P through agro-ecosystems, showing flows (arrows - blue is anthropogenic, green is natural) to each pool (orange squares). Current values (italics, arable only in square parentheses) and proposed safe limits (bold) indicated on key flows (TgP/yr); underlined names indicate pools in Table S6. Citations for values are: a) Mekonnen & Hoekstra, (2018); b) Carpenter & Bennett, (2011); c) Zhang et al., (2021a); d) Cordell & White, (2014); e) Bouwman et al., (2013); f) Steffen et al., (2015); g) Van Drecht et al., (2009); h) Kemena et al., (2019); i) Alewell et al., (2020); j) Beusen et al., (2005); k) Seitzinger et al., (2010); l) Beusen et al., (2022).*

In 2009 the Planetary Boundaries framework first proposed a global safe boundary for P pollution, setting it for loss to the ocean just above current rates (~9 TgP/yr) at 11 TgP/yr with the aim of preventing global ocean anoxia developing within the next millennium (Rockström et al. 2009). Several updated quantifications followed, with Carpenter & Bennett (2011) estimating PBs for freshwater eutrophication due to P based on a range of quantifications of P flow from fertilisers to erodible soils, of which ~6.2 TgP/yr (vs. ~14TgP/yr estimated current rate) was adopted in the Planetary Boundaries update (Steffen et al. 2015) (Table S8). This approach implicitly assumes that soil erosion is the principal source of P to surface waters, neglecting internal P inputs by manure and human waste following animal and human consumption, and so could lead to an overestimate of the P boundary. Furthermore, the potential for improvements in food chain PUE and enhanced P recycling to increase the P boundary are not accounted for. More recently Springmann et al. (2018) described a model approach including those processes and they estimated a safe boundary for total P input to agricultural soils of 6-12 TgP/yr based on critical concentrations of 50-100 mgP/m^3, increasing to 8-16 TgP/yr with 50% P recycling. This further increases to 16 (8-17) TgP/yr if allowing a global rebalancing of P usage by also increasing P inputs in under-fertilised regions alongside decreasing P inputs in over-fertilised regions. Based on this approach, Zou et al., (2022) and Zhang et al. (2021a) calculated that this would be associated with a P surplus of 4.5-9.0 TgP/yr from croplands (vs. ~10 TgP/yr currently) and 1.9-3.8 TgP/yr input to freshwaters (vs. ~1.5 TgP/yr currently).

Here we adopt values from the most recent and comprehensive analysis available for the current status and safe boundaries of agricultural P from the analysis of Springmann et al. (2018) and Zhang et al. (2021a) (Table S6). The global safe P limits are set to avoid significant pollution to surface water from total P runoff (following Springmann et al. (2018) and Mekonnen & Hoekstra *(2018)* in focusing on losses derived from P application, rather than all potential losses including natural P) across wide areas, based on critical concentration limits of 50-100 mgP/m^3 for surface water to prevent eutrophication (Springmann et al. 2018). The global critical P surplus and associated global soil P input for croplands was taken from Springmann et al. (2018) and Zhang et al. (2021a). Critical P surplus was calculated from total P loss to freshwater (calculated by multiplying critical concentrations of 50-100 mgP/m^3 with global freshwater discharge of 38 × 10^{12} m^3/yr, giving 1.9-3.8 TgP/yr) and estimates of human sewage contribution (1.2-2.4 TgP/yr, leaving 0.7-1.4 TgP/yr from agricultural losses), P retention in aquatic sediment(Zhang et al. 2021b)(20% of P load to freshwater retained by sediment, giving an allowable runoff from agriculture of 0.9-1.8 TgP/yr) and soils (80% of P surplus retained, giving allowable P surplus of 4.5-9.0 TgP/yr) (Zhang et al. 2021a) based on estimated fractions for food chain P use efficiency and sewage recycling (see supplementary materials of Springmann et al. (2018) and Zhang et al. (2021a) for further details).

These estimates are based on global budget calculations not taking into account spatial heterogeneity in soil types, soil erosion rates, and the effect of climate change on soil erosion, P runoff, and water flows. Work is now ongoing to improve these estimates by taking a similar approach to the N boundary by using spatially explicit models to determine subglobal critical versus current P surpluses and aggregating these to the global level, which could also allow interaction with the climate ESB to be explored. This modelling is currently lacking though, and so for now the calculations of Springmann et al. (2018) and Zhang et al. (2021a) provide a provisional first-order estimate for a safe P ESB with respect to agriculture and freshwater eutrophication. For the distribution of where exceeds the safe and just P boundary at a subglobal scale for Figure 3 in the main text, we use estimated P load to freshwater (Mekonnen and Hoekstra 2018) and water discharge (Fekete et al. 2001; Wisser et al. 2010) to estimate the global distribution of surface water P concentrations to compare with our subglobal boundary values.

Table S6. *Values and safe limits adopted by the EC for critical concentrations, total inputs, surplus, and losses to surface water & oceans for P with respect to triggering surface water eutrophication. Bold numbers indicate headline indicators. Values based on the cited sources, and illustrated in Figure S3.*

**P indicator**	**Natural baseline**	**Current excess above baseline**	**Safe limit**	**Unit**	**Source**
**Critical concen-trations**	~10	**-**	**50-100**	mgP/m^3	Springmann et al. (2018); Mekonnen & Hoekstra (2018)
Input to agricultural soils	~10 [natural] +5-10 [anthro]	~17 [mined] + ~6-17 [manure, crops-all]	16 (8-17) [w.r.t mined input; inc. 50% recycling & global rebalancing]	TgP/yr	Mekonnen & Hoekstra (2018); Springmann et al. (2018); Bouwman et al. (2013); Cordell & White (2014)
**Soil surplus**	-	**~10** (8.8-11.3) [crops] + ~1 [pasture]	**4.5-9.0** [based on 1.2-2.4 of 1.9-3.8 loss budget used by sewage etc.]	TgP/yr	Zhang et al. (2021a); Mekonnen & Hoekstra (2018); Bouwman et al. (2013); Cordell & White (2014)

Loss to surface water	~0.4 [dissolved]; ~2.5(-9.9) [total inc. particulate P]	~1.5(-3.4) [from anthro. inputs]: ~0.6 [agri.], ~0.5(-2.4) [sewage], ~0.4 [industry]; ~7.3 [total]: ~5.4 [agri.], ~1.7 [sewage/other]	1.9-3.8 [inc. ~0.4 natural, 1.5-3.4 without]	TgP /yr	Mekonnen & Hoekstra (2018); Carpenter & Bennett (2011); Zhang et al. (2021a); Zou et al. (2022); Van Drecht et al. (2009); Beusen et al. (2022)
Loss to Ocean	~0.4 [dissolved]; ~1.3(-9.3) [total inc. particulate P]	~1.5 [from anthro. inputs]; ~2.5-3.3 [total]	1.6(-11.2)? [20% natural]; 11(-100)? [10x low natural]	TgP /yr	Kemena et al. (2019); Beusen et al. (2022); Carpenter & Bennett (2011); Beusen et al. (2005); Seitzinger et al. (2010); Steffen et al. (2015)

As with the N ESB calculation, focusing for our headline indicator on global critical P surplus based on the more recent method of Springmann et al. (2018) has some advantages over the original PB quantification by taking account of other P sources such as sewage, focusing directly on P surplus and P losses and so not incorporating an implicit PUE (which also makes clear the role of improving PUE in transformation), and allowing additional P inputs in under-fertilised regions (increasing the global safe P limit). On the other hand, emphasis is placed on agricultural losses while the equally or even more significant pathway via sewage to freshwater is assumed to remain constant when setting the safe soil surplus and loss to freshwater (Table S6, Figure S3). Efforts to improve sewage recycling within agriculture to close the nutrient loop would therefore effectively increase the safe limits for agricultural losses. At a subglobal scale P loss to surface water is more relevant as this can be more decoupled to P surplus than for N surplus and loss as a result of spatially heterogeneous soil erosion rates.

It should be noted that all PBs derived until now imply an implicit direct relationship between P application and P loss. However, as stated above, unlike N, P is adsorbed in the soil and P concentrations in solution are governed by soil P contents. Changes in P contents are thus the cause of changes in P leaching and P runoff to surface water, which are reacting with a delay time to changes in P input. The PBs given in Springmann et al (2018) include an ongoing soil and sediment P retention, while this would not be acceptable at an infinite time scale. An alternative approach for assessing the PBs would be to link it to sustainable P management based on the “Build-up or mining and Maintenance” approach (Li et al. 2011; Bai et al. 2013). The principle of this approach, which is applied in fertiliser recommendation systems and P fertiliser policy, is that P application should be (i) more than the P withdrawal in harvested crop when available soil P < critical level for crop yield, to build up available soil P to the required agronomic level asses, and (ii) equal the P withdrawal in harvested crops when available soil P equals the critical level for crop yield (Li et al. 2011). This applies the assessment of a temporary additional P input for a target period followed by a P input equal to crop P demand, which implies then a near 100% P use efficiency, when the soil P status is at an adequate level. Estimates are currently ongoing but were not yet ready to be included in this overview.

A safe limit for P loss to the ocean is not provided by this approach and is more uncertain in the literature. Rockström et al. (2009) suggest ~20% above natural baseline for long timescales as a low limit and 10x natural baselines as a high limit based on past ocean anoxia events, while Watson et al. (2017) suggest

double background weathering over 50ky is enough to trigger global ocean anoxia within 10ky, and Kemena et al. (2019) find that a RCP8.5 warming scenario (driving a 2.5x increase in P weathering) causes a 5x increase in ocean suboxic volume (greater than that caused by direct anthropogenic P release to the ocean) but no global ocean anoxia event. In contrast, preliminary modelling for this study using the same UVic ESCM Earth system model setup as Kemena et al. (2019) suggests that ocean P inputs that are even modestly above background levels could cause significant Earth system disruption if continuously maintained beyond 1000 years, suggesting that long-term safe limits for P loss to the ocean might be close to zero. Coastal hypoxia is occurring far sooner and faster than open ocean hypoxia, but modelling of future coastal hypoxia scenarios is lacking at a global scale. More research is therefore required in order to assess whether the safe limits for P surplus and loss to terrestrial surface water are also safe for the ocean.

P pollution has no direct human health effects, with many countries not setting a maximum phosphate limit in water supplies. However, as with N pollution, harm can occur as a result of eutrophication, and so we have set the harm boundary for P the same as the safe boundary. A further consideration is that unlike N, the primary source of P for agriculture is a non-renewable resource (rock phosphate). Rock phosphate depletion presents potential inter-generational harm through inefficient usage prior to the establishment of full nutrient recycling (see SM section 2.4.4), while unequal access due to both poverty and geographic distribution is also a justice issue (Elser and Bennett 2011).

Table S7. *Current values and proposed safe limits for critical concentrations, total inputs, new fixation, surpluses, and losses for N in relation to the protection of surface water (from N runoff), terrestrial ecosystems (from NH_3 emissions and related N deposition), and drinking water (from NO_3 leaching). Bolded rows indicate those used to derive values in Table S5.*

N indicator & environmental system		Current global value	Safe limit	Unit	Timeframe for current value	Domain	Source
Critical concen-trations or depos-ition	Surface water (N runoff)	1.63	1-2.5	mgN/L	-	-	de Vries et al. (2013)
	Groundwater (NO_3 leach)		50 [11.3]	mgNO_3/L [mgNO_3-N/L]	-	-	de Vries et al. (2013)
	Terr. ecosystems (NH_3 emiss.)	0.56	1-3 (air)	ugNH_3/m^3	-	-	de Vries et al. (2013)
	Terr. ecosystems (NH_3 emiss.)		5-25 (biome dependent)	kgN/ha/yr	-	-	de Vries et al. (2021)
	**Surface water (N runoff)**		**2.5 (1-4)**	**mgN/L**	**-**	**-**	**Schulte-Uebbing et al.** (2022)
	**Groundwater (NO_3 leach)**		**50 [11.3]**	**mgNO_3/L [mgNO_3-N/L]**	**-**	**-**	**Schulte-Uebbing et al.** (2022)
	**Terr. ecosystems (NH_3 emiss.)**		**5-20 (biome dependent)**	**kgN/ha/yr**	**-**	**-**	**Schulte-Uebbing et al.** (2022)
Critical global N inputs	-	248		TgN/yr	2000	Cropland & pasture	Bouwman et al. (2013)
	-	185	~95?	TgN/yr	2010	Cropland & pasture	Bodirsky et al. (2014)
	-	174	-	TgN/yr	2010	Cropland & pasture	Zhang et al. (2015)
	Surface water (N runoff)	191		TgN/yr	2002-2010	Cropland	Mekonnen & Hoekstra (2015)

	Surface water (N runoff)	167-208		TgN/yr	2010	Cropland	Springmann et al. (2018)
	-	161 (139–192)		TgN/yr	2010 (2005-2014)	Cropland	Zhang et al. (2021b)
	Terr. ecosystems (NH_3 emiss.)	232	220	TgN/yr	2010	Cropland & improved pasture	Schulte-Uebbing et al. (2022)
	Groundwater (NO_3 leach)	232	245	TgN/yr	2010	Cropland & improved pasture	Schulte-Uebbing et al. (2022)
	Surface water (N runoff)	232	198	TgN/yr	2010	Cropland & improved pasture	Schulte-Uebbing et al. (2022)
	**Combined (balanced)**	**232**	**134**	**TgN/yr**	**2010**	**Cropland & improved pasture**	**Schulte-Uebbing et al.** (2022)
**Critical new N fixation**	Combined	140	35	TgN/yr	~2000	All anthropogenic N fixation	Rockström et al. (2009)
	-	122		TgN/yr	2000	Cropland & pasture	Bouwman et al. (2013)
	-	136		TgN/yr	"Begin. 21st Century"	All agriculture	Fowler et al. (2013)
	Surface water (N runoff)	140	72 (62-82)	TgN/yr	2000	Cropland & pasture	de Vries et al. (2013)
	Terr. ecosystems (NH_3 emiss.)	140	102 (89-115)	TgN/yr	2000	Cropland & pasture	de Vries et al. (2013)
	Surface water (N runoff)	150	62 (62-82)	TgN/yr	~2000	Cropland & pasture	Steffen et al. (2015)

	Surface water (N runoff)	130		TgN/yr	2002-2010	Cropland	Mekonnen & Hoekstra (2015)
	Surf. Water (balanced)	132	69 (52-113)	TgN/yr	2010	Cropland	Springmann et al. (2018)
	Surf. Water (unbalanced)	132	52-69	TgN/yr	2010	Cropland	Springmann et al. (2018)
	-	86 (68–97)		TgN/yr	2010 (2005-2014)	Cropland	Zhang et al. (2021b)
	Terr. ecosystems (NH_3 emiss.)	122	106	TgN/yr	2010	Cropland & improved pasture	Schulte-Uebbing et al. (2022)
	Groundwater (NO_3 leach)	122	107	TgN/yr	2010	Cropland & improved pasture	Schulte-Uebbing et al. (2022)
	Surface water (N runoff)	122	92	TgN/yr	2010	Cropland & improved pasture	Schulte-Uebbing et al. (2022)
	**Combined (balanced)**	**122**	**65**	**TgN/yr**	**2010**	**Cropland & improved pasture**	**Schulte-Uebbing et al.** (2022)
**Critical N surplus**	-	138 (93 arable, 45 grass)		TgN/yr	2000	Cropland & pasture	Bouwman et al. (2013)
	Surface water (N runoff)	159	50-100	TgN/yr	2010	Cropland & pasture	Bodirsky et al. (2014)
	-	78	39	kgN/ha/yr	2010	Cropland	Zhang et al. (2015)
	**-**	100	50	TgN/yr	2010	Cropland	Zhang et al. (2015)

	Surface water (N runoff)	108		TgN/yr	2002-2010	Cropland	Mekonnen & Hoekstra (2015)
	Surf. Water (unbalanced)	134	67-90	TgN/yr	2010	Cropland	Springmann et al. (2018)
	Surf. Water (balanced)	134	90 (67-146)	TgN/yr	2010	Cropland	Springmann et al. (2018)
	Surf. Water (unbalanced)	-	52-69	kgN/ha/yr	2010 (2005-2014)	Cropland	Zhang et al. (2021a)
	Surface water (N runoff)	144	106-123	TgN/yr	2010	Cropland & pasture	Chang et al. (2021)
	Terr. ecosystems (NH_3 emiss.)	119	101	TgN/yr	2010	Cropland & improved pasture	Schulte-Uebbing et al. (2022)
	Groundwater (NO_3 leach)	119	117	TgN/yr	2010	Cropland & improved pasture	Schulte-Uebbing et al. (2022)
	Surface water (N runoff)	119	92	TgN/yr	2010	Cropland & improved pasture	Schulte-Uebbing et al. (2022)
	**Combined (balanced)**	**119**	**57**	**TgN/yr**	**2010**	**Cropland & improved pasture**	**Schulte-Uebbing et al.** (2022)
**Critical N losses**	Surface water (N runoff)	10.7	5.4-7.2	TgN/yr	2000	Cropland & pasture	de Vries et al. (2013)
	Terr. ecosystems (NH_3 emiss.)	34	24.9-32.1	TgN/yr	2000	Cropland & pasture	de Vries et al. (2013)
	All Agri leach & runoff	57		TgN/yr	2000	Cropland & pasture	Bouwman et al. (2013)
	NH_3 volatilization	24		TgN/yr	2000	Cropland & pasture	Bouwman et al. (2013)

	Agri to SW	24.4		TgN/yr	2002-2010	Cropland	Mekonnen & Hoekstra (2015)
	Sewage to SW	7.5		TgN/yr	2002-2010	Sewage	Mekonnen & Hoekstra (2015)
	Industry to SW	0.74		TgN/yr	2002-2010	Industry	Mekonnen & Hoekstra (2015)
	**Terr. ecosystems (NH_3 emiss.)**	32	**29**	**TgN/yr**	**2010**	**Cropland & improved pasture**	**Schulte-Uebbing et al.** (2022)
	**Groundwater (NO_3 leach)**	37	**34**	**TgN/yr**	**2010**	**Cropland & improved pasture**	**Schulte-Uebbing et al.** (2022)
	**Surface water (N runoff)**	23	**21**	**TgN/yr**	**2010**	**Cropland & improved pasture**	**Schulte-Uebbing et al.** (2022)

Table S8. *Current values and proposed safe limits for local critical concentrations, total input, surplus, and losses to freshwater & oceans for P. Natural baselines are also provided where available, and the current global value indicates the excess above this baseline (unless otherwise stated). Bolded rows indicate those used to derive values in Table S6 or Figure S3.*

**P indicator**	**Natural baseline**	**Current global value (excess above baseline)**	**Safe limit**	**Unit**	**Timeframe for current value**	**Domain**	**Source**
**Critical concen-trations**			24-160	mgP/ m^3			Carpenter & Bennett (2011)
			**50-100**	**mgP/ m^3**			**Springmann et al.** (2018)
**Input of mined P to agricultural soils**	**10-15 (+5 anthro. weather-ing)**	22.6		**TgP/yr**	**~2000-2008**	**All soil**	**Carpenter & Bennett** (2011)
		~40 (inc. weathering)	3.72 (0.96-3.72) [for 24 mgP/m^3 limit]; 26.2 (6.73-26.2) [for 160 mgP/m^3 limit]	TgP/yr	~2000-2008	Erodible soil	Carpenter & Bennett (2011)
		14.2		TgP/yr	2000	Cropland	MacDonald et al. (2011)
		14.0		TgP/yr	2000	Cropland & pasture	Bouwman et al. (2013)
		16.5		TgP/yr	~2000-2010	Cropland & livestock	Cordell & White (2014)
		14.2	6.2 (6.2-11.2)	TgP/yr	~2000-2008	Erodible soils	Steffen et al. (2015)
		17.0		TgP/yr	2013	Cropland	Bouwman et al. (2017)
		**17.0**		**TgP/yr**	**2002-2010**	**Cropland**	**Mekonnen & Hoekstra** (2018)
		17.8	6-12	TgP/yr	2010	Cropland (no recycling)	Springmann et al. (2018)

		17.8	8-16	TgP/yr	2010	Cropland (50% recycling)	Springmann et al. (2018)
		**17.8**	16 (8-17)	**TgP/yr**	**2010**	**Cropland (recycling + balancing)**	**Springmann et al.** (2018)
		16.4		TgP/yr	~2010 (2005-2014)	Cropland	Zou et al. (2022)
**Total P Input to agricultural soils (including manure)**		23.8 (14.2 min., 9.6 man.)		TgP/yr	2000	Cropland	MacDonald et al. (2011)
		**31 (14 min., 17 man.)**		**TgP/yr**	**2000**	**Cropland & pasture**	**Bouwman et al.** (2013)
		32.5 (16.5 min., 16 man.)		TgP/yr	~2000-2010	Cropland & pasture	Cordell & White (2014)
		25 (17 min. +6 man.)		TgP/yr	2013	Cropland	Bouwman et al. (2017)
		**24.1 (17 min., 5.9 man., 1.2 irrig.)**		**TgP/yr**	**2002-2010**	**Cropland**	**Mekonnen & Hoekstra (2018)**
		22.5 (16.4 min., 6 man.)		TgP/yr	~2010 (2005-2014)	Cropland	Zou et al. (2022)
**Soil surplus**		12 (11 crop +1 pasture)		TgP/yr	2000	Cropland & pasture	Bouwman et al. (2013)
		12 (9 crop +1 pasture)		TgP/yr	~2000-2010	Cropland & pasture	Cordell & White (2014)
		9		TgP/yr	2013	Cropland	Bouwman et al. (2017)
		6.8	3.5-6.9	kgP/ha/yr	~2010 (2005-2014)	Cropland	Zhang et al. (2021a)

		**8.8**	4.5-9.0	**TgP/yr**	**~2010 (2005-2014)**	**Cropland**	**Zhang et al.** (2021a)
		11.3		**TgP/yr**	**2002-2010**	**Cropland**	**Mekonnen & Hoekstra (2018)**
**Loss to surface water**	~8-9.9?	9.9 (9.9-38.4) [TP, inc. natural]	8.6 (1.2-8.6)	TgP/yr	~2000-2008	All soil	Carpenter & Bennet (2011)
		4 [runoff]		**TgP/yr**	2000	Cropland & pasture	Bouwman et al. (2013)
		~1.3 [sewage]		TgP/yr	~2000-2010	Cropland & pasture	Cordell & White (2014)
	**~0.4 [from est. of natural concn.]**	**1.47 [TP from inputs]: 0.56 [agri], 0.91 [sewage & other]**		**TgP/yr**	**2002-2010**	**Cropland**	**Mekonnen & Hoekstra (2018)**
		**6.3 [erosion/PP, 1.3 (0.7-1.9) after 70-89% redposited]**		**TgP/yr**	**~2000**	**Cropland**	**Alewell et al.** (2020)
		**1.2-2.4 [sewage]**	**1.9-3.8 [All inputs], 0.7-1.4 [agri. only]**	**TgP/yr**	**~2010 (2005-2014)**	**Cropland**	**Zhang et al.** (2021a)
	**~2.5**	**~9.8 [TP, inc. natural], ~7.3 [exc. natural]: ~5.4 [agri], ~1.7 [sewage/other]**		**TgP/yr**	**2015**	**Cropland & pasture**	**Beusen et al.** (2022)
**Loss to Ocean**		~9 [inc. natural, PP]		TgP/yr	~2000	All land, incl. natural	Beusen et al. (2005)
	~2-4	~1.75		TgP/yr	~2020	All inputs	Filippelli (2008)

		8.6 [TP, inc. natural]: 6.6 [PP], 1.4 [DIP]; 0.6 [DOP]		TgP/yr	2000	All land, incl. natural	Seitzinger et al. (2010)
	8	9 (9-32) [TP, inc. natural], 1 (1-31) [TP, exc. natural]		TgP/yr	~2000-2008	All soil	Carpenter & Bennett (2011)
	1.1	22 [8-9 in SI?]	11 (11-100) [~1.3 strict]	TgP/yr	~2000-2008	All inputs	Steffen et al. (2015)
	**6.5 (1.5-9.3)**			**TgP/yr**	**pre-ind.**	**All land**	**Kemena et al.** (2019)
	**~1.3**	**~4.6 [TP, inc. natural], 3.3 [exc. natural]: ~2.1 [agri.], ~1.1 [sewage & other]**		**TgP/yr**	2015	**Cropland & pasture**	**Beusen et al. (2022)**

1.5. Aerosols and Air Pollution

Tiny solid and liquid particles (other than cloud droplets) suspended in the atmosphere are called aerosols. Natural sources, such as windblown dust, sea salts, volcanic ash, smoke from wildfires, and anthropogenic sources, such as sulphates, nitrates and soot particles (black carbon) from factories, traffic or traditional cookstoves are all examples of aerosols. Aerosols from natural sources dominate air pollution concentration in wild and pristine areas but anthropogenic sources occur where most people live, thus having large health impacts. Anthropogenic aerosols can be potentially controlled (Amann et al. 2020) to lower impacts on human health. Air pollutant concentrations vary depending on diverse factors including (seasonal) emissions, weather, and climate change (Mickley et al. 2004; Jacob and Winner 2009; Westervelt et al. 2016). In our assessment of safe and just (harm) limits, we have drawn on literature and guidance from international organisations.

The safe boundaries described here meet I1 justice criteria, while I2 and I3 are discussed and just (NSH) boundaries are quantified in section 2.3.5.

Aerosols have complex and mixed climate impacts (Forster, Storelvmo, et al. 2021). Aerosols affect the radiative balance directly by either absorbing (e.g. black carbon aerosols) or reflecting (e.g. sulphate aerosols) solar radiation, and indirectly by affecting cloud formation processes. Because of the complex interactions between the aerosols and climate, there is large uncertainty in estimating aerosol radiative forcing and the climate system response. Aerosols originate from diverse sources and have a lifetime of only about 10 days in the atmosphere, leading to large spatial and temporal heterogeneity in their distribution and impacts. Estimates of global aerosol optical depth (AOD; a measure of aerosol loading in the atmosphere) are ~0.13-0.14 (Boucher, Randall, et al. 2013; Vogel et al. 2022) with anthropogenic contribution to AOD ~0.03-0.04. The highly localised aerosol concentrations and their complex interactions with climate could trigger regional-scale tipping points or substantial adverse effects on the regional hydrological cycle. Steffen et al. (2015) proposed a regional boundary of 0.25 with a zone of uncertainty of 0.25 to 0.50 (an increase of ~0.1 from the background aerosol level due to human activities) for the AOD in the South Asian monsoon region that could lead to drier monsoon seasons.

In recent years, climate modelling studies have identified potential shifts in the location of Intertropical Convergence Zone (ITCZ) triggered by differences in sulphate aerosol optical depths (AOD) between the Northern and Southern hemisphere impacting regional monsoon systems (e.g., West African summer monsoon rainfall; (Haywood et al. 2013), and Indian monsoon; (Krishnamohan and Bala 2022). An imbalance in natural or anthropogenic AOD between the hemispheres could arise because of the difference in landmass and population, volcanic eruption or potential Solar Radiation Modification (SRM) (e.g., sulphate injections into the stratosphere). It is further recognized that AOD difference and its impact on shifts in tropical precipitation and water availability is sensitive to the aerosol particle size, and the latitudinal and altitudinal distribution of reflecting aerosols emissions (Zhao et al. 2021). The effects of interhemispheric imbalance of aerosol loading are such that a larger concentration of reflecting aerosols in one hemisphere leads to decreased precipitation in the tropical monsoon regions of the same hemisphere but increased precipitation in the tropical monsoon regions of the opposite hemisphere. Consistently, the decreases in global land monsoon precipitation from the 1950s to the 1980s are partly attributed to human-caused Northern Hemisphere aerosol emissions (IPCC 2021b). The observed reductions in South East Asian monsoon precipitation in the second half of the 20th century and Sahel drought in the 1980s are explained by a southward shift of the ITCZ due to inter-hemispheric temperature differences related to Northern Hemisphere cooling caused by increased Northern Hemisphere aerosol emissions (Douville,

Raghavan, Renwick, et al. 2021). Further, over South Asia, East Asia and Sahel, increases in monsoon precipitation due to warming from greenhouse gas emissions were counteracted by decreases in monsoon precipitation due to cooling from human-caused Northern Hemisphere aerosol emissions over the 20th century (IPCC 2021b).

Historic (and natural) examples of sulphate emissions leading to AOD difference between the hemispheres and subsequent rainfall deficits in the Sahel are the volcanic eruptions of El Chichon in the 1980s (AOD difference of 0.07) and Katmai (AOD difference of 0.08) (Haywood et al. 2013). Modelled sulphate injections into the stratosphere leading to interhemispheric sulphate AOD difference of ~ 0.2 have shown that the northern hemisphere monsoon index (summer monsoon rainfall in land regions of all northern hemisphere monsoon regions) could decline by ~10% and the summer monsoon precipitation over India could decline by >20% (Krishnamohan and Bala 2022). Similarly, Visioni et al. (2020), using modelled seasonal stratospheric aerosol injections simulate an annual interhemispheric AOD difference of ~0.1 (seasonal range 0.09-0.11), which leads to a decline in Indian monsoon precipitation and precipitation in the Amazon. Based on these recent studies, we assess that an additional interhemispheric difference in AOD of 0.05 to 0.20, caused mainly by anthropogenic aerosol emissions in the Northern Hemisphere, could lead to major disruptions to monsoon precipitation in the tropical monsoon regions. Therefore, we assign a safe boundary value of 0.15 for the difference in annual mean AOD between the two hemispheres. The current background annual mean interhemispheric difference in AOD is ~0.05, and this background difference reaches a high value of ~0.1 in the boreal spring and summers (Kishcha et al. 2009; Feng and Ramanathan 2010; Vogel et al. 2022) likely because of the increase in natural dust aerosol emissions in the northern hemisphere during the boreal spring and summers.

2. Justice analysis of the safe ESBs

2.1 Introduction

Damage to biophysical domains is causing harm to humans, communities and countries. Safe boundaries may protect humanity but not all humans, communities and countries. From a justice perspective we have to move beyond looking at humanity as a whole and/or average damage to identify just no significant harm (NSH) boundaries. This approach is part of our larger conceptualization and operationalization of Earth system justice in terms of ends and means (Gupta et al. 2023). In this paper we only look at the operationalization with respect to ends and within ends only with respect to ESBs.

Just NSH boundaries take primarily a bottom-up approach. For assessing NSH it was first important to assess what the threshold for significant harm should be. Based on the scholarship as well as on expert judgement of the Earth Commissioners we propose the definitions presented in the main text. These definitions are tentative formulations by the Earth Commission. The conceptualization of significant harm draws from similar conceptualizations in the fields of child protection legislation and medical scholarship and practice. The conceptualization of the NSH principle draws especially from the field of international environmental agreements, transboundary water agreements, and regional environmental regulations (e.g. EU green taxonomy)(Tignino and Bréthaut 2020). Despite several workshops within Working Group 4 of the Earth Commission where different thresholds were discussed we could not reach an agreement. What is clear is that the concept of leaving no one behind and a human rights approach in terms of harm was seen as too stringent by some and instead the IPCC approach of referring to harm to 10 and 100s of millions of people was seen as an easier threshold in relation to Earth system justice. This harm approach was then further incorporated in our exposure to harm calculations as explained in the methods section of the main paper. This approach is part of our larger 3 I approach explained below.

2.2 Operationalizing the “3 Is” framework

Our Just NSH approach is part of our broader justice narrative. It has procedural and substantive content. When applied to ESBs and transformations, it is divided into a focus on ends (boundaries and minimum access) and means (levers of transformation). In our assessment we use three justice criteria (3 I’s): Interspecies and Earth system stability (I1) (e.g. Burke and Fishel 2020); Intergenerational (between past and present I2a and present and future I2b) (Meyer 2021); and Intragenerational (I3) (Okereke 2006), including international (Blake and Smith 2022), inter-community (Bell 2020), and individual (e.g. Nickel 2021). We simplify these concepts to mean that there should be NSH to: (a) the functional ability of critical Earth systems to provide NCP and support species (I1); (b) current and future generations (I2a and b); and (c) the spatial and other distributive issues affecting current generations (I3), which can be further understood as: harm at the individual level; harm to sovereign nations and/or specific indigenous communities irrespective of size (international/inter-community). These criteria are used to assess proposals for Earth System Boundaries (Table S9). As can be seen, although it is critical from a human rights perspective not to undermine human rights at the individual level, our I1 criteria takes a much lower threshold. However, we try to address human rights issues at the local level through complementing the ESBs with WHO and other local level standards on air, soil and water pollution.

Table S9. *Criteria for 3I’s of justice*

**Domains of evaluation**	1. **Interspecies** justice and Earth system stability - **I1**	2. **Intergenerational** justice (temporal) (past to present, **I2a**; present to future, **I2b**)	3. **Intragenerational** justice (spatial) - **I3**		
			**International** justice	**Inter-community** justice	**Individual** justice
**Safe Earth System boundaries must…**	Ensure the stability of the Earth system from local to global levels to protect nature and humans	Intersectional justice (lens): Recognizing that harm can cumulate to some groups; the threshold of harm to these groups must be higher.			
		Minimise SH to future generations (while also recognizing past SH to present generations).	Minimise SH to nations within current generation	Not threaten any Indigenous Peoples’ or local communities’ existence	Not undermine human rights at the individual level

2.3 Assessing the safe ESBs

This section explains how we apply the concept of NSH to ESBs. We argue that ESBs should not cause, but minimise significant harm to humans.

2.3.1 Climate Change

While the safe boundary (headline 1.5°C, with other boundaries for other likelihoods of passing tipping points, Table 1) ensures I1 because it avoids tipping points, it raises challenges for I2a, I2b and I3. This is because significant harm to present generations, countries, indigenous and local communities, and individuals as well as other species can occur well before biophysical climate tipping elements are triggered.

In relation to **I2a**, we note that future generations are already committed to climate impacts. Intergenerational justice can be applied on time scales from successive generations to generations separated by centuries or millennia (Skillington 2019; Gupta et al. 2023). It is estimated that 190 million

people currently occupy global land below projected high tide lines for 2100 under low carbon emissions (Kulp and Strauss 2019), with further impacts likely to come on longer timescales. In relation to I2a, exposure to harm and harm from climate impacts experienced today stems from past emissions of greenhouse gases (Rocha et al. 2015). Moderate risks for climate-related mortality and morbidity, food production, water scarcity, and heat stress have already occurred in Africa, Europe, Australasia, and North America (IPCC 2021a). Moreover, even at current global warming, which are overlapping with the proposed safe ESBs, future generations are already committed to harm (e.g., small island states, marginalised groups, etc.) (see main text).

In relation to I3, as noted in the previous paragraph, climate impacts are being experienced by people worldwide today. Earlier IPCC assessments have reported harm to crops and water supply at warming as low as 0.5°C in some regions (IPCC 2022). We also highlight the understudied impacts of climate change on mental health, which may be affected negatively by heatwaves, loss of property and livelihoods due to floods, or climate-induced migration (Watts et al. 2019). In relation to international justice, ten island states are among the 15 countries that exhibit the highest disaster risk; this risk is increasingly determined by sea-level rise (Aleksandrova et al. 2021). In 2022, Pakistan experienced extreme rainfall inundating 1/3rd of the country and harming 33 million people. In addition, the frequency and intensity of dust storms in many dryland areas has increased over the last few decades partly due to climate-related factors, thus resulting in increasing negative impacts on human health in regions such as the Arabian Peninsula and broader Middle East, and Central Asia (IPCC 2019). In relation to inter-community justice, some indigenous peoples and local communities are harmed at current warming levels. Indigenous communities in the Arctic may also face harm already at 0.5°C since this level of warming poses risks to Arctic sea ice (IPCC 2022). Indigenous populations that rely on subsistence farming practices for food are also reported to have limited options for adapting to climate change threats (CSS 2021). The proposed safe ESBs will not protect these populations from being harmed. In relation to individual justice, we emphasise that the impacts of climate change (e.g. flooding, storms, droughts, and extreme heats) will be felt disproportionately by people whose access to resources is limited, mostly due to inadequate housing and infrastructure (Hallegatte and Rozenberg 2017). In Nigeria, for instance, the poorest 20% of people are 50% more likely to be affected by a flood, 130% more likely to be affected by a drought, and 80% more likely to be affected by a heat wave than the average Nigerian. Case studies in Bangladesh, India, and Honduras also suggest that poor people are losing two to three times more than non-poor people when hit by a flood or storm (Hallegatte and Rozenberg 2017). Lastly, it is estimated that climate change will cause cereal prices to increase with 1–29% by 2050 (IPCC 2019).

We determine that the safe ESB should be tightened to account for significant harm resulting from climate change. To inform the quantification of the just (NSH) boundary, we use the analysis of exposure to sea level rise and extreme heat presented in globally aggregated form in Fig. 2 and with summaries of greatest impacted countries in Table S10. We obtain a boundary of 1.0°C to avoid high exposure to significant harm from climate change (Table 1).

Table S10. Exposures of populations and countries to sea rise rise, extreme wet bulb temperatures and extreme mean annual temperatures. See Methods in main text for further information.

Temperature (°C)	Sea level rise				Exposure to at least 1 day per year with wet bulb temperature greater than 32°C			Exposure to at least 1 day per year with wet bulb temperature greater than 35°C			Exposure to MAT > 29°C
	Exposed pop, 2100	Exposed pop, long-term (committed, multi-centennial SLR)	Country with highest % pop affected; 2100 | long-term	No. of countries with > 25% of pop affected; 2100 | long-term	Population exposed (in millions of people)	Country with highest % pop exposed	No. of countries with > 50% of population exposed	Population exposed (in millions of people)	Country with highest % pop exposed	No. of countries with > 50% of population exposed	Population exposed (in millions of people)
0.5	Not assessed	Not assessed	Not assessed	Not assessed	370	Qatar (100%), United Arab Emirates (100%), Bahrain (98%)	4	50	Bahrain (28%), United Arab Emirates (21%), Oman (20%)	0	0
1.0	Not assessed	Not assessed	Not assessed	Not assessed	565	Qatar (100%), Bahrain (100%), United Arab Emirates (100%)	6	76	Bahrain (40%), Oman (26%), Central African Republic (25%)	0	0
1.5	~170M	~510M	Marshall Islands (69% | 88%)	7 | 23	761	Qatar (100%), Bahrain (100%), United Arab Emirates (100%)	7	111	United Arab Emirates (79%), Qatar (75%), Bahrain (50%)	3	221
2.0	~180M	~700M	Marshall Islands (70%) | Cocos Islands (96%)	7 | 40	1038	Qatar (100%), Bahrain (100%), United Arab Emirates (100%)	9	161	Bahrain (84%), United Arab Emirates (65%), Qatar (57%)	3	638

2.3.2 Biosphere

2.3.2.1 Functional integrity

The safe boundary for functional integrity meets most of our justice criteria. That is because functional integrity aims to preserve local Nature's Contributions to People (NCP) in agro-ecosystems, cities and other human dominated landscapes. Respecting the safe boundary thus prevents the loss of functional integrity, which would otherwise lead to, e.g., shortages of food production and deaths as result of flooding and landslides. It also prevents ecosystem fragmentation, simplification and area loss, which would otherwise trigger zoonoses (Keesing and Ostfeld 2021), such as COVID-19, Lyme disease, and the avian bird flu. In working lands, enhancing functional integrity secures access to pollination, pest control, and soil protection services which underpin food and nutritional security (Garibaldi et al. 2011, 2020; Chaplin-Kramer et al. 2014; Potts et al. 2016; Kremen and Merenlender 2018; Dainese et al. 2019). Functional integrity also reduces nutrients, pollutants, and sediments runoff from agricultural fields, which would otherwise impact water quality, human health and infrastructure. As a result, the integrity boundary ensures **I1, I2b and I3**. However, we note that people living in degraded lands require higher functional integrity in order to access NCP, but the burden to achieve this should be fairly shared.

In the specific case of integrity loss, both current and future generations of people and other species can benefit from meeting the 20-25% integrity boundary. However, areas with low functional integrity imply a loss to current generations through the behaviour of past generations.

Since the safe boundary for integrity meets the justice criteria, we propose to align the NSH boundary with the safe one.

2.3.2.2 Natural ecosystem area

Adherence to our proposed safe boundary for natural ecosystem area minimises harm with regards to I1 and I2b, but it raises concerns in regards to I2a and I3.

With respect to harm to current generations we note that this boundary entails restoring some 15% of the Earth's surface additional to the current global area of natural ecosystems. This raises the question of whether the safe proposal is not encumbered with the same objections as the Half Earth project. Notably, past proposals to conserve half of the land and sea to stop the loss of biodiversity (Locke 2014; Wilson 2016) have been critiqued for not addressing current equity issues including impacts on food production, increased land prices, land grabbing and displacement (Mehrabi et al. 2018), and the distributive challenges associated to the project (Ellis and Mehrabi 2019) potentially affecting a billion people (Schleicher et al. 2019). The natural ecosystem area boundary builds on an efficient protection of the most biodiversity rich habitats, where poor and vulnerable populations may be located. This means that particular countries, communities and individuals might incur higher costs associated with meeting the natural ecosystem area boundary, due to the biodiversity richness of the areas they populate. Thus, if it is essential to preserve 50-60% of the Earth's land and water we would argue from a justice perspective we should move away from the efficiency principle, and in order to avoid inequitable distribution of the responsibility the just boundary should be set at the upper end of the safe range. We signal that we differentiate between natural ecosystem area, which is a measure of biological quality, and protection, which is a legal measure. There is good evidence that intact areas can be inhabited, and that indeed, many intact areas are inhabited and have been managed by indigenous communities. However, retaining them intact may limit development options where conflicts between local justice (autonomy of choice at country, community and individual level) versus global justice (impacts of climate change) may intersect.

Based on this assessment, we consider that the just (NSH) boundary is aligned with the safe boundary, since ecosystem tipping points triggered by loss of natural ecosystem area leads to loss of Earth system NCP, **but that we need to take into account the distributional equity of intactness responsibilities (I2a and I3).** We argue that there is an optimum where enough nature is conserved to avoid significant harm to people and at the same time access to resources are provided to both current and future generations. We suggest that 55-60% of natural ecosystem area may need to be kept intact once equitable distribution of responsibilities are accounted for.

2.3.3 Freshwater

2.3.3.1 Surface water

The safe ESB for surface water ensures that 80% of the flows are reserved for nature which is sufficient for environmental needs. As a result, this boundary secures **I1 and I2b, except that we have not explicitly defined water quality standards.**

With respect to I2a, the surface water boundary requires additional consideration around water quality. Even if 20% of the surface water flows are left for humans after ensuring nature's needs, this does not mean that all the water left is either sufficient or of good quality, in which case we would not have met the ESB. This is an issue for both present and future generations. This could be solved by complementing the safe boundary with WHO standards on water quality (WHO 2017).

Regarding **I3** (inter-individual, inter-community and international justice), the boundary does not ensure equitable distribution. While the boundary can ensure greater water flows for downstream countries that are currently affected by large withdrawals by upstream countries, it would likely require the more than 800 transboundary water treaties that currently share water between riparian states be renegotiated. Within countries, communities located near water bodies might incur more harm from a 20% flow alteration boundary. Communities and regions located far away from the water bodies may have significantly more difficulties in accessing water. Assuming good water quality, the safe surface water boundary should therefore be complemented with principles of equitable distribution and transformation to reduce unsustainable levels of water use, in order to prevent harm to communities/countries, not least because the boundary requires a significant reduction in surface water demand in large parts of the world. Regarding **individual justice,** the safe surface water boundary does not take into account the conditions of the poor, women and children who are particularly water vulnerable—especially in remote dry areas (Prüss-Ustün et al. 2019).

2.3.3.2 Groundwater

The safe groundwater boundary is set to ensure that annual drawdown does not exceed annual recharge of groundwater aquifers.

In regard to **I1**, the groundwater recharge ESB ensures the protection of current ecosystems dependent on groundwater. Furthermore, imposing the condition of groundwater recharge being higher than withdrawal allows for aquifer restoration in the long term. This also meets **I2b** except in relation to quality concerns.

With respect to **I2a**, the groundwater boundary raises some concerns. Similar to the surface water boundary, the groundwater boundary does not ensure sufficient quantity nor good quality for human use.

Since the boundary is based on no net change of aquifer levels, it sets present/recent levels of recharge as the baseline, which fails to account for harm that is already caused to present generations.

In regard to **I3** (inter-community, inter-human and international justice)**,** the groundwater boundary has similar drawbacks as the surface water boundary. The safe ESB does not account for jurisdictional issues and can affect interstate cooperation on groundwater; and since groundwater aquifer shapes determine flows, it is not possible to assess how this boundary can affect interstate relations, however, it will likely require significant transformation by many states to constrain groundwater. Within countries, this may imply huge restrictions on water consumption (not water use) and affect the existing distribution of water through property rights, permits and contracts – which may all have to be renegotiated to ensure better distribution of scarce resources, a very expensive but necessary task to meet the ESB. Consequently, the safe boundary should be complemented with principles of equitable distribution of water resources in order to prevent harm to communities/countries. Regarding **individual justice**, the safe groundwater boundary does not take into account the vulnerability of particular individuals such as the poor, women and children (Prüss-Ustün et al. 2019). Without appropriate consideration in its implementation, these groups can still be harmed even if the safe groundwater boundary is met.

2.3.4 Nutrient cycles

In relation to I1 and I2, the proposed safe boundaries minimise significant harm because problematic eutrophication events in the Earth system are likely to occur well before direct harm to humans. The safe boundary therefore avoids direct human harm from drinking water with elevated N concentrations (Ward et al. 2018), and indirect harm through the consequences of eutrophication, such as loss of ecosystem services including lost fishery catches, impacts on farmland that rely on eutrophic water, and health impacts from algal blooms (Lavelle et al. 2005; Erisman et al. 2013; Cordell and White 2014; Schulte-Uebbing et al. 2022). The social cost of pollution by agricultural N in the EU in 2008 is estimated to have been €35−230 billion/yr, dwarfing the estimated economic benefit of N in primary agricultural production of €20−80 billion/yr (Van Grinsven et al. 2013). Since the safe boundaries intend to prevent eutrophication events, we expect that they therefore also minimise harm to present and future generations. However, future generations will become increasingly reliant on low grade phosphate rock which is more expensive to extract and has adverse effects on human, animal and plant health as it contains cadmium and other harmful contaminants (Gilbert 2009).

With respect to I3, the safe boundary presents some justice concerns. In relation to international justice, there are considerable differences in N and P use worldwide (Sutton et al. 2013). For example, Africa uses less than 14 kg of fertiliser per hectare while East Asia and Europe use 150 and 200 kg per ha respectively (Ministry of Agriculture, Livestock, Fisheries and Cooperatives 2020). These differences affect yields (Bonilla Cedrez et al. 2020). Moreover, plant-available phosphorus is unevenly distributed. Globally, 29% of croplands are considered deficient in plant-available phosphorus (Tirado and Allsopp 2012). Regionally, it is estimated that 75% of the agricultural soils are nutrient deficient in Sub-Saharan Africa, contributing to low agricultural productivity and food insecurity for 30% of the population (Cordell 2010). The loss of nutrients and soil organic C under current agricultural practices has affected 25% of soils in sub-Saharan Africa (Stewart et al. 2020). In contrast to the cost of over-fertilisation dominating in Europe, in 2007, degraded soils affected about 485 million African people and cost US $9.3 billion annually[29]. We advise taking into account these distributional issues when enforcing the safe boundaries. **In relation to inter-community and individual justice**, the extraction of phosphate rock harms specific communities, including the poor and marginalised individuals (Cordell and White 2014). Additionally, improper management can cause disproportionate harm on the poor and marginalised individuals, and ~7% of global phosphate supply is from the Western Sahara whose occupation and control of resources by Morocco is in

spite of UN resolutions (Cordell and White 2014). Under current agricultural practices it is estimated that remaining P will last 259 years (Brownlie et al. 2021). While the prospects of running out are not an immediate threat, the impacts of price volatility from phosphorus scarcity will hit those regions without adequate nutrients and impose input and food costs on the most vulnerable, similar to during the 2008 phosphate price spike (Khabarov and Obersteiner 2017).

Unlike other Earth system domains such as climate, a just (NSH) boundary based on direct harm is less strict than a NSH boundary based on indirect harm via triggering biophysical tipping elements. We therefore align the just (NSH) boundary with the safe boundaries for direct harm, which include local standards for N and P in air and soil (section 1.4). They should also and take into account distributional issues regarding access to fertilisers.

2.3.5 Aerosols and air pollution

The ESB on interhemispheric difference in aerosol optical depth aims to ensure earth system stability and interspecies justice (I1). It can also protect future generations (I2b) through focusing on avoiding shifts in precipitation patterns of monsoons. Thus, it accounts for the livelihoods of future generations. However, the global ESB and the proposed regional ESB are not stringent enough to protect current generations from adverse health impacts of air pollution (I2a and I3). Hence, we follow the latest World Health Organisation air quality guidelines for ambient air pollution.

$PM_{2.5}$ refers to tiny particles or droplets in the air that are two and a half microns or less in width. It is measured in terms of concentration (µg/m3) with different thresholds for average daily or annual exposure. High levels of $PM_{2.5}$ are associated with respiratory illnesses and premature deaths as well as heart problems and debilitating asthma, as they are able to travel deeply into the respiratory tract, reaching the lungs (affecting lung function and worsening other medical conditions). $PM_{2.5}$ is not a single specific chemical entity, but a mixture of particles from different sources with different chemical compositions. Precursors of $PM_{2.5}$ are sulphur dioxide (SO2), nitrogen oxides (NOx), volatile organic compounds (VOC), and ammonia (NH3).

We propose their interim targets as basis for likelihood ranges for the NSH boundary (Table S11) given the linear relationship between mortality risk and air pollution (ca. 6-8% per 10 ug/m^3 relative to baseline mortality risk, see (Chen and Hoek 2020). Yet, $PM_{2.5}$ does not cover toxicity of particles which vary from sources and places.

Table S11. *Annual exposure to $PM_{2.5}$ for 24-hour mean*

**Annual exposure to $PM_{2.5}$ µg/m^3**	**Description**
< 5	Very low likelihood of harm
5 – 10	Low likelihood of harm
10 – **15**	**Moderate likelihood of harm**
15 – 25	High likelihood of harm
25 – 35	Very high likelihood of harm
> 35	Extremely high likelihood of harm

Aerosols from natural sources dominate air pollution concentration in wild and pristine areas but anthropogenic sources occur in most populated regions and can be potentially controlled to lower impacts on human health (Amann et al. 2020). Given natural emissions, such as aeolian dust or wildfires cannot be avoided and their control is impracticable, significantly reduced life expectancy due to air pollution from natural sources will remain (Lelieveld et al. 2020). Because of natural sources, the most stringent levels of

WHO guidelines on clean air pollution cannot be met everywhere, except for a few places in the mountains or close to the sea. In 2019, 99% of the global population lived in areas where the target level of WHO air quality guidelines (annual mean $PM_{2.5}$ of 5 μg/m^3) was not achieved (WHO 2021). Air pollutants have been declining in many regions around the globe since 2000, however, the aerosol concentrations over Southeast Asia have been rising during this period (Quaas et al. 2022). Hence, we propose the moderate likelihood level of harm of 15 μg/m^3 of mean annual exposure to $PM_{2.5}$ aerosols (WHO proposes 15 μg/m^3 for 24-hour mean), similarly to US EPA standards secondary goal of 15 μg/m^3 (primary goal of 12 μg/m^3) and EU guidelines of max. 20 μg/m^3. Reducing annual average $PM_{2.5}$ concentrations from levels of 35 μg/m^3, common in many developing regions and cities to the WHO guideline level of 15 μg/m^3 could reduce air pollution-related mortality by approximately 12% (based on WHO (2006)). Reducing air pollution to the lower pollution levels would lead to higher reductions in premature deaths. However, no level of air pollution can be called absolutely safe (no harm) from a health perspective, and staying within these WHO limits does reduce health risks.

With respect to I3, the safe boundary aligns with just, yet air pollution is linked to health impacts in animals, crop yield impacts and pollination.

When considering regional and local aerosol levels and their impacts, local sources dominate. This can be linked to intragenerational injustices with regards to access to and means for clean technologies (Rao et al. 2021; Chen et al. 2022). When considering **international justice,** the ESB accounts for the fact that aerosol loading accumulates over large regions, even an entire hemisphere, to cause a global scale climate effect. Aerosols do not stop at national boundaries. Yet, short-lived climate forcers which affect human health most, are strongly linked to local sources (Hill et al. 2019). With respect to i**nter-community justice,** air pollution affects poor communities most as they tend to live and work in the most polluted areas and sectors (UNEP 2019; Poulhès and Proulhac 2022), have often no or little power to implement or guarantee compliance with standards of polluting industries close to them, and are often lacking technologies and fuels that mitigate the air pollution burden in their homes. Great inequalities exist in who contributes to and who suffers from air pollution (Rao et al. 2021). Regarding **individual justice,** air pollution adds to other pre-existing health burdens (e.g. Jorgenson et al. 2021; Raaschou-Nielsen et al. 2022) and susceptibility. For example, women, elderly, and children are suffering the most from indoor air pollution (Jerneck and Olsson 2013). Through the just boundary local impacts are accounted for.

SI References

Abatzoglou JT, Dobrowski SZ, Parks SA, Hegewisch KC. TerraClimate, a high-resolution global dataset of monthly climate and climatic water balance from 1958-2015. Sci Data. 2018 Jan 9;5:170191.

Achakulwisut P, Brauer M, Hystad P, Anenberg SC. Global, national, and urban burdens of paediatric asthma incidence attributable to ambient NO_2 pollution: estimates from global datasets. Lancet Planet Health. 2019 Apr;3(4):e166–78.

Acreman M. Environmental flows-basics for novices. WIREs Water. 2016 Sep;3(5):622–8.

Agutu NO, Awange JL, Ndehedehe C, Kirimi F, Kuhn M. GRACE-derived groundwater changes over Greater Horn of Africa: Temporal variability and the potential for irrigated agriculture. Sci Total Environ. 2019 Nov 25;693:133467.

Albrecht T, Winkelmann R, Levermann A. Glacial-cycle simulations of the Antarctic Ice Sheet with the Parallel Ice Sheet Model (PISM)–Part 1: Boundary conditions and climatic forcing. The Cryosphere. 2020a;14(2):599–632.

Albrecht T, Winkelmann R, Levermann A. Glacial-cycle simulations of the Antarctic Ice Sheet with the Parallel Ice Sheet Model (PISM)–Part 2: Parameter ensemble analysis. The Cryosphere. 2020b;14(2):633–56.

Aleksandrova M, Balasko S, Kaltenborn M, Malerba D, Mucke P, Neuschäfer O, et al. World Risk Report 2021 . Bündnis Entwicklung Hilft, Ruhr University Bochum – Institute for International Law of Peace and Conflict; 2021. Available from: https://weltrisikobericht.de/weltrisikobericht-2021-e.

Alewell C, Ringeval B, Ballabio C, Robinson DA, Panagos P, Borrelli P. Global phosphorus shortage will be aggravated by soil erosion. Nat Commun. 2020 Sep 11;11(1):4546.

Allan JR, Possingham HP, Atkinson SC, Waldron A, Di Marco M, Butchart SHM, et al. The minimum land area requiring conservation attention to safeguard biodiversity. Science. 2022 Jun 3;376(6597):1094–101.

Amann M, Kiesewetter G, Schöpp W, Klimont Z, Winiwarter W, Cofala J, et al. Reducing global air pollution: the scope for further policy interventions. Philos Trans A Math Phys Eng Sci. 2020 Oct 30;378(2183):20190331.

Andrén H. Effects of habitat fragmentation on birds and mammals in landscapes with different proportions of suitable habitat: A review. Oikos. 1994;71(3):355.

Armstrong McKay DI, Staal A, Abrams JF, Winkelmann R, Sakschewski B, Loriani S, et al. Exceeding 1.5°C global warming could trigger multiple climate tipping points. Science. 2022 Sep 9;377(6611):eabn7950.

Arthington AH. Environmental Flows: Saving Rivers in the Third Millennium. Berkeley, CA: University of California Press.; 2012.

Arthington AH, Bhaduri A, Bunn SE, Jackson SE, Tharme RE, Tickner D, et al. The Brisbane Declaration and Global Action Agenda on Environmental Flows (2018). Front Environ Sci Eng China . 2018;6. Available from: https://www.frontiersin.org/article/10.3389/fenvs.2018.00045

Arthington AH, Bunn SE, Poff NL, Naiman RJ. The challenge of providing environmental flow rules to sustain river ecosystems. Ecol Appl. 2006 Aug;16(4):1311–8.

Avelino J, Romero-Gurdián A, Cruz-Cuellar HF, Declerck FAJ. Landscape context and scale differentially impact coffee leaf rust, coffee berry borer, and coffee root-knot nematodes. Ecol Appl. 2012 Mar;22(2):584–96.

Bai X, Bjørn A, Kılkış Ş, Muñoz S, Whiteman G, Hoff H, et al. How to stop cities and companies causing planetary harm. Nature. 2022 Sep 12;609:463–6.

Bai Z, Li H, Yang X, Zhou B, Shi X, Wang B, et al. The critical soil P levels for crop yield, soil fertility and environmental safety in different soil types. Plant Soil. 2013 Nov 1;372(1):27–37.

Banks-Leite C, Pardini R, Tambosi LR, Pearse WD, Bueno AA, Bruscagin RT, et al. Using ecological thresholds to evaluate the costs and benefits of set-asides in a biodiversity hotspot. Science. 2014 Aug 29;345(6200):1041–5.

Beaudoing H, Rodell M, NASA/GSFC/HSL. GLDAS Noah Land Surface Model L4 3 hourly 0.25 x 0.25 degree, Version 2.1 . NASA Goddard Earth Sciences Data and Information Services Center; 2020. Available from: https://disc.gsfc.nasa.gov/datacollection/GLDAS_NOAH025_3H_2.1.html

Bell D. Communitarianism. In: Zalta EN, editor. The Stanford Encyclopedia of Philosophy. Metaphysics Research Lab, Stanford University; 2020.

Benchimol M, Peres CA. Widespread Forest Vertebrate Extinctions Induced by a Mega Hydroelectric Dam in Lowland Amazonia. PLoS One. 2015 Jul 1;10(7):e0129818.

Berkes F, Folke C, Colding J, editors. Linking social and ecological systems: management practices and social mechanisms for building resilience. Cambridge University Press; 2000.

Beusen AHW, Dekkers ALM, Bouwman AF, Ludwig W, Harrison J. Estimation of global river transport of sediments and associated particulate C, N, and P. Global Biogeochem Cycles . 2005 Dec;19(4). Available from: http://doi.wiley.com/10.1029/2005GB002453

Beusen AHW, Doelman JC, Van Beek LPH, Van Puijenbroek PJTM, Mogollón JM, Van Grinsven HJM, et al. Exploring river nitrogen and phosphorus loading and export to global coastal waters in the Shared Socio-economic pathways. Glob Environ Change. 2022 Jan 1;72:102426.

Beusen AHW, Van Beek LPH, Bouwman AF, Mogollón JM, Middelburg JJ. Coupling global models for hydrology and nutrient loading to simulate nitrogen and phosphorus retention in surface water – description of IMAGE–GNM and analysis of performance. Geosci Model Dev. 2015 Dec 21;8(12):4045–67.

Blake M, Smith PT. International Distributive Justice. In: Zalta EN, editor. The Stanford Encyclopedia of Philosophy . Summer 2022. Metaphysics Research Lab, Stanford University; 2022. Available from: https://plato.stanford.edu/archives/sum2022/entries/international-justice/

Bobbink R, Hicks K, Galloway J, Spranger T, Alkemade R, Ashmore M, et al. Global assessment of nitrogen deposition effects on terrestrial plant diversity: a synthesis. Ecol Appl. 2010 Jan;20(1):30–59.

Bodirsky BL, Popp A, Lotze-Campen H, Dietrich JP, Rolinski S, Weindl I, et al. Reactive nitrogen requirements to

feed the world in 2050 and potential to mitigate nitrogen pollution. Nat Commun. 2014 May 13;5:3858.

Bonilla Cedrez C, Chamberlin J, Guo Z, Hijmans RJ. Spatial variation in fertilizer prices in Sub-Saharan Africa. PLoS One. 2020 Jan 14;15(1):e0227764.

Boucher, Randall, et al. Clouds and Aerosols. Chapter 7. In: Stocker, T.F., D. Qin, G.-K. Plattner, M. Tignor, S.K. Allen, J. Boschung, A. Nauels, Y. Xia, V. Bex and P.M. Midgley, editor. Climate Change 2013: The Physical Science Basis Contribution of Working Group I to the Fifth Assessment Report of the Intergovernmental Panel on Climate Change (IPCC). Cambridge University Press, Cambridge, United Kingdom and New York, NY, USA.; 2013. p. 571–658.

Bouwman AF, Beusen AHW, Lassaletta L, van Apeldoorn DF, van Grinsven HJM, Zhang J, et al. Lessons from temporal and spatial patterns in global use of N and P fertilizer on cropland. Sci Rep. 2017 Jan 13;7:40366.

Bouwman L, Goldewijk KK, Van Der Hoek KW, Beusen AH, Van Vuuren DP, Willems J, et al. Exploring global changes in nitrogen and phosphorus cycles in agriculture induced by livestock production over the 1900–2050 period. Proc Estonian Acad Sci Biol Ecol. 2013;110(52):20882–7.

Brainerd E, Menon N. Seasonal effects of water quality: The hidden costs of the Green Revolution to infant and child health in India. J Dev Econ. 2014 Mar 1;107:49–64.

Breitburg D, Levin LA, Oschlies A, Grégoire M, Chavez FP, Conley DJ, et al. Declining oxygen in the global ocean and coastal waters. Science . 2018 Jan 5;359(6371). Available from: http://dx.doi.org/10.1126/science.aam7240

Brizga SO, Arthington AH, Pusey BJ, Kennard MJ, Mackay SJ, Werren GL, et al. Benchmarking, a “top-down” methodology for assessing environmental flows in Australian rivers. In: Proceedings of International Conference on Environmental Flows for Rivers. 2022.

Broadley A, Stewart-Koster B, Burford MA, Brown CJ. A global review of the critical link between river flows and productivity in marine fisheries. Rev Fish Biol Fish . 2022 Mar 24; Available from: https://doi.org/10.1007/s11160-022-09711-0

Broadley A, Stewart-Koster B, Kenyon RA, Burford MA, Brown CJ. Impact of water development on river flows and the catch of a commercial marine fishery. Ecosphere . 2020 Jul;11(7). Available from: https://onlinelibrary.wiley.com/doi/10.1002/ecs2.3194

Brownlie WJ, Sutton MA, Reay DS, Heal KV, Hermann L, Kabbe C, et al. Global actions for a sustainable phosphorus future. Nature Food. 2021 Feb 18;2(2):71–4.

Bruckerhoff LA, Leasure DR, Magoulick DD. Flow–ecology relationships are spatially structured and differ among flow regimes. J Appl Ecol. 2019 Feb;56(2):398–412.

Budiansky S. Extinction or miscalculation? Nature. 1994;370:105.

Bueler E, Brown J. Shallow shelf approximation as a “sliding law” in a thermomechanically coupled ice sheet model. Journal of Geophysical Research: Earth Surface . 2009;114(F3). Available from: https://agupubs.onlinelibrary.wiley.com/doi/abs/10.1029/2008JF001179

Bunn SE, Arthington AH. Basic principles and ecological consequences of altered flow regimes for aquatic biodiversity. Environ Manage. 2002 Oct;30(4):492–507.

Burke A, Fishel S. Across Species and Borders: Political Representation, Ecological Democracy and the Non-Human. In: Pereira JC, Saramago A, editors. Non-Human Nature in World Politics: Theory and Practice. Cham: Springer International Publishing; 2020. p. 33–52.

Carlisle DM, Nelson SM, Eng K. Macroinvertebrate community condition associated with the severity of streamflow alteration. River Res Appl. 2012;30(1):29–39.

Carlisle DM, Wolock DM, Meador MR. Alteration of streamflow magnitudes and potential ecological consequences: a multiregional assessment. Front Ecol Environ. 2010;9(5):264–70.

Carpenter SR. Eutrophication of aquatic ecosystems: bistability and soil phosphorus. Proc Natl Acad Sci U S A. 2005 Jul 19;102(29):10002–5.

Carpenter SR, Bennett EM. Reconsideration of the planetary boundary for phosphorus. Environ Res Lett. 2011 Feb 14;6(1):014009.

Chang J, Havlík P, Leclère D, de Vries W, Valin H, Deppermann A, et al. Reconciling regional nitrogen boundaries with global food security. Nature Food. 2021 Sep 13;2(9):700–11.

Chaplin-Kramer R, Dombeck E, Gerber J, Knuth KA, Mueller ND, Mueller M, et al. Global malnutrition overlaps with pollinator-dependent micronutrient production. Proc Biol Sci. 2014 Nov 7;281(1794):20141799.

Chaplin-Kramer R, O'Rourke ME, Blitzer EJ, Kremen C. A meta-analysis of crop pest and natural enemy response to landscape complexity. Ecol Lett. 2011 Sep;14(9):922–32.

Chase JM, Gooriah L, May F, Ryberg WA, Schuler MS. A framework for disentangling ecological mechanisms underlying the island species–area relationship. Frontiers of Biogeography; Oakland . 2019;11(1). Available from: http://dx.doi.org/10.21425/F5FBG40844

Chen J, Famigliett JS, Scanlon BR, Rodell M. Groundwater Storage Changes: Present Status from GRACE Observations. In: Cazenave A, Champollion N, Benveniste J, Chen J, editors. Remote Sensing and Water Resources. Cham: Springer International Publishing; 2016. p. 207–27.

Chen J, Hoek G. Long-term exposure to PM and all-cause and cause-specific mortality: A systematic review and meta-analysis. Environ Int. 2020 Oct;143:105974.

Chen L, Lin J, Martin R, Du M, Weng H, Kong H, et al. Inequality in historical transboundary anthropogenic $PM_{2.5}$ health impacts. Sci Bull Fac Agric Kyushu Univ. 2022 Feb 26;67(4):437–44.

Chen W, Olden JD. Evaluating transferability of flow-ecology relationships across space, time and taxonomy. Freshw Biol. 2018 Aug;63(8):817–30.

Connor EF, McCoy ED. Species-area relationships In Levin SA (Ed.), Encyclopedia of biodiversity, Vol. 5 (pp. 397--411). New York: Academic Press.[Google Scholar]; 2001.

Cordell D. The Story of Phosphorus. Sustainability implications of global phosphorus scarcity for food security [PhD in Sustainable Futures | PhD in Water and Environmental Studies]. Institute for Sustainable Futures, University of Technology, Sydney | Department of Water and Environmental Studies, Linköping University.; 2010 [cited 2022 Jun 6]. Available from: https://opus.lib.uts.edu.au/handle/10453/36078

Cordell D, White S. Life's Bottleneck: Sustaining the World's Phosphorus for a Food Secure Future. Annu Rev Environ Resour. 2014 Oct 17;39(1):161–88.

Couto TBA, Messager ML, Olden JD. Safeguarding migratory fish via strategic planning of future small hydropower in Brazil. Nature Sustainability. 2021 Jan 11;4(5):409–16.

CSS. Climate Change: Science and Impacts Factsheet . Center for Sustainable Systems, University of Michigan.; 2021. Report No.: Pub. No. CSS05-19. Available from: https://css.umich.edu/factsheets/climate-change-science-and-impacts-factsheet

Dainese M, Martin EA, Aizen MA, Albrecht M, Bartomeus I, Bommarco R, et al. A global synthesis reveals biodiversity-mediated benefits for crop production. Sci Adv. 2019 Oct;5(10):eaax0121.

Dang TD, Cochrane TA, Arias ME, Van PDT, de Vries TT. Hydrological alterations from water infrastructure development in the Mekong floodplains. Hydrol Process. 2016 Oct 15;30(21):3824–38.

Dankovic DA, Whittaker C. What is an acceptable risk of cancer due to occupational exposure to a carcinogen? (Abstract #1902). The Toxicologist, Supplement to Toxicological Sciences . 2015;144(1). Available from: https://www.toxicology.org/pubs/docs/Tox/2015Tox.pdf

DeClerck FAJ, Koziell I, Benton T, Garibaldi LA, Kremen C, Maron M, et al. A Whole Earth Approach to Nature-Positive Food: Biodiversity and Agriculture. In: von Braun J, Afsana K, Fresco LO, Hassan MHA, editors. Science and Innovations for Food Systems Transformation. Cham: Springer International Publishing; 2023. p. 469–96.

Denys C, Tscharntke T. Plant-insect communities and predator-prey ratios in field margin strips, adjacent crop fields, and fallows. Oecologia. 2002 Jan;130(2):315–24.

De Vries W, Du E, Bahl KB, Uebbing LS, Dentener F. Global-Scale Impact of Human Nitrogen Fixation on Greenhouse Gas Emissions. In: Oxford Research Encyclopedia of Environmental Science. 2017.

Díaz S, Zafra-Calvo N, Purvis A, Verburg PH, Obura D, Leadley P, et al. Set ambitious goals for biodiversity and sustainability. Science. 2020 Oct 23;370(6515):411–3.

Dinerstein E, Olson D, Joshi A, Vynne C, Burgess ND, Wikramanayake E, et al. An Ecoregion-Based Approach to Protecting Half the Terrestrial Realm. Bioscience. academic.oup.com; 2017 Jun 1;67(6):534–45.

Dinerstein E, Vynne C, Sala E, Joshi AR, Fernando S, Lovejoy TE, et al. A Global Deal For Nature: Guiding principles, milestones, and targets. Sci Adv. 2019 Apr;5(4):eaaw2869.

Döll P, Fiedler K. Global-scale modeling of groundwater recharge. Hydrol Earth Syst Sci. 2008 May 29;12(3):863–85.

Douville, Raghavan, Renwick, et al. Water Cycle Changes. Chapter 8. In: Masson-Delmotte, V., P. Zhai, A. Pirani, S.L. Connors, C. Péan, S. Berger, N. Caud, Y. Chen, L. Goldfarb, M.I. Gomis, M. Huang, K. Leitzell, E. Lonnoy, J.B.R. Matthews, T.K. Maycock, T. Waterfield, O. Yelekçi, R. Yu, and B. Zhou, editor. Climate Change 2021: The Physical Science Basis Contribution of Working Group I to the Sixth Assessment Report of the Intergovernmental Panel on Climate Change (IPCC). Cambridge University Press, Cambridge , United Kingdom and New York, NY, USA.; 2021. p. 1055–210.

Duffy KA, Schwalm CR, Arcus VL, Koch GW, Liang LL, Schipper LA. How close are we to the temperature tipping point of the terrestrial biosphere? Sci Adv . 2021 Jan;7(3). Available from: https://www.science.org/doi/10.1126/sciadv.aay1052

Dugan PJ, Barlow C, Agostinho AA, Baran E, Cada GF, Chen D, et al. Fish migration, dams, and loss of ecosystem services in the Mekong basin. Ambio. 2010 Jun;39(4):344–8.

Dunn FE, Darby SE, Nicholls RJ, Cohen S, Zarfl C, Fekete BM. Projections of declining fluvial sediment delivery to major deltas worldwide in response to climate change and anthropogenic stress. Environ Res Lett. 2019 Aug 6;14(8):084034.

Ellis EC, Mehrabi Z. Half Earth: promises, pitfalls, and prospects of dedicating Half of Earth's land to conservation. Current Opinion in Environmental Sustainability. 2019 Jun 1;38:22–30.

Elser J, Bennett E. A broken biogeochemical cycle. Nature. 2011;478(7367):29–31.

Erisman JW, Galloway JN, Seitzinger S, Bleeker A, Dise NB, Petrescu AMR, et al. Consequences of human modification of the global nitrogen cycle. Philos Trans R Soc Lond B Biol Sci. 2013 Jul 5;368(1621):20130116.

Erisman JW, Galloway J, Seitzinger S, Bleeker A, Butterbach-Bahl K. Reactive nitrogen in the environment and its effect on climate change. Current Opinion in Environmental Sustainability. 2011 Oct 1;3(5):281–90.

Fattorini S, Borges PAV. Species-area relationships underestimate extinction rates. Acta Oecol. 2012 Apr 1;40:27–30.

Fekete BM, Vörösmarty CJ, Lammers RB. Scaling gridded river networks for macroscale hydrology: Development, analysis, and control of error. Water Resour Res. 2001 Jul;37(7):1955–67.

Feng Y, Ramanathan V. Investigation of aerosol–cloud interactions using a chemical transport model constrained by satellite observations. Tellus B Chem Phys Meteorol. 2010 Jan 1;62(1):69–86.

Filippelli GM. The Global Phosphorus Cycle: Past, Present, and Future. Elements . 2008 Apr 1;4(2):89–95.

Forster, Storelvmo, et al. The Earth's Energy Budget, Climate Feedbacks, and Climate Sensitivity. Chapter 7. In: Masson-Delmotte, V., P. Zhai, A. Pirani, S.L. Connors, C. Péan, S. Berger, N. Caud, Y. Chen, L. Goldfarb, M.I. Gomis, M. Huang, K. Leitzell, E. Lonnoy, J.B.R. Matthews, T.K. Maycock, T. Waterfield, O. Yelekçi, R. Yu, and B. Zhou, editor. Climate Change 2021: The Physical Science Basis Contribution of Working Group I to the Sixth Assessment Report of the Intergovernmental Panel on Climate Change (IPCC). Cambridge University Press, Cambridge , United Kingdom and New York, NY, USA.; 2021. p. 923–1054.

Fowler D, Coyle M, Skiba U, Sutton MA, Cape JN, Reis S, et al. The global nitrogen cycle in the twenty-first century. Philos Trans R Soc Lond B Biol Sci. 2013 Jul 5;368(1621):20130164.

Fox-Kemper, Hewitt, Xiao, et al. Ocean, Cryosphere and Sea Level Change. Chapter 9. In: Masson-Delmotte, V., P. Zhai, A. Pirani, S.L. Connors, C. Péan, S. Berger, N. Caud, Y. Chen, L. Goldfarb, M.I. Gomis, M. Huang, K. Leitzell, E. Lonnoy, J.B.R. Matthews, T.K. Maycock, T. Waterfield, O. Yelekçi, R. Yu, and B. Zhou, editor. Climate Change 2021: The Physical Science Basis Contribution of Working Group I to the Sixth Assessment Report of the Intergovernmental Panel on Climate Change (IPCC). Cambridge University Press, Cambridge , United Kingdom and New York, NY, USA.; 2021. p. 1211–362.

Fremier AK, DeClerck FAJ, Bosque-Pérez NA, Carmona NE, Hill R, Joyal T, et al. Understanding Spatiotemporal Lags in Ecosystem Services to Improve Incentives. Bioscience. 2013 Jun 1;63(6):472–82.

Garibaldi LA, Oddi FJ, Miguez FE, Bartomeus I, Orr MC, Jobbágy EG, et al. Working landscapes need at least 20% native habitat. Conserv Lett. 2020;14(2):e12773.

Garibaldi LA, Steffan-Dewenter I, Kremen C, Morales JM, Bommarco R, Cunningham SA, et al. Stability of pollination services decreases with isolation from natural areas despite honey bee visits. Ecol Lett. 2011 Oct;14(10):1062–72.

Gatti LV, Basso LS, Miller JB, Gloor M, Gatti Domingues L, Cassol HLG, et al. Amazonia as a carbon source linked to deforestation and climate change. Nature. 2021 Jul;595(7867):388–93.

Gerten D, Hoff H, Rockström J, Jägermeyr J, Kummu M, Pastor AV. Towards a revised planetary boundary for consumptive freshwater use: role of environmental flow requirements. Current Opinion in Environmental Sustainability. 2013 Dec 1;5(6):551–8.

Gilbert N. Environment: The disappearing nutrient. Nature. 2009;461:716–8.

Gleeson T, Wang-Erlandsson L, Zipper SC, Porkka M, Jaramillo F, Gerten D, et al. The Water Planetary Boundary: Interrogation and Revision. One Earth. 2020 Mar 20;2(3):223–34.

Gonçalves-Souza D, Verburg PH, Dobrovolski R. Habitat loss, extinction predictability and conservation efforts in the terrestrial ecoregions. Biol Conserv. 2020 Jun 1;246:108579.

Grill G, Lehner B, Thieme M, Geenen B, Tickner D, Antonelli F, et al. Mapping the world's free-flowing rivers. Nature. 2019 May;569(7755):215–21.

Gu B, Zhang L, Van Dingenen R, Vieno M, Van Grinsven HJ, Zhang X, et al. Abating ammonia is more cost-effective than nitrogen oxides for mitigating $PM_{2.5}$ air pollution. Science. 2021 Nov 5;374(6568):758–62.

Gulev, Thorne, et al. Changing State of the Climate System. Chapter 2. In: Masson-Delmotte, V., P. Zhai, A. Pirani, S.L. Connors, C. Péan, S. Berger, N. Caud, Y. Chen, L. Goldfarb, M.I. Gomis, M. Huang, K. Leitzell, E. Lonnoy, J.B.R. Matthews, T.K. Maycock, T. Waterfield, O. Yelekçi, R. Yu, and B. Zhou, editor. Climate Change 2021: The Physical Science Basis Contribution of Working Group I to the Sixth Assessment Report of the Intergovernmental Panel on Climate Change (IPCC). Cambridge University Press, Cambridge , United Kingdom and New York, NY, USA.; 2021. p. 287–422.

Gupta J, Liverman D, Prodani K, Aldunce P, Bai X, Broadgate W, et al. Earth system justice needed to identify and live within Earth system boundaries. Nature Sustainability. 2023 Mar 2;1–9.

Hallegatte S, Rozenberg J. Climate change through a poverty lens. Nat Clim Chang. 2017 Apr 5;7(4):250–6.

Halley JM, Sgardeli V, Monokrousos N. Species-area relationships and extinction forecasts. Ann N Y Acad Sci. 2013 May;1286(1):50–61.

Hanasaki N, Inuzuka T, Kanae S, Oki T. An estimation of global virtual water flow and sources of water withdrawal for major crops and livestock products using a global hydrological model. J Hydrol. 2010 Apr 30;384(3):232–44.

Hanski I, Zurita GA, Bellocq MI, Rybicki J. Species-fragmented area relationship. Proc Natl Acad Sci U S A. 2013 Jul 30;110(31):12715–20.

Haywood JM, Jones A, Bellouin N, Stephenson D. Asymmetric forcing from stratospheric aerosols impacts Sahelian rainfall. Nat Clim Chang. 2013 Mar 31;3(7):660–5.

He F, Hubbell SP. Species–area relationships always overestimate extinction rates from habitat loss. Nature. 2011 May 18;473(7347):368–71.

Heinze C, Blenckner T, Martins H, Rusiecka D, Döscher R, Gehlen M, et al. The quiet crossing of ocean tipping points. Proc Natl Acad Sci U S A . 2021 Mar 2;118(9). Available from: http://dx.doi.org/10.1073/pnas.2008478118

Herbert C, Döll P. Global assessment of current and future groundwater stress with a focus on transboundary aquifers. Water Resour Res. 2019 Jun;55(6):4760–84.

Hill TD, Jorgenson AK, Ore P, Balistreri KS, Clark B. Air quality and life expectancy in the United States: An analysis of the moderating effect of income inequality. SSM Popul Health. 2019 Apr;7:100346.

Hoegh-Guldberg, Jacob, Taylor, et al. Impacts of 1.5ºC Global Warming on Natural and Human Systems. Chapter 3. In: Masson-Delmotte, V., P. Zhai, H.-O. Pörtner, D. Roberts, J. Skea, P.R. Shukla, A. Pirani, W. Moufouma-Okia, C. Péan, R. Pidcock, S. Connors, J.B.R. Matthews, Y. Chen, X. Zhou, M.I. Gomis, E. Lonnoy, T. Maycock, M. Tignor, and T. Waterfield, editor. Global Warming of 15°C An IPCC Special Report on the impacts of global warming of 15°C above pre-industrial levels and related global greenhouse gas emission pathways, in the context of strengthening the global response to the threat of climate change, sustainable development, and efforts to eradicate poverty. In Press; 2018.

Hubau W, Lewis SL, Phillips OL, Affum-Baffoe K, Beeckman H, Cuní-Sanchez A, et al. Asynchronous carbon sink saturation in African and Amazonian tropical forests. Nature. 2020 Mar;579(7797):80–7.

IFRC. World Disaster Report 2020: Come Heat of High Water . International Federation of Red Cross and Red Crescent Societies: Geneva, Switzerland; 2020. Available from: https://www.ifrc.org/document/world-disasters-report-2020

IPCC. Managing the Risks of Extreme Events and Disasters to Advance Climate Change Adaptation. A Special Report of Working Groups I and II of the Intergovernmental Panel on Climate Change. Field, C.B., V. Barros, T.F. Stocker, D. Qin, D.J. Dokken, K.L. Ebi, M.D. Mastrandrea, K.J. Mach, G.-K. Plattner, S.K. Allen, M. Tignor, and P.M. Midgley, editor. Cambridge University Press, Cambridge, UK and New York, NY, USA.; 2012.

IPCC. Climate Change and Land: an IPCC special report on climate change, desertification, land degradation, sustainable land management, food security, and greenhouse gas fluxes in terrestrial ecosystems. P.R. Shukla, J. Skea, E. Calvo Buendia, V. Masson-Delmotte, H.-O. Pörtner, D. C. Roberts, P. Zhai, R. Slade, S. Connors, R. van Diemen, M. Ferrat, E. Haughey, S. Luz, S. Neogi, M. Pathak, J. Petzold, J. Portugal Pereira, P. Vyas, E. Huntley, K. Kissick, M. Belkacemi, J. Malley, editor. In Press; 2019.

IPCC. Climate Change 2021: The Physical Science Basis. Contribution of Working Group I to the Sixth Assessment Report of the Intergovernmental Panel on Climate Change. Masson-Delmotte, V., P. Zhai, A. Pirani, S.L. Connors, C. Péan, S. Berger, N. Caud, Y. Chen, L. Goldfarb, M.I. Gomis, M. Huang, K. Leitzell, E. Lonnoy, J.B.R. Matthews, T.K. Maycock, T. Waterfield, O. Yelekçi, R. Yu, B. Zhou, editor. Cambridge University Press, Cambridge, United Kingdom and New York, NY, USA, In press; 2021a.

IPCC. Summary for Policymakers. In: Masson-Delmotte, V., P. Zhai, A. Pirani, S.L. Connors, C. Péan, S. Berger, N. Caud, Y. Chen, L. Goldfarb, M.I. Gomis, M. Huang, K. Leitzell, E. Lonnoy, J.B.R. Matthews, T.K. Maycock, T. Waterfield, O. Yelekçi, R. Yu, and B. Zhou, editor. Climate Change 2021: The Physical Science Basis Contribution of Working Group I to the Sixth Assessment Report of the Intergovernmental Panel on Climate Change (IPCC). Cambridge University Press, Cambridge , United Kingdom and New York, NY, USA.; 2021b. p. 3–32.

IPCC. Climate Change 2022: Impacts, Adaptation, and Vulnerability. Contribution of Working Group II to the Sixth Assessment Report of the Intergovernmental Panel on Climate Change. H.-O. Pörtner, D.C. Roberts, M. Tignor, E.S. Poloczanska, K. Mintenbeck, A. Alegría, M. Craig, S. Langsdorf, S. Löschke, V. Möller, A. Okem, B. Rama, editor. Cambridge University Press; 2022.

Jacob DJ, Winner DA. Effect of climate change on air quality. Atmos Environ. 2009 Jan 1;43(1):51–63.

Jerneck A, Olsson L. A smoke-free kitchen: initiating community based co-production for cleaner cooking and cuts in carbon emissions. J Clean Prod. 2013 Dec 1;60:208–15.

Jorgenson AK, Thombs RP, Clark B, Givens JE, Hill TD, Huang X, et al. Inequality amplifies the negative association between life expectancy and air pollution: A cross-national longitudinal study. Sci Total Environ. 2021 Mar 1;758:143705.

Joughin I, Smith BE, Medley B. Marine ice sheet collapse potentially under way for the Thwaites Glacier Basin, West Antarctica. Science. 2014 May 16;344(6185):735–8.

Jung M, Arnell A, de Lamo X, García-Rangel S, Lewis M, Mark J, et al. Areas of global importance for conserving terrestrial biodiversity, carbon and water. Nat Ecol Evol. 2021 Nov;5(11):1499–509.

Karp DS, Chaplin-Kramer R, Meehan TD, Martin EA, DeClerck F, Grab H, et al. Crop pests and predators exhibit inconsistent responses to surrounding landscape composition. Proc Natl Acad Sci U S A. 2018 Aug 14;115(33):E7863–70.

Kaufman D, McKay N, Routson C, Erb M, Dätwyler C, Sommer PS, et al. Holocene global mean surface temperature, a multi-method reconstruction approach. Sci Data. 2020 Jun 30;7(1):201.

Keesing F, Ostfeld RS. Impacts of biodiversity and biodiversity loss on zoonotic diseases. Proc Natl Acad Sci U S A . 2021 Apr 27;118(17). Available from: http://dx.doi.org/10.1073/pnas.2023540118

Kemena TP, Landolfi A, Oschlies A, Wallmann K, Dale AW. Ocean phosphorus inventory: large uncertainties in future projections on millennial timescales and their consequences for ocean deoxygenation. Earth Syst Dyn. 2019 Sep 6;10(3):539–53.

Khabarov N, Obersteiner M. Global Phosphorus Fertilizer Market and National Policies: A Case Study Revisiting the 2008 Price Peak. Front Nutr. 2017 Jun 14;4:22.

King J, Louw D. Instream flow assessments for regulated rivers in South Africa using the Building Block Methodology. Aquat Ecosyst Health Manag. 1998 Jan 1;1(2):109–24.

Kishcha P, Starobinets B, Kalashnikova O, Long CN, Alpert P. Variations of meridional aerosol distribution and solar dimming. J Geophys Res . 2009 May 22;114(D00D14). Available from: http://doi.wiley.com/10.1029/2008JD010975

Klein AM, Steffan-Dewenter I, Tscharntke T. Fruit set of highland coffee increases with the diversity of pollinating bees. Proc Biol Sci. 2003a May 7;270(1518):955–61.

Klein AM, Steffan-Dewenter I, Tscharntke T. Pollination of Coffea canephora in relation to local and regional agroforestry management. J Appl Ecol. 2003b Sep 26;40(5):837–45.

Klein AM, Steffan-Dewenter I, Tscharntke T. Foraging trip duration and density of megachilid bees, eumenid wasps and pompilid wasps in tropical agroforestry systems. J Anim Ecol. 2004 May;73(3):517–25.

Kremen C. Reframing the land-sparing/land-sharing debate for biodiversity conservation. Ann N Y Acad Sci. 2015 Oct;1355(1):52–76.

Kremen C, Iles A, Bacon C. Diversified farming systems: An agroecological, systems-based alternative to modern industrial agriculture. Ecol Soc . 2012;17(4). Available from: http://www.jstor.org/stable/26269193

Kremen C, Merenlender AM. Landscapes that work for biodiversity and people. Science . 2018 Oct 19;362(6412). Available from: http://dx.doi.org/10.1126/science.aau6020

Kremen C, Williams NM, Aizen MA, Gemmill-Herren B, LeBuhn G, Minckley R, et al. Pollination and other ecosystem services produced by mobile organisms: a conceptual framework for the effects of land-use change. Ecol Lett. 2007 Apr;10(4):299–314.

Kremen C, Williams NM, Bugg RL, Fay JP, Thorp RW. The area requirements of an ecosystem service: crop pollination by native bee communities in California. Ecol Lett. 2004 Sep 22;7(11):1109–19.

Krishnamohan KS, Bala G. Sensitivity of tropical monsoon precipitation to the latitude of stratospheric aerosol injections. Clim Dyn . 2022 Jan 5; Available from: https://doi.org/10.1007/s00382-021-06121-z

Kulp SA, Strauss BH. New elevation data triple estimates of global vulnerability to sea-level rise and coastal flooding. Nat Commun. 2019 Oct 29;10(1):4844.

Lavelle P, Dugdale R, Scholes R, Berhe AA, Carpenter E, Codispoti L, et al. Nutrient cycling. In: Ecosystems and Human Well-Being: Current State and Trends: Findings of the Condition and Trends Working Group (Millennium Ecosystem Assessment). Island Press, Washington, Covelo, London.; 2005.

Leadley P, Archer E, Bendandi B, Cavender-Bares J, Davalos L, DeClerck F, et al. Setting ambitious international restoration objectives for terrestrial ecosystems for 2030 and beyond. PLOS Sustainability and Transformation. 2022 Dec 6;1(12):e0000039.

Leahy SM, Robins JB. River flows affect the growth of a tropical finfish in the wet-dry rivers of northern Australia, with implications for water resource development. Hydrobiologia. 2021 Oct 1;848(18):4311–33.

Lelieveld J, Evans JS, Fnais M, Giannadaki D, Pozzer A. The contribution of outdoor air pollution sources to premature mortality on a global scale. Nature. 2015 Sep 17;525(7569):367–71.

Lelieveld J, Pozzer A, Pöschl U, Fnais M, Haines A, Münzel T. Loss of life expectancy from air pollution compared to other risk factors: a worldwide perspective. Cardiovasc Res. 2020 Sep 1;116(11):1910–7.

Lenton TM, Held H, Kriegler E, Hall JW, Lucht W, Rahmstorf S, et al. Tipping elements in the Earth's climate system. Proc Natl Acad Sci U S A. 2008 Feb 12;105(6):1786–93.

Li H, Huang G, Meng Q, Ma L, Yuan L, Wang F, et al. Integrated soil and plant phosphorus management for crop and environment in China. A review. Plant Soil. 2011 Dec 1;349(1):157–67.

Liu J, Dietz T, Carpenter SR, Alberti M, Folke C, Moran E, et al. Complexity of coupled human and natural systems. Science. 2007 Sep 14;317(5844):1513–6.

Liu X, Liu W, Liu L, Tang Q, Liu J, Yang H. Environmental flow requirements largely reshape global surface water scarcity assessment. Environ Res Lett. 2021 Oct 5;16(10):104029.

Locke H. Nature needs half: a necessary and hopeful new agenda for protected areas. Nature New South Wales. 2014;58(3):7–17.

Loisel H, Mangin A, Vantrepotte V, Dessailly D, Ngoc Dinh D, Garnesson P, et al. Variability of suspended particulate matter concentration in coastal waters under the Mekong's influence from ocean color (MERIS) remote sensing over the last decade. Remote Sens Environ. 2014 Jul 1;150:218–30.

Lytle DA, Poff NL. Adaptation to natural flow regimes. Trends Ecol Evol. 2004 Feb;19(2):94–100.

MacDonald GK, Bennett EM, Potter PA, Ramankutty N. Agronomic phosphorus imbalances across the world's croplands. Proc Natl Acad Sci U S A. 2011 Feb 15;108(7):3086–91.

Magnone D, Niasar VJ, Bouwman AF, Beusen AHW, van der Zee SEATM, Sattari SZ. Soil chemistry aspects of predicting future phosphorus requirements in sub-Saharan Africa. J Adv Model Earth Syst. 2019 Jan;11(1):327–37.

Mao J, Fu W, Shi X, Ricciuto DM, Fisher JB, Dickinson RE, et al. Disentangling climatic and anthropogenic controls on global terrestrial evapotranspiration trends. Environ Res Lett. 2015 Sep 8;10(9):094008.

Marchetti MP, Moyle PB. Effects of flow regime on fish assemblages in a regulated California stream. Ecol Appl. 2001 Apr;11(2):530–9.

Marcott SA, Shakun JD, Clark PU, Mix AC. A reconstruction of regional and global temperature for the past 11,300 years. Science. 2013 Mar 8;339(6124):1198–201.

Martínez-Salinas A, DeClerck F, Vierling K, Vierling L, Legal, Luc, Vílchez-Mendoza S, et al. Bird functional diversity supports pest control services in a Costa Rican coffee farm. Agric Ecosyst Environ. 2016 Nov 1;235:277–88.

Matthews TJ, Cottee-Jones HE, Whittaker RJ. Habitat fragmentation and the species–area relationship: a focus on total species richness obscures the impact of habitat loss on habitat specialists. Divers Distrib. 2014 Oct;20(10):1136–46.

Matthews TJ, Guilhaumon F, Triantis KA, Borregaard MK, Whittaker RJ, Santos A. On the form of species–area relationships in habitat islands and true islands. Glob Ecol Biogeogr. 2016 Jul;25(7):847–58.

McClain ME, Subalusky AL, Anderson EP, Dessu SB, Melesse AM, Ndomba PM, et al. Comparing flow regime, channel hydraulics, and biological communities to infer flow–ecology relationships in the Mara River of Kenya and Tanzania. Hydrol Sci J. 2014 Apr 3;59(3-4):801–19.

McIntyre PB, Reidy Liermann CA, Revenga C. Linking freshwater fishery management to global food security and biodiversity conservation. Proc Natl Acad Sci U S A. 2016 Nov 8;113(45):12880–5.

Mehrabi Z, Ellis EC, Ramankutty N. The challenge of feeding the world while conserving half the planet. Nature Sustainability. 2018 Aug 14;1(8):409–12.

Mekonnen MM, Hoekstra AY. Global Gray Water Footprint and Water Pollution Levels Related to Anthropogenic Nitrogen Loads to Fresh Water. Environ Sci Technol. 2015 Nov 3;49(21):12860–8.

Mekonnen MM, Hoekstra AY. Global anthropogenic phosphorus loads to freshwater and associated grey water footprints and water pollution levels: A high-resolution global study. Water Resour Res. 2018 Jan;54(1):345–58.

Meyer L. Intergenerational Justice. In: The Stanford Encyclopedia of Philosophy . Metaphysics Research Lab, Stanford University; 2021. Available from: https://plato.stanford.edu/archives/sum2021/entries/justice-intergenerational/

Mickley LJ, Jacob DJ, Field BD, Rind D. Effects of future climate change on regional air pollution episodes in the United States. Geophys Res Lett . 2004;31(24). Available from: http://doi.wiley.com/10.1029/2004GL021216

Ministry of Agriculture, Livestock, Fisheries and Cooperatives. National Agricultural Soil Management Policy . Republic of Kenya; 2020. Available from: https://kilimo.go.ke/wp-content/uploads/2021/01/Draft-National-Agricultural-Soil-Management-Policy-NASMP-September-2020.pdf

Moeck C, Grech-Cumbo N, Podgorski J, Bretzler A, Gurdak JJ, Berg M, et al. A global-scale dataset of direct natural groundwater recharge rates: A review of variables, processes and relationships. Sci Total Environ. 2020 May 15;717:137042.

Mohamed A, DeClerck F, Verburg PH, Obura D, Abrams JF, Zafra-Calvo N, et al. Biosphere functional integrity for people and planet . bioRxiv. 2022 [pre-print]. Available from: https://www.biorxiv.org/content/10.1101/2022.06.24.497294v3

Mohan, Western, Wei. Predicting groundwater recharge for varying land cover and climate conditions–a global meta-study. Hydrol Earth Syst Sci . 2018; Available from: https://hess.copernicus.org/articles/22/2689/2018/

Nakicenovic N, Rockström J, Gaffney O, Zimm C. Global Commons in the Anthropocene: World Development on a Stable and Resilient Planet. IIASA Working Paper . IIASA, Laxenburg, Austria, ; 2016. Report No.: WP-16-019. Available from: http://pure.iiasa.ac.at/14003/

Ndehedehe CE, Awange JL, Kuhn M, Agutu NO, Fukuda Y. Analysis of hydrological variability over the Volta river basin using in-situ data and satellite observations. Journal of Hydrology: Regional Studies. 2017 Aug 1;12:88–110.

Newbold T, Hudson LN, Arnell AP, Contu S, De Palma A, Ferrier S, et al. Has land use pushed terrestrial biodiversity beyond the planetary boundary? A global assessment. Science. 2016 Jul 15;353(6296):288–91.

Ney-Nifle M, Mangel M. Habitat loss and changes in the species-area relationship. Conserv Biol. 2000 Jun;14(3):893–8.

Nickel J. Human Rights. In: Zalta EN, editor. The Stanford Encyclopedia of Philosophy. Metaphysics Research Lab, Stanford University; 2021.

Norlock K. Feminist Ethics. In: Zalta EN, editor. The Stanford Encyclopedia of Philosophy . Summer 2019. Metaphysics Research Lab, Stanford University; 2019. Available from: https://plato.stanford.edu/archives/sum2019/entries/feminism-ethics/

Ochoa-Quintero JM, Gardner TA, Rosa I, Ferraz SF de B, Sutherland WJ. Thresholds of species loss in Amazonian deforestation frontier landscapes. Conserv Biol. 2015 Apr;29(2):440–51.

Ojha C, Werth S, Shirzaei M. Groundwater loss and aquifer system compaction in San Joaquin Valley during 2012-2015 drought. J Geophys Res [Solid Earth]. 2019 Mar;124(3):3127–43.

Okereke C. Global environmental sustainability: Intragenerational equity and conceptions of justice in multilateral environmental regimes. Geoforum. 2006 Sep 1;37(5):725–38.

O'Mara K, Venarsky M, Stewart-Koster B, McGregor GB, Schulz C, Kainz M, et al. Connectivity of fish communities in a tropical floodplain river system and predicted impacts of potential new dams. Sci Total Environ. 2021 Sep 20;788:147785.

O'Neill, van Aalst, Zaiton Ibrahim, et al. Key Risks Across Sectors and Regions. Chapter 16. In: H.-O. Pörtner, D.C. Roberts, M. Tignor, E.S. Poloczanska, K. Mintenbeck, A. Alegría, M. Craig, S. Langsdorf, S. Löschke, V. Möller, A. Okem, B. Rama, editor. Climate Change 2022: Impacts, Adaptation, and Vulnerability Contribution of Working Group II to the Sixth Assessment Report of the Intergovernmental Panel on Climate Change (IPCC). Cambridge University Press, Cambridge, UK and New York, NY, USA; 2022. p. 2411–538.

Orr S, Pittock J, Chapagain A, Dumaresq D. Dams on the Mekong River: Lost fish protein and the implications for land and water resources. Glob Environ Change. 2012 Oct 1;22(4):925–32.

Osman MB, Tierney JE, Zhu J, Tardif R, Hakim GJ, King J, et al. Globally resolved surface temperatures since the Last Glacial Maximum. Nature. 2021 Nov;599(7884):239–44.

Ovaskainen O, Hanski I. Extinction threshold in metapopulation models. Ann Zool Fennici. 2003;40(2):81–97.

Pan X. Application of fundamental equations to species-area theory. BMC Ecol. 2016 Oct 7;16(1):42.

Pardini R, Bueno A de A, Gardner TA, Prado PI, Metzger JP. Beyond the fragmentation threshold hypothesis: regime shifts in biodiversity across fragmented landscapes. PLoS One. 2010 Oct 27;5(10):e13666.

Parmesan, Morecroft, Trisurat, et al. Terrestrial and Freshwater Ecosystems and their Services. Chapter 2. In: H.-O. Pörtner, D.C. Roberts, M. Tignor, E.S. Poloczanska, K. Mintenbeck, A. Alegría, M. Craig, S. Langsdorf, S. Löschke, V. Möller, A. Okem, B. Rama, editor. Climate Change 2022: Impacts, Adaptation, and Vulnerability Contribution of Working Group II to the Sixth Assessment Report of the Intergovernmental Panel on Climate Change (IPCC). Cambridge University Press, Cambridge, UK and New York, NY, USA; 2022. p. 197–377.

Pastor AV, Ludwig F, Biemans H, Hoff H, Kabat P. Accounting for environmental flow requirements in global water assessments. Hydrol Earth Syst Sci. 2014 Dec 11;18(12):5041–59.

Pelicice FM, Pompeu PS, Agostinho AA. Large reservoirs as ecological barriers to downstream movements of Neotropical migratory fish. Fish Fish. 2014;16(4):697–715.

Pflüger FJ, Signer J, Balkenhol N. Habitat loss causes non-linear genetic erosion in specialist species. Global Ecology and Conservation. 2019 Jan 1;17:e00507.

Poff NL. Beyond the natural flow regime? Broadening the hydro-ecological foundation to meet environmental flows challenges in a non-stationary world. Freshw Biol. 2018 Aug;63(8):1011–21.

Poff NL, Allan JD, Bain MB, Karr JR, Prestegaard KL, Richter BD, et al. The Natural Flow Regime. Bioscience. 1997 Dec 1;47(11):769–84.

Poff NL, Olden JD, Merritt DM, Pepin DM. Homogenization of regional river dynamics by dams and global biodiversity implications. Proc Natl Acad Sci U S A. 2007 Apr 3;104(14):5732–7.

Poff NL, Richter BD, Arthington AH, Bunn SE, Naiman RJ, Kendy E, et al. The ecological limits of hydrologic alteration (ELOHA): a new framework for developing regional environmental flow standards. Freshw Biol. 2010 Jan;55(1):147–70.

Poff NL, Tharme RE, Arthington AH. Chapter 11 - Evolution of Environmental Flows Assessment Science, Principles, and Methodologies. In: Horne AC, Webb JA, Stewardson MJ, Richter B, Acreman M, editors. Water for the Environment. Academic Press; 2017. p. 203–36.

Poff NL, Zimmerman JKH. Ecological responses to altered flow regimes: a literature review to inform the science and management of environmental flows. Freshw Biol. 2010 Jan;55(1):194–205.

Postel SL, Daily GC, Ehrlich PR. Human Appropriation of Renewable Fresh Water. Science. 1996;271(5250):785–8.

Potts SG, Imperatriz-Fonseca V, Ngo HT, Aizen MA, Biesmeijer JC, Breeze TD, et al. Safeguarding pollinators and their values to human well-being. Nature. 2016 Dec 8;540(7632):220–9.

Poulhès A, Proulhac L. Exposed to NO2 in the center, NOx polluters in the periphery: Evidence from the Paris region. Sci Total Environ. 2022 May 15;821:153476.

Pozzer A, Tsimpidi AP, Karydis VA, de Meij A, Lelieveld J. Impact of agricultural emission reductions on fine-particulate matter and public health. Atmos Chem Phys. 2017 Oct 27;17(20):12813–26.

Prüss-Ustün A, Wolf J, Bartram J, Clasen T, Cumming O, Freeman MC, et al. Burden of disease from inadequate water, sanitation and hygiene for selected adverse health outcomes: An updated analysis with a focus on low- and middle-income countries. Int J Hyg Environ Health. 2019 Jun;222(5):765–77.

Quaas J, Jia H, Smith C, Albright AL, Aas W, Bellouin N, et al. Robust evidence for reversal of the trend in aerosol effective climate forcing. Atmos Chem Phys. 2022;22(18):12221–39.

Raaschou-Nielsen O, Taj T, Poulsen AH, Hvidtfeldt UA, Ketzel M, Christensen JH, et al. Air pollution at the residence of Danish adults, by socio-demographic characteristics, morbidity, and address level characteristics. Environ Res. 2022 May 15;208:112714.

Rammelt CF, Gupta J, Liverman D, Scholtens J, Ciobanu D, Abrams JF, et al. Impacts of meeting minimum access on critical earth systems amidst the Great Inequality. Nature Sustainability. 2022 Nov 10;1–10.

Rao ND, Kiesewetter G, Min J, Pachauri S, Wagner F. Household contributions to and impacts from air pollution in India. Nature Sustainability. 2021 Jul 26;4(10):859–67.

Recknagel T, Dornblut I, Looser U. Freshwater Fluxes into the World's Oceans. bfg Mitteilungen . Koblenz, Bundesanstalt für Gewässerkunde; 2021. Report No.: 36. Available from: http://dx.doi.org/10.5675/BfG_Mitteilungen_36.2021

Reinecke R, Müller Schmied H, Trautmann T, Andersen LS, Burek P, Flörke M, et al. Uncertainty of simulated groundwater recharge at different global warming levels: a global-scale multi-model ensemble study. Hydrol Earth Syst Sci. 2021;25(2):787–810.

Richter BD, Davis MM, Apse C, Konrad C. A presumptive standard for environmental flow protection. River Res Appl. 2012 Oct;28(8):1312–21.

Ricketts TH. Tropical forest fragments enhance pollinator activity in nearby coffee crops. Conserv Biol. 2004 Oct;18(5):1262–71.

Ricketts TH, Regetz J, Steffan-Dewenter I, Cunningham SA, Kremen C, Bogdanski A, et al. Landscape effects on crop pollination services: are there general patterns? Ecol Lett. 2008 May;11(5):499–515.

Riggio J, Baillie JEM, Brumby S, Ellis E, Kennedy CM, Oakleaf JR, et al. Global human influence maps reveal clear opportunities in conserving Earth's remaining intact terrestrial ecosystems. Glob Chang Biol. 2020 Aug;26(8):4344–56.

Rignot E, Mouginot J, Morlighem M, Seroussi H, Scheuchl B. Widespread, rapid grounding line retreat of Pine Island, Thwaites, Smith, and Kohler glaciers, West Antarctica, from 1992 to 2011. Geophys Res Lett. 2014 May 28;41(10):3502–9.

Rijsberman FR, Swart RJ (eds). Targets and Indicators of Climate Change. Report of Working Group II of the Advisory Group on Greenhouse Gases. Rijsberman FR, Swart RJ, editors. Stockholm Environmental Institute;

1990.

Ritchie PDL, Clarke JJ, Cox PM, Huntingford C. Overshooting tipping point thresholds in a changing climate. Nature. 2021 Apr;592(7855):517–23.

Rocha M, Krapp M, Guetschow J, Jeffery L, Hare B, Schaeffer M. Historical Responsibility for Climate Change - from countries emissions to contribution to temperature increase . Climate Analytics; 2015. Available from: https://www.climateanalytics.org/media/historical_responsibility_report_nov_2015.pdf

Rockström J, Gupta J, Lenton TM, Qin D, Lade SJ, Abrams JF, et al. Identifying a safe and just corridor for people and the planet. Earth’s Future . 2021 Apr;9(4). Available from: https://agupubs.onlinelibrary.wiley.com/doi/abs/10.1029/2020EF001866

Rockström J, Steffen W, Noone K, Persson A, Chapin FS 3rd, Lambin EF, et al. A safe operating space for humanity. Nature. 2009 Sep 24;461(7263):472–5.

Rolls RJ, Arthington AH. How do low magnitudes of hydrologic alteration impact riverine fish populations and assemblage characteristics? Ecol Indic. 2014 Apr 1;39:179–88.

Rosenfeld JS. Developing flow-ecology relationships: Implications of nonlinear biological responses for water management. Freshw Biol. 2017 Aug;62(8):1305–24.

Rost S, Gerten D, Bondeau A, Lucht W, Rohwer J, Schaphoff S. Agricultural green and blue water consumption and its influence on the global water system. Water Resour Res . 2008 Sep 1;44(9). Available from: https://doi.org/10.1029/2007WR006331

Rybicki J, Hanski I. Species-area relationships and extinctions caused by habitat loss and fragmentation. Ecol Lett. 2013 May;16 Suppl 1:27–38.

SBTN. Science-based targets for nature: initial guidance for business . Science Based Targets Network; 2020. Available from: https://sciencebasedtargetsnetwork.org/wp-content/uploads/2020/09/SBTN-initial-guidance-for-business.pdf

Schaphoff, Bloh, Rammig. LPJmL4–a dynamic global vegetation model with managed land–Part 1: Model description. Geosci Model Dev . 2018; Available from: https://gmd.copernicus.org/articles/11/1343/2018/

Schleicher J, Zaehringer JG, Fastré C, Vira B, Visconti P, Sandbrook C. Protecting half of the planet could directly affect over one billion people. Nature Sustainability. 2019 Nov 18;2(12):1094–6.

Schleussner CF, Lissner TK, Fischer EM, Wohland J, Perrette M, Golly A, et al. Differential climate impacts for policy-relevant limits to global warming: the case of 1.5 °C and 2 °C. Earth System Dynamics. 2016 Apr 21;7(2):327–51.

Schulte-Uebbing LF, Beusen AHW, Bouwman AF, de Vries W. From planetary to regional boundaries for agricultural nitrogen pollution. Nature. 2022 Oct;610(7932):507–12.

Schulte-Uebbing L, de Vries W. Reconciling food production and environmental boundaries for nitrogen in the European Union. Sci Total Environ. 2021 Sep 10;786:147427.

Seitzinger, S P , Mayorga, E , Bouwman, A F , Kroeze, C , Beusen, A H , Billen, G , Van Drecht, G. , Dumont, E. , Fekete, B. M. , Garnier, J. , Harrison, J. A. Global river nutrient export: A scenario analysis of past and future trends. Global Biogeochemical Cycles . 2010;24(4). Available from: https://agupubs.onlinelibrary.wiley.com/doi/full/10.1029/2009GB003587

Sirami C, Gross N, Baillod AB, Bertrand C, Carrié R, Hass A, et al. Increasing crop heterogeneity enhances multitrophic diversity across agricultural regions. Proc Natl Acad Sci U S A. 2019 Aug 13;116(33):16442–7.

Skillington T. Climate change and intergenerational justice. Routledge; 2019.

Sperna Weiland FC, van Beek LPH, Kwadijk JCJ, Bierkens MFP. The ability of a GCM-forced hydrological model to reproduce global discharge variability. Hydrol Earth Syst Sci. 2010 Aug 19;14(8):1595–621.

Springmann M, Clark M, Mason-D’Croz D, Wiebe K, Bodirsky BL, Lassaletta L, et al. Options for keeping the food system within environmental limits. Nature. 2018 Oct;562(7728):519–25.

Steffan-Dewenter I, Münzenberg U, Bürger C, Thies C, Tscharntke T. Scale-dependent effects of landscape context on three pollinator guilds. Ecology. 2002 May;83(5):1421–32.

Steffan-Dewenter I, Tscharntke T. Effects of habitat isolation on pollinator communities and seed set. Oecologia. 1999 Nov;121(3):432–40.

Steffen W, Richardson K, Rockström J, Cornell SE, Fetzer I, Bennett EM, et al. Planetary boundaries: guiding human development on a changing planet. Science. 2015;347(6223):1259855.

Steffen W, Richardson K, Rockström J, Schellnhuber HJ, Dube OP, Dutreuil S, et al. The emergence and evolution of Earth System Science. Nature Reviews Earth & Environment. 2020 Jan 13;1(1):54–63.

Sterling SM, Ducharne A, Polcher J. The impact of global land-cover change on the terrestrial water cycle. Nat Clim Chang. 2013;3(4):385–90.

Stewart ZP, Pierzynski GM, Middendorf BJ, Prasad PVV. Approaches to improve soil fertility in sub-Saharan Africa. J Exp Bot. 2020 Jan 7;71(2):632–41.

Strassburg BBN, Iribarrem A, Beyer HL, Cordeiro CL, Crouzeilles R, Jakovac CC, et al. Global priority areas for ecosystem restoration. Nature. 2020 Oct;586(7831):724–9.

Sutton MA, Bleeker A, Howard CM et al. Our Nutrient World: The challenge to produce more food and energy with less pollution. Global Overview of Nutrient Management . Centre for Ecology and Hydrology, Edinburgh on behalf of the Global Partnership on Nutrient Management and the International Nitrogen Initiative.; 2013. Available from: https://wedocs.unep.org/handle/20.500.11822/10747

Swift TL, Hannon SJ. Critical thresholds associated with habitat loss: a review of the concepts, evidence, and applications. Biol Rev Camb Philos Soc. 2010 Feb;85(1):35–53.

Syvitski JPM, Kettner AJ, Overeem I, Hutton EWH, Hannon MT, Brakenridge GR, et al. Sinking deltas due to human activities. Nat Geosci. 2009 Sep 20;2(10):681–6.

Tessler ZD, Vörösmarty CJ, Overeem I, Syvitski JPM. A model of water and sediment balance as determinants of relative sea level rise in contemporary and future deltas. Geomorphology . 2018 Mar 15;305:209–20.

Tharme RE. A global perspective on environmental flow assessment: emerging trends in the development and application of environmental flow methodologies for rivers. River Res Appl. 2003 Sep;19(5-6):397–441.

Thies C, Steffan-Dewenter I, Tscharntke T. Effects of landscape context on herbivory and parasitism at different spatial scales. Oikos. 2003 Apr;101(1):18–25.

Thomas BF, Famiglietti JS. Identifying Climate-Induced Groundwater Depletion in GRACE Observations. Sci Rep. 2019 Mar 11;9(1):4124.

Tickner D, Opperman JJ, Abell R, Acreman M, Arthington AH, Bunn SE, et al. Bending the Curve of Global Freshwater Biodiversity Loss: An Emergency Recovery Plan. Bioscience. 2020 Apr 1;70(4):330–42.

Tignino M, Bréthaut C. The role of international case law in implementing the obligation not to cause significant harm. International Environmental Agreements: Politics, Law and Economics. 2020 Dec 1;20(4):631–48.

Tirado R, Allsopp M. Phosphorus in agriculture: problems and solutions. Technical Report (Review) . Greenpeace Research Laboratories ; 2012. Report No.: 2. Available from: https://www.greenpeace.to/greenpeace/wp-content/uploads/2012/06/tirado-and-allsopp-2012-phosphorus-in-agriculture-technical-report-02-2012.pdf

Trenberth KE, Fasullo JT, Mackaro J. Atmospheric Moisture Transports from Ocean to Land and Global Energy Flows in Reanalyses. J Clim. 2011 Sep 15;24(18):4907–24.

Triantis KA, Borges PAV, Ladle RJ, Hortal J, Cardoso P, Gaspar C, et al. Extinction debt on oceanic islands. Ecography . 2010 May;no – no.

Triantis KA, Guilhaumon F, Whittaker RJ. The island species-area relationship: biology and statistics. J Biogeogr. 2012 Feb;39(2):215–31.

Tscharntke T, Grass I, Wanger TC, Westphal C, Batáry P. Beyond organic farming - harnessing biodiversity-friendly landscapes. Trends Ecol Evol. 2021 Oct;36(10):919–30.

Tscharntke T, Karp DS, Chaplin-Kramer R, Batáry P, DeClerck F, Gratton C, et al. When natural habitat fails to enhance biological pest control – Five hypotheses. Biol Conserv. 2016 Dec 1;204:449–58.

Tscharntke T, Tylianakis JM, Rand TA, Didham RK, Fahrig L, Batáry P, et al. Landscape moderation of biodiversity patterns and processes - eight hypotheses. Biol Rev Camb Philos Soc. 2012 Aug;87(3):661–85.

Turschwell MP, Stewart-Koster B, King AJ, Pusey B, Crook D, Boone E, et al. Flow-mediated predator–prey dynamics influence fish populations in a tropical river. Freshw Biol. 2019 Aug;64(8):1453–66.

Ulrich W, Buszko J. Species-area relationships of butterflies in Europe and species richness forecasting. Ecography . 2003 Jun;26(3):365–73.

UNCCD. The Global Land Outlook . Second. United Nations Convention to Combat Desertification, Bonn.; 2022. Available from: https://www.unccd.int/sites/default/files/2022-04/UNCCD_GLO2_low-res_2.pdf

UNEP. Global Chemicals Outlook II - From Legacies to Innovative Solutions: Implementing the 2030 Agenda for Sustainable Development . United Nations Environment Programme; 2019. Available from: https://www.unep.org/explore-topics/chemicals-waste/what-we-do/policy-and-governance/global-chemicals-outlook

UN GA. Transforming our world: the 2030 Agenda for Sustainable Development, available from . Vol. A/RES/70/1 (25 September 2015)., General Assembly resolution 70/1. 2015. Available from: https://undocs.org/en/A/RES/70/1

Van Binh D, Kantoush S, Sumi T. Changes to long-term discharge and sediment loads in the Vietnamese Mekong Delta caused by upstream dams. Geomorphology . 2020 Mar 15;353:107011.

Van Drecht G, Bouwman AF, Harrison J, Knoop JM. Global nitrogen and phosphate in urban wastewater for the period 1970 to 2050. Global Biogeochem Cycles . 2009 Dec;23(4). Available from: http://doi.wiley.com/10.1029/2009GB003458

Van Grinsven HJM, Holland M, Jacobsen BH, Klimont Z, Sutton MA, Jaap Willems W. Costs and benefits of nitrogen for Europe and implications for mitigation. Environ Sci Technol. 2013 Apr 16;47(8):3571–9.

Visioni D, MacMartin DG, Kravitz B, Richter JH, Tilmes S, Mills MJ. Seasonally modulated stratospheric aerosol geoengineering alters the climate outcomes. Geophys Res Lett . 2020 Jun 28;47(12). Available from: https://onlinelibrary.wiley.com/doi/10.1029/2020GL088337

Vogel A, Alessa G, Scheele R, Weber L, Dubovik O, North P, et al. Uncertainty in aerosol optical depth from modern aerosol-climate models, reanalyses, and satellite products. J Geophys Res . 2022 Jan 27;127(2). Available from: https://onlinelibrary.wiley.com/doi/10.1029/2021JD035483

de Vrese P, Stacke T, Kleinen T, Brovkin V. Diverging responses of high-latitude CO_2 and CH_4 emissions in idealized climate change scenarios. cryosphere. 2021 Mar 2;15(2):1097–130.

de Vries W. Impacts of nitrogen emissions on ecosystems and human health: A mini review. Current Opinion in Environmental Science & Health. 2021 Jun 1;21:100249.

de Vries W, Kros J, Kroeze C, Seitzinger SP. Assessing planetary and regional nitrogen boundaries related to food security and adverse environmental impacts. Current Opinion in Environmental Sustainability. 2013 Sep 1;5(3):392–402.

de Vries W, Schulte-Uebbing L, Kros H, Voogd JC, Louwagie G. Spatially explicit boundaries for agricultural nitrogen inputs in the European Union to meet air and water quality targets. Sci Total Environ. 2021 Sep 10;786:147283.

Wada Y, van Beek LPH, van Kempen CM, Reckman JWTM, Vasak S, Bierkens MFP. Global depletion of groundwater resources. Geophys Res Lett . 2010 Oct;37(20). Available from: http://doi.wiley.com/10.1029/2010GL044571

Wang-Erlandsson L, Tobian A, van der Ent RJ, Fetzer I, te Wierik S, Porkka M, et al. A planetary boundary for green water. Nature Reviews Earth & Environment. 2022 Apr 26;1–13.

Wang J, Dong Z, Liao W, Li C, Feng S, Luo H, et al. An environmental flow assessment method based on the relationships between flow and ecological response: A case study of the Three Gorges Reservoir and its downstream reach. Sci China Tech Sci. 2013 Jun 1;56(6):1471–84.

Wang L, Good SP, Caylor KK. Global synthesis of vegetation control on evapotranspiration partitioning. Geophys Res Lett. 2014 Oct 16;41(19):6753–7.

Ward MH, Jones RR, Brender JD, de Kok TM, Weyer PJ, Nolan BT, et al. Drinking Water Nitrate and Human Health: An Updated Review. Int J Environ Res Public Health . 2018 Jul 23;15(7). Available from: http://dx.doi.org/10.3390/ijerph15071557

Watkins MM, Wiese DN, Yuan DN, Boening C, Landerer FW. Improved methods for observing Earth’s time variable mass distribution with GRACE using spherical cap mascons. J Geophys Res [Solid Earth]. 2015 Apr;120(4):2648–71.

Watson AJ, Lenton TM, Mills BJW. Ocean deoxygenation, the global phosphorus cycle and the possibility of human-caused large-scale ocean anoxia. Philos Trans A Math Phys Eng Sci . 2017 Sep 13;375(2102). Available from: http://dx.doi.org/10.1098/rsta.2016.0318

Watts N, Amann M, Arnell N, Ayeb-Karlsson S, Belesova K, Boykoff M, et al. The 2019 report of The Lancet

Countdown on health and climate change: ensuring that the health of a child born today is not defined by a changing climate. Lancet. 2019 Nov 16;394(10211):1836–78.

Webb AJ, Miller KA, King EL, Little SC, Stewardson MJ, Zimmerman JKH, et al. Squeezing the most out of existing literature: a systematic re-analysis of published evidence on ecological responses to altered flows. Freshw Biol. 2013 Dec;58(12):2439–51.

Westervelt DM, Horowitz LW, Naik V, Tai APK, Fiore AM, Mauzerall DL. Quantifying PM2.5-meteorology sensitivities in a global climate model. Atmos Environ. 2016 Oct 1;142:43–56.

WHO. Air quality guidelines. Global update 2005. Particulate matter, ozone, nitrogen dioxide, and sulfur dioxide . World Health Organization. Europe; 2006. Available from: https://www.euro.who.int/__data/assets/pdf_file/0005/78638/E90038.pdf

WHO. Guidelines for drinking-water quality. Fourth edition incorporating the first addendum. World Heath Organization; 2017.

WHO. Ambient (outdoor) air pollution . 2021 [cited 2022 Jun 6]. Available from: https://www.who.int/news-room/fact-sheets/detail/ambient-(outdoor)-air-quality-and-health

Willeit M, Ganopolski A, Calov R, Brovkin V. Mid-Pleistocene transition in glacial cycles explained by declining CO_2 and regolith removal. Sci Adv. 2019 Apr;5(4):eaav7337.

Willett W, Rockström J, Loken B, Springmann M, Lang T, Vermeulen S, et al. Food in the Anthropocene: the EAT-Lancet Commission on healthy diets from sustainable food systems. Lancet. 2019 Feb 2;393(10170):447–92.

Wilson EO. Half-Earth: Our Planet's Fight for Life. W. W. Norton & Company; 2016.

Winkelmann R, Martin MA, Haseloff M, Albrecht T, Bueler E, Khroulev C, et al. The Potsdam Parallel Ice Sheet Model (PISM-PIK) – Part 1: Model description. The Cryosphere. 2011;5(3):715–26.

Winkler AJ, Myneni RB, Hannart A, Sitch S, Haverd V, Lombardozzi D, et al. Slowdown of the greening trend in natural vegetation with further rise in atmospheric CO_2. Biogeosciences. 2021 Sep 13;18(17):4985–5010.

Wisser D, Fekete BM, Vörösmarty CJ, Schumann AH. Reconstructing 20th century global hydrography: a contribution to the Global Terrestrial Network- Hydrology (GTN-H). Hydrol Earth Syst Sci. 2010 Jan 6;14(1):1–24.

With KA, King AW. Extinction Thresholds for Species in Fractal Landscapes. Conserv Biol. 1999 Apr 1;13(2):314–26.

Woodley S, Locke H, Laffoley D, MacKinnon K, Sandwith T, Smart J. A review of evidence for area-based conservation targets for the post-2020 global biodiversity framework. Parks. 2019 Dec 5;25(2):31–46.

Wunderling N, Winkelmann R, Rockström J, Loriani S, Armstrong-McKay D, Ritchie P, et al. Global warming overshoots increase risk of triggering climate tipping points and cascades. Nat Clim Chang . 2022; Available from: https://www.researchsquare.com/article/rs-1418830/latest.pdf

Yeakley AJ, Ervin D, Chang H, Granek EF, Dujon V, Shandas V, et al. Ecosystem services of streams and rivers. In: River Science. Chichester, UK: John Wiley & Sons, Ltd; 2016. p. 335–52.

Zhang X, Davidson EA, Mauzerall DL, Searchinger TD, Dumas P, Shen Y. Managing nitrogen for sustainable development. Nature. 2015 Dec 3;528(7580):51–9.

Zhang X, Yao G, Vishwakarma S, Dalin C, Komarek AM, Kanter DR, et al. Quantitative assessment of agricultural sustainability reveals divergent priorities among nations. One Earth. 2021a Sep 17;4(9):1262–77.

Zhang X, Zou T, Lassaletta L, Mueller ND, Tubiello FN, Lisk MD, et al. Quantification of global and national nitrogen budgets for crop production. Nature Food. 2021b Jul 15;2(7):529–40.

Zhang Y, Pan M, Sheffield J, Siemann AL, Fisher CK, Liang M, et al. A Climate Data Record (CDR) for the global terrestrial water budget: 1984–2010. Hydrol Earth Syst Sci. 2018 Jan 12;22(1):241–63.

Zhao M, Cao L, Bala G, Duan L. Climate response to latitudinal and altitudinal distribution of stratospheric sulfate aerosols. J Geophys Res . 2021 Dec 27;126(24). Available from: https://onlinelibrary.wiley.com/doi/10.1029/2021JD035379

Zipper SC, Jaramillo F, Wang-Erlandsson L, Cornell SE, Gleeson T, Porkka M, et al. Integrating the Water Planetary Boundary With Water Management From Local to Global Scales. Earth's Future. 2020 Feb;8(2):e2019EF001377.

Zou T, Zhang X, Davidson EA. Global trends of cropland phosphorus use and sustainability challenges. Nature. 2022 Nov;611(7934):81–7.
