# Supplementary material for: Safe and just Earth system boundaries
Source: Nature. 2023 May 31;619(7968):102–11. doi: 10.1038/s41586-023-06083-8 (PMC10322705; doi:10.1038/s41586-023-06083-8)
Supplement: Supplementary file 2 — Reporting Summary [file 41586_2023_6083_MOESM2_ESM.pdf]

nature portfolio

Corresponding author(s): Johan Rockström and Steven Lade

Last updated by author(s): Mar 10, 2023

Reporting Summary

Nature Portfolio wishes to improve the reproducibility of the work that we publish. This form provides structure for consistency and transparency in reporting. For further information on Nature Portfolio policies, see our Editorial Policies and the Editorial Policy Checklist.

Statistics

For all statistical analyses, confirm that the following items are present in the figure legend, table legend, main text, or Methods section.

n/a	Confirmed	
☐	☒	The exact sample size (*n*) for each experimental group/condition, given as a discrete number and unit of measurement
☒	☐	A statement on whether measurements were taken from distinct samples or whether the same sample was measured repeatedly
☒	☐	The statistical test(s) used AND whether they are one- or two-sided<br>*Only common tests should be described solely by name; describe more complex techniques in the Methods section.*
☒	☐	A description of all covariates tested
☒	☐	A description of any assumptions or corrections, such as tests of normality and adjustment for multiple comparisons
☐	☒	A full description of the statistical parameters including central tendency (e.g. means) or other basic estimates (e.g. regression coefficient) AND variation (e.g. standard deviation) or associated estimates of uncertainty (e.g. confidence intervals)
☒	☐	For null hypothesis testing, the test statistic (e.g. *F*, *t*, *r*) with confidence intervals, effect sizes, degrees of freedom and *P* value noted<br>*Give P values as exact values whenever suitable.*
☒	☐	For Bayesian analysis, information on the choice of priors and Markov chain Monte Carlo settings
☒	☐	For hierarchical and complex designs, identification of the appropriate level for tests and full reporting of outcomes
☒	☐	Estimates of effect sizes (e.g. Cohen's *d*, Pearson's *r*), indicating how they were calculated

Our web collection on statistics for biologists contains articles on many of the points above.

Software and code

Policy information about availability of computer code

Data collection	No software used for data collection.
Data analysis	Software used includes: R version 4.1.3 (Figures 2 and 3), Google Sheets (accessed in 2022, to track papers for functional integrity literature review), Google Earth Engine (accessed in 2022, for current state of functional integrity), Python v3.7.6 using Jupyter notebook web-based interactive computing platform v 6.2.0 (development of environmental flows and surface water boundaries raster datasets), QGIS v3.22.9 (for data review of surface water boundary), Water Balance Model (accessed in 2022), MATLAB R2018A (for processing the GRACE data and GLDAS model output for the groundwater boundary).

For manuscripts utilizing custom algorithms or software that are central to the research but not yet described in published literature, software must be made available to editors and reviewers. We strongly encourage code deposition in a community repository (e.g. GitHub). See the Nature Portfolio guidelines for submitting code & software for further information.

Data

Policy information about availability of data

All manuscripts must include a data availability statement. This statement should provide the following information, where applicable:

- Accession codes, unique identifiers, or web links for publicly available datasets
- A description of any restrictions on data availability
- For clinical datasets or third party data, please ensure that the statement adheres to our policy

Please see data availability statement in manuscript.

Human research participants

Policy information about studies involving human research participants and Sex and Gender in Research.

Reporting on sex and gender	N/A
Population characteristics	N/A
Recruitment	N/A
Ethics oversight	N/A

Note that full information on the approval of the study protocol must also be provided in the manuscript.

Field-specific reporting

Please select the one below that is the best fit for your research. If you are not sure, read the appropriate sections before making your selection.

☐ Life sciences ☐ Behavioural & social sciences ☒ Ecological, evolutionary & environmental sciences

For a reference copy of the document with all sections, see nature.com/documents/nr-reporting-summary-flat.pdf

Ecological, evolutionary & environmental sciences study design

All studies must disclose on these points even when the disclosure is negative.

Study description	Methodologies for each domain is included in text, see Methods and Supplementary Methods. These methodologies varied by domain, including relying upon recently published literature review, recently published modelling, new literature review, and new analysis based on well-established datasets. We here include the new literature review for the functional integrity boundary as an example.
Research sample	Web of Science
Sampling strategy	Search terms as described in Supplementary Methods, Table S2.
Data collection	Awaz Mohamed ran the searches.
Timing and spatial scale	Limited to papers between 2010 and 2021 (see Supplementary Methods).
Data exclusions	Papers were filtered in several steps (Supplementary Methods), including "Of those [402 potentially eligible] studies we conducted a full text review to confirm eligibility which was established if the reference contained measures of habitat quantity within 1 km at the landscape scale with measures appearing either directly in the source text, tables, supplementary information or in figures."
Reproducibility	The functional integrity boundary for several NCPs were studied and compared (Table S2).
Randomization	N/A
Blinding	N/A

Did the study involve field work? ☐ Yes ☒ No

Reporting for specific materials, systems and methods

We require information from authors about some types of materials, experimental systems and methods used in many studies. Here, indicate whether each material, system or method listed is relevant to your study. If you are not sure if a list item applies to your research, read the appropriate section before selecting a response.

Materials & experimental systems

n/a	Involved in the study
☒	☐ Antibodies
☒	☐ Eukaryotic cell lines
☒	☐ Palaeontology and archaeology
☒	☐ Animals and other organisms
☒	☐ Clinical data
☒	☐ Dual use research of concern

Methods

n/a	Involved in the study
☒	☐ ChIP-seq
☒	☐ Flow cytometry
☒	☐ MRI-based neuroimaging
